# A synaptoid connectome differentiates tanycytic subpopulations and underlies neuroglial communication and neuroendocrine regulation

**DOI:** 10.1038/s41467-026-74598-5

**Published:** 2026-06-20

**Authors:** Vanessa Neve, Daniela Fernandois, Surya Rai, Ümit Özorhan, Sreekala Nampoothiri, Gaetan Ternier, Spyridon Sideromenos, Sarah Gallet, Cecile Allet, Marcus Stahr, Natascha Klaus, Ines Martinez-Corral, Caio Fernando Ferreira Coêlho, Akila Chandrasekar, Andreea Constantinescu, Manon Rivagorda, Simon Dührkop, Julia Cases Bazarra, Sonja Binder, Paolo Giacobini, Sowmyalakshmi Rasika, Ruben Nogueiras, Tibor Harkany, Martin K. Schwarz, Helge Müller-Fielitz, Vincent Prevot, Markus Schwaninger

**Affiliations:** 1 Institute of Experimental and Clinical Pharmacology and Toxicology, University of Luebeck, Luebeck, Germany; 2 University Lille, Inserm, CHU Lille, Laboratory of Development and Plasticity of the Neuroendocrine Brain, Lille Neuroscience & Cognition, UMR-S 1172, DISTALZ, EGID, Lille, France; 3 Department of Molecular Neurosciences, Center for Brain Research, Medical University of Vienna, Vienna, Austria; 4 Department of Physiology, CIMUS, University of Santiago de Compostela-Instituto de Investigación Sanitaria, Santiago de Compostela, Spain; 5 Department of Neuroscience, Karolinska Institutet, Solna, Sweden; 6 Institute for Experimental Epileptology and Cognition Research, University of Bonn Medical Center, Bonn, Germany

**Keywords:** Synaptic transmission, Reproductive biology, Glial biology

## Abstract

Tanycytes are radial-glia-like cells that play important roles in regulating the neuroendocrine system and metabolism. Synapse-like (synaptoid) connections have previously been described between neurons and tanycytes, but their structure and function are unclear. Here, we report that neuron-tanycyte synaptoids are abundant and resemble typical neuronal synapses in shape and composition. Tanycytic subtypes receive specific inputs from a variety of hypothalamic as well as extrahypothalamic neuronal populations and respond to several neurotransmitters and neuromodulators. As proof-of-principle of their functional relevance, we demonstrate in mice, that two distinct populations of kisspeptin neurons, which stimulate the gonadotropic axis, innervate different tanycytic subsets of the mediobasal hypothalamus to control basal levels of the gonadotropin luteinizing hormone (LH) and its pulsatile release pattern, in a sex‑ and region‑specific manner. Neuron-tanycyte synaptoid connections are thus widespread, diverse and functionally specific elements of hypothalamic neural circuits that play a key role in finetuning hormonal axes.

## Abstract (web summary)

The structure and function of neuron-tanycyte synaptoids are unclear. Here, the authors show that tanycytic subtypes receive specific input from hypothalamic and extra-hypothalamic neurons. Neuron-tanycyte synaptoids can fine-tune hormonal axes.

## Introduction

The function of the central nervous system depends on connections between its cells. Previous research on these connections has focused on synapses between neurons^1^. So fundamental is connectivity that it serves as the basis for defining cell types and has even enabled the identification of new types of neurons^2^. Among glial cells, astrocytes and microglia modulate, stabilize, or prune neuron-neuron synapses, but are not integrated into neural networks through synaptic junctions^3–5^. Unlike astrocytes and microglia, oligodendrocyte progenitor cells (OPCs) receive direct synaptic input from neurons, whereby neurons exert trophic effects on OPCs, influencing their proliferation and migration and the formation of myelin sheaths^6–9^. In addition to OPCs, neuronal processes also terminate on typical ependymal cells and on tanycytes^10^, specialized radial glia-like cells that line the wall of the third ventricle in the mediobasal hypothalamus and send long projections into several hypothalamic nuclei^11^. Based on their location in the ventricular wall and their cell shape, tanycytes can be differentiated into α-tanycytes near the dorsomedial and ventromedial hypothalamic nuclei (DMH, VMH) and β-tanycytes near the arcuate nucleus (ARC) and the median eminence (ME)^11^. Electron microscopy has demonstrated the existence of synapse-like, or “synaptoid”, nerve endings mainly on β-tanycytes^12–16^. However, more than half a century after the first description of synaptoid connections on tanycytes, our knowledge of their structure, function, and the exact identity of the afferent neurons remains limited.

Tanycytes are known to perform a number of unique functions. For instance, the endfeet of tanycytic projections cover blood vessels, including the fenestrated portal vessels of the median eminence. By regulating the size of their endfeet through a Ca^2+^-dependent mechanism, certain tanycytes control the access of neurosecretory nerve terminals to pituitary portal vessels^17^, and thus the blood-borne delivery of releasing hormones, such as gonadotropin-releasing hormone (GnRH), to the pituitary gland^18^. Consequently, tanycytes influence the secretion of pituitary hormones, including the gonadotropin luteinizing hormone (LH). Other functions of tanycytes have been attributed to their ability to form the blood-brain barrier, to transport metabolic hormones and nutrients from the blood into the cerebrospinal fluid (CSF), and to modulate the activity of neurons involved in regulating metabolism and endocrine rhythms^19–21^. Single-cell RNA sequencing (scRNAseq) studies have demonstrated the existence of additional tanycyte subpopulations, although the functional implications of this finding have been unclear^22–24^. Synaptoid contacts by neurons could thus be an optimal means to transmit the multitude of inputs that tanycytes need to fulfil their diverse tasks.

Here, we characterized the molecular composition and morphology of synaptoid connections between neurons and tanycytes. As these connections resemble typical neuron-neuron synapses, we sought to adapt the rabies-virus-based retrograde tracing technology that has been successfully applied to characterize neuron-neuron networks^25^ to these neuron-tanycyte connections. This technique successfully revealed a tanycytic connectome consisting of multiple neuronal populations that provide highly specific input to tanycytes. Interestingly, tanycytic subpopulations differ in the neurons to which they are connected and in the neurotransmitters and neuromodulators to which they respond. As proof-of-concept of the functional relevance of these connections, we studied the relative contribution of different tanycytic subpopulations to the release of GnRH and thus of LH, which occurs in the form of pulses in both males and females (with an additional preovulatory surge in females). We show that while neurons expressing kisspeptin, a powerful GnRH secretagogue, innervate all tanycytes, two different tanycytic populations mediate kisspeptin effects on the rhythm of GnRH/LH release and on basal LH levels, thus controlling hypothalamic-pituitary-gonad (HPG) axis function. Together, these results pinpoint the crucial role of tanycytes in neuroglial networks that regulate hormone axes and possibly other hypothalamic functions.

## Results

### Synaptoid connections on tanycytes

**Fig. 1:**
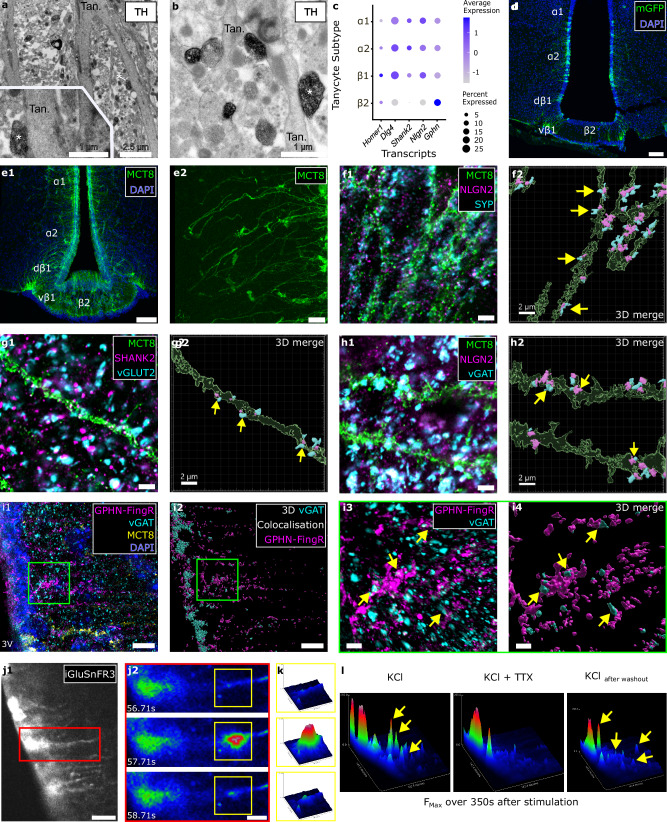
Synaptoid terminals on tanycytes. **a**, **b** Electron microscope images of tyrosine hydroxylase (TH)-positive neuronal endings (*) on tanycyte (Tan) in the mediobasal hypothalamus of mice. **c** ScRNAseq analyses indicate that tanycytes express genes of several postsynaptic proteins in adult mice (> 6 months old) on normal diet. **d** Sparse labeling of tanycyte plasma membranes with mGFP (green) after injection of the tanycyte-specific *AAV-Dio2-Cre* in *mT/mG* mice. The position of α1-, α2-, dorsal (**d**) and ventral (v) β1-, as well as β2-tanycytes on the coronal section are indicated. Scale bar, 100 µm. **e** Immunofluorescence staining of MCT8 enabled membrane labeling of tanycytes. Scale bars, **e1**, 100 µm; **e2**, 25 µm. **f–h** The pre-synaptic proteins (cyan) SYNAPTOPHYSIN (SYP, **f**), vGLUT2 (**g**), and vGAT (**h**) were closely apposed to the tanycyte post-synaptic proteins (magenta) NEUROLIGIN-2 (NLGN2, **f**, **h**) and SHANK2 (**g**). After immunofluorescence staining, MCT8 was imaged by confocal microscopy, and pre- and post-synaptic proteins by 3D-STED. 3D-image rendering (**f2**, **g2**, **h2**) showed that post-synaptoid- puncta in tanycyte plasma membranes lay in apposition to pre-synaptoid synaptophysin, vGAT, or vGLUT2, respectively. Scale bars, 2 µm. **i** Tanycyte GEPHYRIN (GPHN) was selectively labeled by expressing GPHN-FingR-GFP (magenta) in tanycytes. Immunofluorescence staining of vGAT (cyan, **i1**, **i3**) and 3D rendering (**i2**, **i4**) showed apposition of vGAT with tanycyte GPHN. Scale bars **i1**, **i2**, 10 µm; **i3**, i4, 5 µm. **j–l** Representative glutamate imaging in acute brain slices after transducing tanycytes with *AAV-CAG-iGluSnFR3*, encoding a fluorescent glutamate sensor. KCl (30 mM) stimulated glutamate release in tanycyte synaptoids, as shown by flashes of increased fluorescence close to tanycyte cell bodies and processes (**j**, **k**). This result was reproduced using slices from 8 mice. **l** A 3D surface plot, visualizing the maximal fluorescence over 350 s from (**j1**), after treatment with KCl with and without tetrodotoxin (TTX, 1 µM). The effect of KCl was abolished by TTX in a reversible manner. This result was reproduced using slices from 5 mice. For the image analysis, see Supplementary Fig. 3. For information on the sex of mice, see Supplementary Data File 5.

Using electron microscopy (EM), we confirmed that in the mediobasal hypothalamus of mice, synaptoid nerve terminals are closely apposed to tanycytes (Fig. 1a, b) and contain small pre-synaptic vesicles. Tanycytic synaptoids mostly lacked a post-synaptic density and thus resembled symmetric neuronal synapses^12–16^. To assess the molecular composition of the post-synaptoids in tanycytes, we analyzed scRNAseq data. For this, we merged existing scRNAseq information^22,23,26,27^ in the database Tanybase, which contains transcriptomic data from 20,610 mouse tanycytes. In line with the presence of synaptoid contacts, tanycytes expressed the genes of several post-synaptic proteins, including *Homer1*, *Dlg4* (gene for PSD95), *Shank2*, *Nlgn2*, and *Gephyrin* (*Gphn*, Fig. 1c). We detected the post-synaptic proteins PSD95 and NEUROLIGIN-2 (NLGN2) in primary tanycyte cultures by Western blotting (Supplementary Fig. 1a, b). In brain sections, we labeled the tanycytic plasma membrane by injecting the tanycyte-specific *AAV-Dio2-Cre* vector into *mT/mG* mice^17,28^ to express the membrane-bound GFP (mGFP) in tanycytes or by staining MCT8 (Fig. 1d, e and Supplementary Fig. 2a). Using this approach and confocal as well as super-resolution 3D-STED microscopy, we were able to detect the post-synaptic proteins GPHN, NLGN2, HOMER1, PSD95, and SHANK2 on the surface of tanycytes (Supplementary Fig. 2b–k)^29^. These post-synaptic proteins were found in small puncta overlapping with MCT8 or mGFP in the plasma membrane of tanycytes (Fig. 1f–h and Supplementary Fig. 2b–k). Optimal transport co-localization analysis^30^ of 3D-STED images demonstrated the relative proximity of SHANK2 and NLGN2 to MCT8 in the tanycytic membrane (Supplementary Fig. 2l, m). SHANK2- and NLGN2-positive puncta had a higher density in α1-tanycytes than in β2-tanycytes (Supplementary Fig. 1c–h). Taking the total cell surface into account (Supplementary Fig. 1i), we estimated that α1-tanycytes carry 3772 $\pm \,$1502 NLGN2- and 1068 $\pm \,$185 SHANK2-positive post-synaptoid puncta and β2-tanycytes 545 $\pm \,$76 NLGN2- and 11 $\pm \,$1 (mean ± SEM, *n* = 3 mice) SHANK2-positive puncta per cell, with the other two subtypes mostly displaying intermediate numbers of puncta (Supplementary Fig. 1j), i.e., of a similar order of magnitude as estimates of the number of synapses per neuron^31^. Puncta were mostly located along processes, with only a few on tanycytic cell bodies (Supplementary Fig. 1e, g). Notably, SHANK2 and NLGN2 differed in their distribution along α-tanycyte processes, with SHANK2-positive puncta decreasing and NLGN2-positive puncta increasing in the more distal fraction of tanycytic processes (Supplementary Fig. 1f, h). The size and shape of post-synaptoid junctions on tanycytes were similar to those of post-synaptic neuronal junctions.

Synaptophysin-positive pre-synaptic neuronal terminals are known to occur in close proximity to tanycytic processes^32^. Extending this observation, immunofluorescence labeling of the pre-synaptic proteins SYNAPTOPHYSIN, vGLUT2 (marker of glutamatergic pre-synapses) and vGAT (marker of GABAergic pre-synapses), together with the post-synaptic proteins SHANK2 and NLGN2, confirmed that pre-synaptic terminals were closely apposed to post-synaptoid tanycytic puncta (Fig. 1f–h). To verify that the post-synaptoid terminals are localized in tanycytes, we selectively expressed fibronectin intrabodies generated with mRNA display (FingR) directed against GPHN and PSD95 in tanycytes^33^. GPHN FingR-GFP and PSD95 FingR-GFP labeled puncta in tanycytes that were also detected by immunofluorescence with antibodies against GPHN or PSD95 (Supplementary Fig. 1k, l). In accordance with our observations using immunostaining of post-synaptic proteins above, FingR-labeled post-synaptoid puncta were apposed to pre-synaptic terminals (Fig. 1i).

To support the presence of glutamatergic synaptoids on tanycytes, we expressed the glutamate sensor iGluSnFR3 in tanycytes. When examining acute coronal brain slices with confocal microscopy^34^, the sensor was localized throughout the cell bodies and processes of tanycytes, as shown by fluorescence under basal conditions and after adding glutamate to the perfusate (Fig. 1j, Supplementary Fig. 3, and Supplementary Videos 1 and 2). However, following stimulation of neuronal network activity with a pulse of KCl (30 mM), glutamate concentrations increased briefly in individual small puncta that were mostly localized on tanycytic processes and rarely on tanycytic cell bodies, as shown by the fluorescence contour of tanycytes at baseline (Fig. 1j, k and Supplementary Videos 3–5, *n* = 8 mice). Tetrodotoxin (TTX), an inhibitor of voltage-gated sodium channels, reversibly attenuated the occurrence of KCl-triggered puncta along tanycyte contours (Fig. 1l and Supplementary Videos 6 and 7, *n* = 2 mice). Since tanycytes do not express voltage-gated sodium channels^22^, this inhibition of KCl effects by TTX indicates the involvement of neuronal action potentials and glutamate release at tanycytic synaptoids.

Overall, the data demonstrate that tanycytes carry a large number of synaptoids that resemble neuronal excitatory and inhibitory synapses. Consistent with this, we found that tanycytes receive glutamatergic inputs from afferent neurons.

### Synaptoid connectivity of neurons with tanycytes

**Fig. 2:**
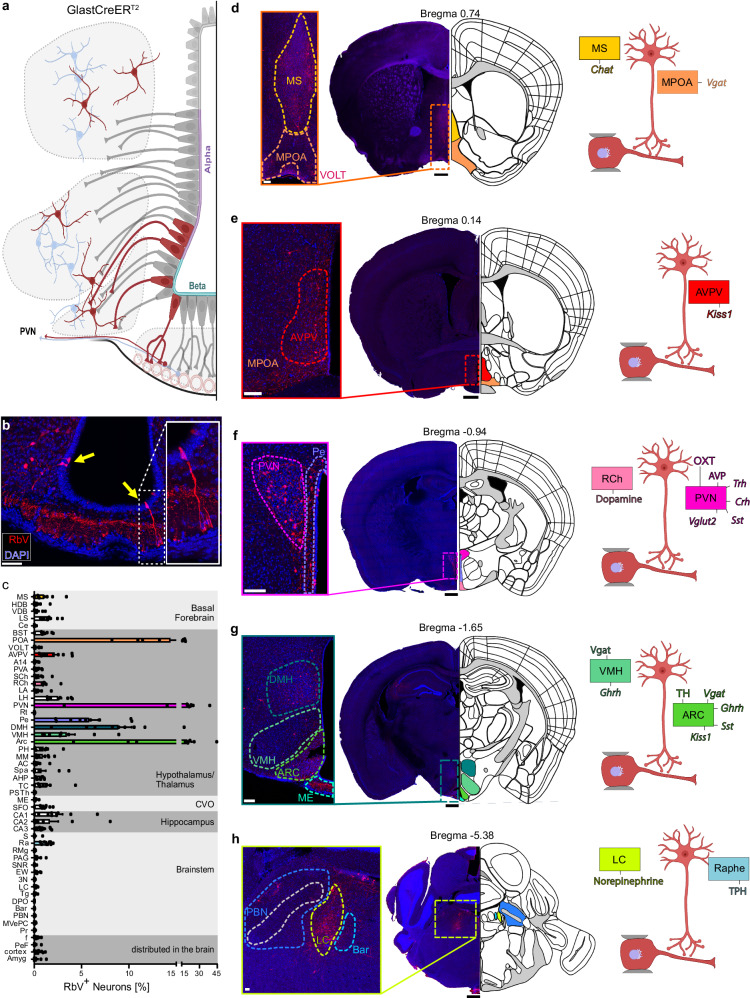
Retrograde tracing of neurons innervating α- and β-tanycytes. **a** Scheme of retrograde mono-synaptic rabies virus (RbV) tracing. In *GlastCreER*^*T2*^ mice, Cre-dependent expression of the RbV receptor TVA and G occurred in α- and β-tanycytes that served as starter cells. Created in BioRender. Schwaninger, M. (2026) https://BioRender.com/anffcqh. **b** Tanycytes expressed the RbV-encoded RFP (red, RbV, arrows) indicating transduction. Scale bar, 50 µm; magnified image, 25 µm. **c** Number of retrogradely traced neurons per mouse in different brain regions. Mean + SEM, *N* denotes the number of mice. **d–h** Representative overview images and brain diagrams showing distribution and identity of retrogradely traced neurons in different brain regions. Identity of RbV^+^ neurons were determined by IHC or in situ hybridization (for further information, see Supplementary Figs. 4 and 5). 3N oculomotor nucleus, A14 a14 dopaminergic cells, AC anterior commissural nucleus, AHP anterior hypothalamic area, posterior part, Amyg amygdala, ARC arcuate nucleus, AVPV anteroventral periventricular nucleus, Bar Barrington’s nucleus, BST bed nucleus stria terminalis, CA1-3 cornu ammonis 1-3, Ce central amygdaloid nucleus, CVO circumventricular organ, DMH dorsomedial hypothalamus, DPO dorsal periolivary region, DR dorsal raphe, EW Edinger–Westphal nucleus, f fornix, HBD horizontal diagonal band of Broca, LA lateroanterior hypothalamic nucleus, LC locus coeruleus, LH lateral hypothalamus, LS lateral septal nucleus, ME median eminence, MM medial mammillary nucleus, MPOA medial preoptic area, MS medial septal nucleus, MVePC medial vestibular nucleus, parvicellular part, PAG lateral periaqueductal gray, PBN parabrachial nucleus, Pe periventricular hypothalamic nucleus, PeF perifornical nucleus, PH posterior hypothalamic area, POA preoptic area, PPTg pedunculopontine tegmental nucleus, Pr prepositus nucleus, PSTh parasubthalamic nucleus, PVA paraventricular thalamic nucleus, PVN paraventricular hypothalamic nucleus, RbV rabies virus, Ra raphe nuclei except RMg, RMg raphe magnus nucleus, S subiculum, SCh suprachiasmatic nucleus, SFO subfornical organ, SNR substantia nigra, reticular part, Spa subparaventricular zone of the hypothalamus, TC tuber cinereum area, RCh retrochiasmatic area, Rt reticular thalamic nucleus, VDB vertical diagonal band of Broca, VMH ventromedial hypothalamus, VOLT vascular organ of the lamina terminalis. Black scale bars, 500 µm; white scale bars, 100 µm. The brain schemes are based on the Allen Mouse Brain Atlas and Allen Reference Atlas^109^. For information on the sex of mice, see Supplementary Data File 5.

To obtain a comprehensive picture of neuronal input, we used the rabies virus technology, which is established for retrograde tracing across neuron-neuron synapses and has also been used for characterizing close contacts between glial cells^25,35,36^. To express the rabies virus receptor TVA and glycoprotein G in tanycytes, we injected a Cre-dependent AAV expression vector into the lateral ventricle of *GlastCreER*^*T2*^ mice. This approach affords tanycyte-specific expression while sparing parenchymal astrocytes and neurons (Supplementary Fig. 5a)^17^. After injecting the Cre-dependent AAV1/2 vector *AAV-EF1a-flex-TVA-G* into the lateral ventricle, followed two weeks later by the pseudotyped, glycoprotein-deleted rabies virus (RbV) SADΔG-EGFP or SADΔG-RFP into the ARC, tanycytes expressed RbV-encoded *Gfp* or *Rfp* (RbV, Fig. 2a, b, *n *= 9 mice). In the brain parenchyma, numerous additional cells, all of which exhibited neuronal cell shapes, were RbV-positive, and no RbV-positive astrocytes were detected. We counted 3055 $\pm \,$ 921 RbV-positive neurons in female mice (mean ± SEM, *n* = 6 mice) and 2072 ± 897 RbV-positive neurons in male mice (*n* = 3 mice, Supplementary Fig. 4a). In contrast, when we injected only SADΔG-RFP into the mediobasal hypothalamus without expressing TVA and G, tanycytes were not RbV-positive, as expected, and in the whole brain, only 15 $\pm \,$9 cells (mean ± SEM, *n* = 2), all at the injection site, were RbV-positive, indicating that the labeling of neurons reflected their connection to tanycytes. The anatomical localization of retrogradely-labeled RbV-positive neurons was highly reproducible between mice and was restricted to specific nuclei (Fig. 2c). Most RbV-positive neurons were found in the basal forebrain and hypothalamic nuclei (Fig. 2d–g). Neurons in the paraventricular (PVN) (Fig. 2f) and the parabrachial nuclei (PBN) (Fig. 2h) were labeled, confirming previous reports that thyrotropin-releasing hormone (TRH)-expressing neurons of the PVN and glutamatergic neurons of the PBN innervate tanycytes^16,17^. The hypothalamic nuclei together contained 85.5 ± 2.0% (mean ± SEM, *n* = 9 mice) of all RbV-positive neurons. Some RbV-positive neurons were located in remarkably distant brain areas, such as the hippocampus and brainstem nuclei, including the dorsal raphe, Edinger–Westphal nucleus, and locus coeruleus (Fig. 2c, h), which are known to send projections to the mediobasal hypothalamus^37–41^. The distribution of retrogradely labeled neurons did not differ between the sexes (Supplementary Fig. 4a–d).

**Fig. 3:**
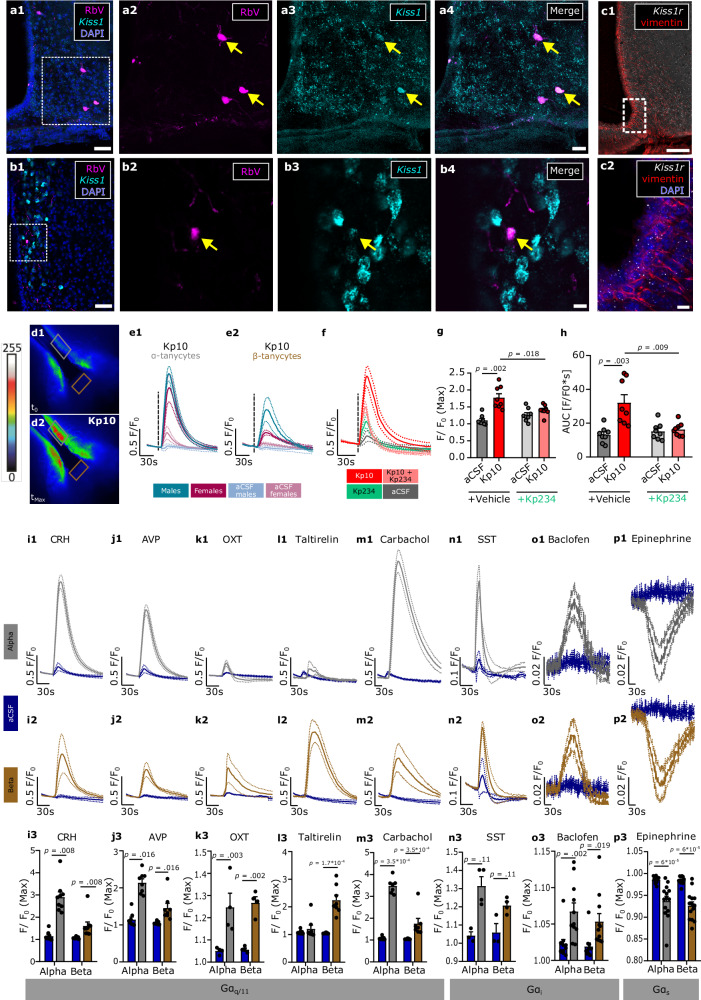
Kisspeptin neuron populations project on tanycytes. **a**, **b** Retrograde tracing experiments starting from tanycytes (Fig. 2) showed that RbV-positive neurons expressed *Kisspeptin1* (*Kiss1*) in the ARC (**a**) and the anteroventral periventricular nucleus (AVPV, **b**). *Kiss1* was detected by in situ hybridization. Scale bars overviews, 50 µm. Scale bars, a2–4, 10 µm; b2–b4, 25 µm. **c** Tanycytes expressed *Kiss1r*, as detected by in situ hybridization (grey). The tanycyte marker was labeled by immunostaining (red). Scale bar overview, 100 µm; magnified image, 10 µm. **d**, **e** The kisspeptin agonist Kp10 (1 mM) increased [Ca^2+^]_i_ more strongly in α- than in β-tanycytes, regardless of sex. Fluorescent heatmaps of a coronal slice before stimulation (*t*_0_, **d1**) and at maximum fluorescence (*t*_max_, **d2**) after stimulation are shown, as well as traces of the fluorescence in boxed areas relative to baseline (*F*/*F*_0_) (**e**), obtained from 5 male and 4 female mice with 2 slices per mouse. **f–h** The Kiss1R antagonist Kp234 inhibited the effect of Kp10 on tanycytic [Ca^2+^]_i_ in acute slices. Repeated-measures ANOVA with Sidak post-hoc test. **i–m** Tanycytic [Ca^2+^]_i_ in acute slices was increased by the following neurotransmitters (or their analogues) released from connected neurons: corticotropin-releasing hormone (CRH, 21 µM, **i**), vasopressin (AVP, 1 mM, **j**), oxytocin (OXT, 1 mM, **k**), taltirelin (33 µM, **l**), and carbachol (333 µM, **m**). **n–p** Tanycytic [cAMP]_i_ in acute slices was decreased by somatostatin (SST, 50 µM, **n**) and baclofen (1 mM, **o**), while stimulation with epinephrine (1 mM, **p**) increased [cAMP]_i_. Mean traces ± SEM (dotted lines) for α- (**i1–p1**) and β-tanycytes (**i2–p2**), as well as maximal F/F_0_ (**i3–p3**), are shown. For statistical analysis, Scheirer-Ray-Hare and Wilcoxon matched-pairs signed rank test (**i3**, **j3**) or two-sided Mann–Whitney *U* tests (**l3–n3**, **p3**) or RM-ANOVA and Sidak test (**k3**, **o3**) were used. Values (**e–p**) are means ± SEM, *N* denotes the number of mice. For information on the sex of mice and detailed information on the statistical analysis, see Supplementary Data File 5.

To characterize these retrogradely RbV-labeled neurons, we performed immunofluorescence labeling and RNAScope in situ hybridization. In line with the presence of both excitatory and inhibitory synaptoid terminals on tanycytes (Fig. 1f–l), RbV transduction of tanycytes retrogradely traced *Vglut2*-positive neurons in the PVN (Fig. 2f and Supplementary Fig. 5b), extending previous data that glutamatergic PBN neurons stimulate tanycytes^16,42^, as well as *Vgat*-positive GABAergic neurons in the medial preoptic area (MPOA), VMH, and ARC (Fig. 2d, g and Supplementary Fig. 5c, d). The latter is in line with reports that GABAergic neurons in the MPOA project to the mediobasal hypothalamus^43^. In the ARC, RbV-transduced neurons expressed growth hormone-releasing hormone (*Ghrh*, Fig. 2g and Supplementary Fig. 5e), kisspeptin *(Kiss1*, Figs. 2g and 3a), somatostatin (*Sst*, Fig. 2g and Supplementary Fig. 5f), and tyrosine hydroxylase (TH, Fig. 2g and Supplementary Fig. 5g). Indeed, immuno-EM confirmed that TH-positive pre-synapses terminated on tanycytes (Fig. 1a, b). While four of the major ARC neuronal types were connected with tanycytes, no agouti-related peptide (*Agrp*)- or pro-opiomelanocortin (*Pomc*)-expressing ARC neurons (Supplementary Fig. 6a, b) were retrogradely transduced, suggesting that retrograde tracing from tanycytes is highly specific and not simply a result of cellular proximity.

In the PVN, retrogradely-traced RbV-positive neurons expressed *Trh*, corticotropin-releasing hormone (*Crh)*, oxytocin (OXT), *Sst*, or arginine-vasopressin (AVP) in addition to *Vglut2* (Fig. 2f and Supplementary Figs. 5b and 6c–g), demonstrating that the major neuropeptidergic PVN neurons that release neurohormones in the ME and neurohypophysis are connected to tanycytes. We could not identify RbV-positive neurons that expressed TH in the PVN (Supplementary Fig. 6h), but did observe several in the retrochiasmatic area (Fig. 2f and Supplementary Fig. 6i), probably corresponding to dopaminergic neurons that are known to project to the mediobasal hypothalamus^44^. Moreover, RbV-positive neurons in the locus coeruleus expressed TH, corresponding to adrenergic neurons, while those in the raphe nucleus expressed tryptophan hydroxylase (TPH), a marker of serotoninergic neurons (Fig. 2h and Supplementary Fig. 6j, k), both neuronal populations known to project to the mediobasal hypothalamus^45,46^.

The anterior hypothalamus contains neuronal populations that are responsible for growth and reproduction. While GnRH-expressing neurons in the preoptic area (POA) were not RbV-labeled (Supplementary Fig. 6l), *Kiss1* neurons in the anteroventral periventricular nucleus (AVPV) of female and male mice were retrogradely labeled with RbV (Figs. 2e and 3b). In the medial septal nucleus (MS), we identified retrogradely-labeled neurons expressing choline O-acetyltransferase (*Chat*) (Fig. 2d and Supplementary Fig. 6m). This is in line with the observation that cholinergic neurons of the MS and the diagonal band of Broca (DBB) project to the ARC and ME, among other areas, and regulate metabolism^47,48^. In summary, tanycytes receive input from several neuronal populations releasing specific neurotransmitters and neuromodulators.

### Tanycytes respond to various neurotransmitters and neuromodulators

Given the synaptoid connections between various neuronal populations and tanycytes, we asked whether tanycytes could respond to the neurotransmitters and neuromodulators released by these neuronal populations. Tanycytes express AMPA glutamate receptors^16^ as well as G protein-coupled receptors for the primary neurotransmitters or neuromodulators of afferent neurons, as shown by scRNAseq analysis and confirmed by in situ hybridization for *Kiss1r*, *Chrm1*, *Trhr1*, *Sstr2*, *Oxtr*, and *Avpr1a* (Fig. 3c and Supplementary Fig. 7). Since tanycytes do not show active membrane properties upon depolarization and apparently signal through second messengers^16,49,50^, we investigated the functional responsivity of tanycytes by measuring intracellular Ca^2+^ ([Ca^2+^]_i_) or cAMP ([cAMP]_i_) levels (Supplementary Fig. 8c, l), depending on whether the receptors for specific neurotransmitters are known to be coupled to Gα_q/11_ or Gα_s_/Gα_i_ proteins, respectively. For this, we transduced tanycytes with AAV vectors for the Ca^2+^ sensor GCaMP6s or the cAMP sensor Flamindo2 and stimulated them in acute brain slices ex vivo. An increase in [Ca^2+^]_i_ enhances the fluorescence of GCaMP6s, while an increase in [cAMP]_i_ decreases the fluorescence of Flamindo2^51,52^. Intriguingly, tanycytes responded to numerous neurotransmitters and neuromodulators (or their analogues) released from retrogradely-labeled neurons: the Kiss1 analogue Kp10, the TRH analogue taltirelin, OXT, AVP, CRH, and the cholinergic receptor agonist carbachol elevated [Ca^2+^]_i_ in tanycytes in accordance with their known intracellular signaling pathways (Fig. 3d–m). Since the kisspeptin system is sexually dimorphic^53^, we tested whether brain slices from male and female mice would react differently to stimulation by Kp10. While Kp10 activated [Ca^2+^]_i_ in tanycytes, with a more pronounced effect in α- than in β-tanycytes, we did not detect a significant difference between males and females (Fig. 3d, e). To verify that Kp10 activated [Ca^2+^]_i_ in tanycytes through the Kiss1 receptor (Kiss1R), we used the specific receptor antagonist kisspeptin-234 (Kp234)^54^. Kp234 significantly inhibited the effect of Kp10 on [Ca^2+^]_i_ in tanycytes (Fig. 3f–h), demonstrating that Kiss1R mediates the effect of Kp10. Further experiments confirmed that the effect of carbachol was concentration-dependent and mediated by the cholinergic M1 receptor CHRM1 (Supplementary Fig. 8d–g)^55^. Epinephrine increased [cAMP]_i_, while the β-adrenoreceptor antagonist propranolol decreased it. Similarly, the GABA_B_ receptor agonist baclofen, the α_2_-adrenoreceptor agonist clonidine, SST, dopamine, and the dopamine receptor 2 agonist quinpirole reduced [cAMP]_i_ in tanycytes (Fig. 3n–p and Supplementary Fig. 8m–u). Stimulation in isolated primary cultured tanycytes confirmed the effect and provided evidence that these neurotransmitters or their analogues directly act on tanycytes, independent of other cell types (Supplementary Fig. 8a, b, v).

Interestingly, carbachol had a stronger effect on [Ca^2+^]_i_ in α-tanycytes (Fig. 3m), while taltirelin increased [Ca^2+^]_i_ mainly in β2-tanycytes (Fig. 3l). The differential response of tanycytes to taltirelin could be explained by the expression of *Trhr1*, which mediates the response to taltirelin^17^; mainly in β-tanycytes but not in α-tanycytes (Supplementary Fig. 7a, c). In contrast, *Chrm1*, which mediated the effect of carbachol on [Ca^2+^]_i_, was expressed in all tanycytes at a low level (Supplementary Fig. 7a, b). However, since the M1 and TRHR1 receptors signal through the same Gα_q/11_—phospholipase C—IP3 pathway (Supplementary Fig. 8h–k)^17^, the mechanism underlying the predominance of α-tanycytes in the response to carbachol is still unclear.

Overall, the data demonstrate that tanycytes respond in a differential and subpopulation-specific manner to classic neurotransmitters and neuromodulators released by afferent neurons.

### Targeting tanycytic subpopulations

**Fig. 4:**
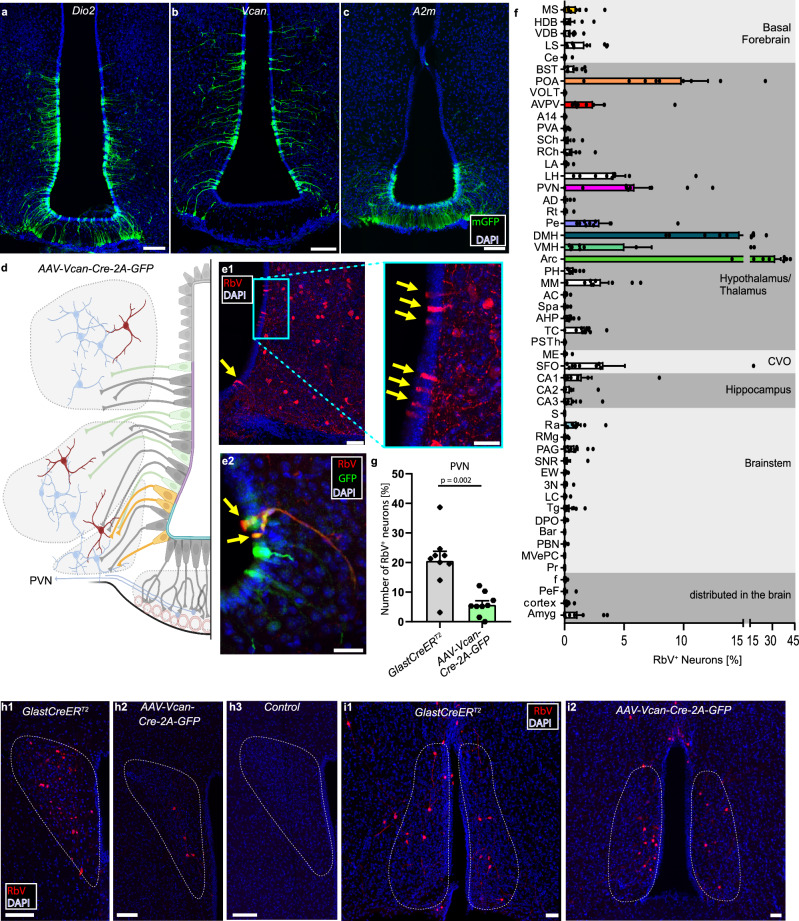
Transcriptional targeting of α- and β1-tanycytes enables specific retrograde tracing. **a–c** *Dio2*, *Vcan*, and *A2m* promoters directed gene expression into different tanycyte subpopulations. AAV vectors, in which promoters control Cre expression, were injected in the lateral ventricle of *mT/mG* reporter mice. mGFP (green) expression in representative sections is shown. The number of replicates are given in Supplementary Data File 5. Scale bar, 100 µm. **d** Scheme of retrograde mono-synaptic rabies virus (RbV) tracing. Due to the *AAV-Vcan-Cre-2A-GFP* vector, Cre-dependent expression of the RbV receptor TVA and G was directed to α- and dorsal β1-tanycytes that served as starter cells. Created in BioRender. Schwaninger, M. (2026) https://BioRender.com/anffcqh. **e** Tanycyte transduced with both *AAV-Vcan-Cre-2A-GFP* and RbV expressed GFP (green) and the RbV-encoded RFP (red, arrows). Scale bar overview **e1**, 50 µm; magnified image and **e2**, 25 µm. **f** Number of retrogradely traced neurons in different brain regions. Mean + SEM. **g** The retrograde tracing from *Vcan*-targeted tanycytes showed significantly fewer RbV-positive neurons in the paraventricular nucleus (PVN) than when tracing started from *Glast*-targeted tanycytes (same as in Fig. 2). RbV neuron counts per nucleus are expressed as percentage of total RbV neuron counts. Values are means + SEM, *N* denotes the number of mice. Two-sided Mann–Whitney *U* test. Detailed information on the test statistics is provided in Supplementary Data File 5. **h** In the PVN, numerous RbV-positive neurons (red) were found after tracing from *Glast*-targeted tanycytes (**h1**), but only few neurons after tracing from *Vcan*-targeted tanycytes (**h2**). In control conditions, no neurons were labeled in the PVN (**h3**). Scale bars, 100 µm. **i** In the anteroventral periventricular nucleus (AVPV), similar numbers of neurons were retrogradely traced when starting from *Glast*-targeted α- and β-tanycytes (**i1**) or *Vcan*-targeted α- and dorsal β1-tanycytes (**i2**). Scale bars, 50 µm. For information on the sex of mice, see Supplementary Data File 5.

The observation that specific neurons connect to tanycytes through synaptoid junctions raises the question of whether neuronal innervation differs between tanycytic subpopulations. Previous work on tanycytic physiology relied on genetic (e.g., *GlastCreER*^*T2*^ mice) or viral tools (e.g., *AAV-Dio2-Cre*)^56^ but could not distinguish between tanycytic subpopulations. To target tanycytic subpopulations, we searched for genes that are selectively expressed in these subpopulations. Based on the Tanybase scRNAseq and in situ hybridization (Allen Brain atlas, https://portal.brain-map.org/) databases, we picked 20 genes that were expressed in tanycytes with varying specificity across tanycytic subpopulations (Supplementary Fig. 9a). We used the promoter region of these genes to drive Cre expression from AAV1/2 vectors (Supplementary Data File 1 for more information regarding the promoter sequences). After injection into Cre reporter mice, all vectors mediated recombination in tanycytes, as shown by the reporter in tanycytic cell bodies and processes encircling vessels (Supplementary Fig. 9b). Only *Adm*, *Crym, Col25a1*, and *Frzb* promoters targeted non-tanycytic cells in the parenchyma in addition to tanycytes (Supplementary Fig. 9b). The distribution of recombinant tanycytes along the ventricular wall differed between promoters, and for some promoters, reflected endogenous gene expression. Thus, consistent with the expression of the endogenous genes^22^, the *Sprr1a* promoter directed recombination to β1- and isolated α1-tanycytes, the *Vcan* promoter mainly to α- and dorsal β1-tanycytes, the *A2m* promoter to β1 + β2-tanycytes, and the *Dio2* promoter to all tanycytic subpopulations (Fig. 4a–c and Supplementary Fig. 9b). Overall, these data confirm tanycytic diversity and suggest that it has a transcriptional basis. As these vectors do not target the parenchyma when injected into the lateral ventricle (Supplementary Fig. 10), they offer tools to study the functional implications of tanycytic diversity.

### Tanycytic subpopulations are specialized in terms of neuronal input

Taking advantage of the selective recombination made possible by the AAV vectors described above, we repeated the RbV tracing experiments but limited the starter cells to α- and dorsal β1-tanycytes that are targeted by the selective *AAV-Vcan-Cre* vector (*Vcan*-targeted tanycytes). After injecting *AAV-Vcan-Cre-2A-GFP* plus *AAV-EF1a-flex-TVA-G* into the lateral ventricle and SADΔG-RFP into the ARC, we found GFP/RFP double-positive starter α- and dorsal β1-tanycytes as well as several RFP-labeled neurons (RbV, Fig. 4d, e, *n* = 9 mice). When targeting tanycytes with the *Vcan* promoter, retrogradely-labeled neurons were mostly located in the same nuclei (Fig. 4f) as in previous experiments in which all tanycytes served as starter cells due to the *GlastCreER*^*T2*^ mouse line (Fig. 2). However, only 1320 ± 683 (mean ± SEM) neurons were retrogradely labeled from *Vcan*-targeted tanycytes in female mice (*n* = 5 mice) and 2449 ± 937 neurons in male mice (*n* = 4, Supplementary Fig. 4e). Importantly, the reduction affected specific nuclei, mainly the PVN (Fig. 4g, h), while others, such as the AVPV, still contained numerous labeled neurons (Fig. 4i). Similar to the experiments in which retrograde labeling started from *Glast*-targeted tanycytes, no difference between the sexes was detected (Supplementary Fig. 4e–h). Overall, the comparison of retrogradely-traced neurons starting either from *Vcan*-targeted tanycytes or from all tanycytes suggests that -tanycyte subpopulations receive different inputs, with PVN neurons preferentially innervating ventral β1 and β2-tanycytes.

### Kiss1 neuron–tanycyte connections differentially regulate the hypothalamic-pituitary-gonadal axis and estrous cycle

To investigate the functional significance of these differential neuronal inputs to tanycytes, we focused on the connection with Kiss1 neurons identified by retrograde tracing experiments. The Kiss1 neuronal populations of the ARC and AVPV are key regulators of sexual function and the HPG axis^57^. Kiss1 directly stimulates the secretion of GnRH^58,59^, which travels via the pituitary portal vessels to the anterior pituitary and triggers the release of gonadotropins. Interestingly, Kiss1 regulates both the frequency and amplitude of gonadotropin pulses. However, increasing evidence suggests that Kiss1 neurons mediate some neuroendocrine functions by acting on cells other than GnRH neurons^60,61^, including possibly glial cells^62^. Because (1) tanycytes are connected to Kiss1 neuronal populations in both the AVPV and ARC (Fig. 3a, b), (2) tanycytes express the Kiss1 receptor *Kiss1r* (Fig. 3c), (3) Kiss1 increases [Ca^2+^]_i_ in tanycytes (Fig. 3d, e), (4) Kiss1 is known to stimulate GnRH release from explants of the mediobasal hypothalamus^58,59,63^, and (5) the retraction of tanycytic endfeet that ensheathe GnRH neuronal terminals is known to control GnRH release^18,21^, we hypothesized that tanycytes could play a role in controlling GnRH release through the input they receive from Kiss1 neurons. Selective labeling of Kiss1 neurons in *Kiss-Cre*; *mT/mG* mice confirmed that many Kiss1 neurites occur in close proximity to tanycytic processes in the ME and ARC (Supplementary Fig. 11a, b)^63,64^. Using tissue clearing and light sheet microscopy, we were able to track Kiss1 neurites from the AVPV to the mediobasal hypothalamus (Supplementary Video 8). Selective labeling of Kiss1 neurons by injecting the reporter vector *AAV-flex-mCherry* into the AVPV of *Kiss-Cre* mice confirmed that Kiss1 neuronal projections from AVPV are abundant just below the tanycytic cell body layer in the ME, suggesting that they contact the proximal processes of these tanycytes (Supplementary Fig. 11c, d). Accordingly, the neurites of AVPV Kiss1 neurons were closely apposed to NLGN2-positive post-synaptoid puncta in tanycytes (Supplementary Fig. 11e, f). Along similar lines, a previous paper reported that the ARC Kiss1 neurons expressing neurokinin B (NKB) form direct morphological contacts with tanycytes at the ultrastructural level^65^. Overall, these data provide strong evidence that Kiss1 neurons project to the mediobasal hypothalamus and are connected to tanycytes via synaptoid junctions.

**Fig. 5:**
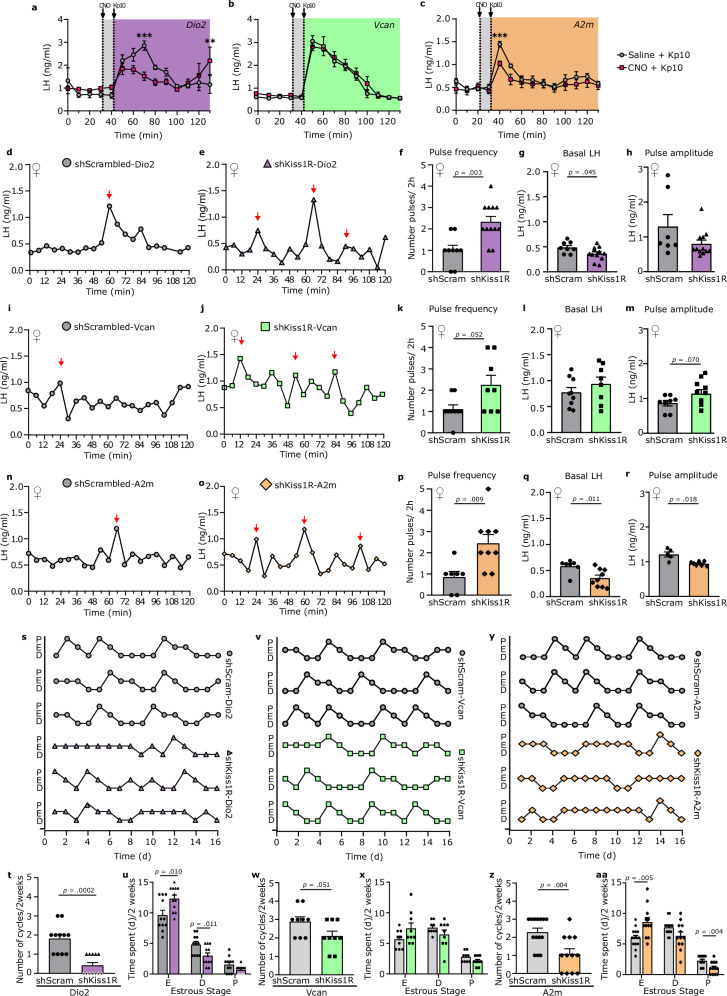
Tanycyte subpopulations mediate a differential effect of kisspeptin on LH secretion and the estrous cycle. **a–c** Stimulation of LH secretion by the kisspeptin agonist Kp10 (1 nmol) was reduced when *Dio2*-targeted α- and β-tanycytes or *A2m*-targeted β-tanycytes were inhibited, while inhibition of *Vcan*-targeted α- and dorsal β1-tanycytes had no effect. Female *hM4Di* mice received either *AAV*-*Dio2-Cre* (α + β-tanycytes, **a**, *p* = 0.003 at *t* = 70 min, RM two-way ANOVA followed by Sidak’s tests), *AAV-Vcan-Cre-2A-GFP* (α- + dorsal β_1_-tanycytes, **b**), or *AAV-A2m-Cre-2A-GFP* (β-tanycytes, **c**, *p* = 0.001 at *t *= 40 min). Ten min before Kp10, the mice were treated with clozapine-N-oxide (CNO, 1 mg/kg body weight) to activate the DREADD inhibitor hM4Di, and one week later they were treated with saline, or vice versa. Please note that the *y*-axis range in (**a** and **b**) differs from that in panel **c** due to experimental variations. **d**, **e** Representative profile of LH blood concentrations in female mice injected with *AAV-flex-shScramble-GFP* (**d**) or *AAV*-*flex-shKiss1r-GFP* (**e**) together with *AAV*-*Dio2-Cre*. Time points considered as a LH pulse were labeled with red arrows. **f–h** LH pulse frequency (**f**), basal plasma concentrations (**g**), and pulse amplitude (**h**) in female mice in which *Kiss1r* was knocked down in *Dio2*-targeted α- and β-tanycytes. **f–h** were analyzed by a two-sided Mann–Whitney *U* test. **i**, **j** Representative profile of LH blood concentrations in female mice injected with *AAV-flex-shScramble-GFP* (**i**) or *AAV-flex-Kiss1r-GFP* (**j**) together with *AAV*-*Vcan-Cre-2A-GFP*. **k–m** LH pulse frequency (**k**), basal plasma concentrations (**l**), and pulse amplitude (**m**) in female mice in which *Kiss1r* was knocked down in *Vcan*-targeted α- and dorsal β1-tanycytes. **k** and **m** were analyzed by a two-sided Mann–Whitney *U* test, **l** by unpaired *T*-test. **n**, **o** Representative profile of LH blood concentrations in female mice injected with *AAV-flex-shScramble-GFP* (**n**) or *AAV*-*flex-shKiss1r-GFP* (**o**) together with *AAV*-*A2m-Cre-2A-GFP*. **p–r** LH pulse frequency (**p**), basal plasma concentrations (**q**), and pulse amplitude (**r**) in female mice in which *Kiss1r* was knocked down in *A2m*-targeted β-tanycytes. **p** and **q** were analyzed by unpaired a two-sided *T*-test, **r** by a two-sided Welch *t*-test. **s–aa** Estrous cyclicity in representative female mice which received *AAV-flex-shScramble-GFP* or *AAV-shKiss1r-GFP* with either *AAV*-*Dio2*-*Cre* (**s**), *AAV-Vcan-Cre-2A-GFP* (**v**), or *AAV-A2m-Cre-2A-GFP* (**y**). Number of complete estrous cycles and days of permanency in estrus, diestrus, or proestrus after *Kiss1r* knockdown in *Dio2*-targeted tanycytes (**t**, **u**), in *Vcan*-targeted tanycytes (**w**, **x**), or *A2m*-targeted tanycytes (**z, aa**). **t** and **z** were analyzed by Mann–Whitney *U*, while (**w**) was analyzed by unpaired a two-sided *T*-test. **u**, **x**, and **aa** were analyzed by multiple Mann–Whitney *U*, being Holm Sidak corrected. Mean + SEM, *N* denote the number of mice. Detailed information on the test statistics and number of animals is provided in Supplementary Data File 5.

Systemically administered Kiss1 enhances LH release^66^, a surrogate for GnRH release, into the circulation, presumably by activating the same pathways as Kiss1 neurons. To test whether and which tanycytes are involved in the effect of Kiss1 in vivo, we used the inhibitory DREADD approach (hM4Di). We expressed *hM4Di* in 3 partially overlapping subsets of tanycytes—*Dio2*-targeted α + β-tanycytes, *Vcan*-targeted α- + dorsal β1-tanycytes, and *A2m*-targeted β1 + β2-tanycytes—by injecting *AAV-Dio2-Cre* (tanycytes^*Dio2::hM4Di*^ mice), *AAV-Vcan-Cre-2A-GFP* (tanycytes^*Vcan::hM4Di*^ mice), or *AAV-A2m-Cre-2A-GFP* (tanycytes^*A2m::hM4Di*^ mice) into the lateral ventricle of *hM4Di* mice (Supplementary Fig. 12a). While, as expected, the Kiss1R agonist Kp10 (1 mol/25 g body weight, intraperitoneally) stimulated the release of GnRH/LH in diestrus female mice, pre-treatment with the DREADD activator clozapine-N-oxide (CNO, 1 mg/kg body weight) blunted Kp10-induced LH release in both tanycytes^*Dio2::hM4Di*^ and tanycytes^*A2m::hM4Di*^ mice in which the population of ventral β1 tanycytes at the frontier between the ME and ARC, known to ensheathe GnRH neuronal terminals^18^, would have been targeted, but not in tanycytes^*Vcan::hM4Di*^ mice, in which the ventral β1-tanycytes controlling GnRH terminal access to the circulation are spared (Fig. 5a–c and Supplementary Fig. 12b). These findings indicate that tanycytes play an active role in the Kiss1-mediated regulation of the HPG axis. In particular, the data suggest that ventral β1-tanycytes of the ME, which are known to ensheathe the terminals of GnRH neurons and thus regulate GnRH secretion^18^, and which are comprehensively transduced by both *Dio2* and *A2m* targeting AAVs, mediate the Kiss1 effect on the amplitude of GnRH release.

The effect of Kiss1 is mediated by Kiss1R in vivo^66^. Tanycytes expressed *Kiss1r* (Fig. 3c; Supplementary Fig. 7a). FACS isolation of fluorescently labeled tanycytes and RT-PCR demonstrated that tanycytic *Kiss1r* expression increased from diestrus to proestrus and markedly dropped in female mice after ovariectomy (Supplementary Fig. 13a–c). To silence *Kiss1r* selectively in either α + β-, α + dorsal β1-, or β1 + β2-tanycytes, we co-injected the Cre-dependent *AAV-flex-shKiss1r-GFP* along with *AAV-Dio2-Cre, AAV-Vcan-Cre-2A-GFP*, or *AAV-A2m-Cre-2A-GFP*, respectively, into the lateral ventricle of mice. FACS isolation of tanycytes confirmed that *Kiss1r* expression was efficiently knocked-down in the three populations of shRNA-expressing tanycytes (Supplementary Fig. 13d–g). Interestingly, the knockdown of *Kiss1r* in tanycyte subpopulations of female mice differentially affected LH pulsatility at estrus. While all three interventions increased the frequency of LH pulses, suggesting that α- or dorsal β1-tanycytes exert a Kiss1-mediated slowdown of GnRH pulsatility, only the knockdown of *Kiss1r* in tanycyte^*Dio2::shKiss1R*^ mice and in tanycyte^*A2m::shKiss1R*^ mice (in which both β2 tanycytes of the ME and ventral β1 tanycytes that ensheathe GnRH neurons are targeted) lowered basal LH levels (Fig. 5d–q and Supplementary Fig. 13h–p). In addition, LH pulse amplitudes were significantly lower when *Kiss1r* was knocked-down in *A2m*-targeted β1 + β2-tanycytes but not in *Vcan*-targeted α- + dorsal β1-tanycytes (Fig. 5m, r). Similar results were obtained in males in which *Kiss1r* was knocked-down in tanycytes^*Dio2::shKiss1R*^ and tanycytes^*Vcan::shKiss1R*^ mice (Supplementary Fig. 13v–z). Notably, the knockdown of *Kiss1r* in *A2m*-targeted β-tanycytes of male mice increased rather than decreased basal LH levels as in female mice (Supplementary Fig. 13aa–ae), probably reflecting sexual dimorphism in the Kiss1 input from AVPV neurons^53^. Taken together, these findings indicate that the Kiss1-mediated neuronal input to α- or dorsal β1-tanycytes primarily regulates LH pulse frequency, especially in males, whereas input to ventral β1- and β2-tanycytes modulates both basal LH levels and the pulsatile LH release pattern in both sexes. Of note, while selectively blunting *Kiss1r* expression in *Vcan*-targeted α- + dorsal β1-tanycytes only had a mild effect on estrous cyclicity (Fig. 5v–x), *Kiss1r* knockdown in *Dio2*-targeted α + β-tanycytes or in *A2m*-targeted β-tanycytes blunted the estrous cycle and prolonged the time in estrus (Fig. 5s–u, y–aa) when compared to sham-treated mice that received the *AAV-flex-shScramble-GFP* virus, again suggesting a differential effect of β-tanycytes on GnRH release characteristics.

As a whole, our findings demonstrate that specific neuronal populations form functional synaptoid contacts with tanycytes, thereby integrating these glial cells into the neural network. This neuron-to-tanycyte communication may enable neurons to modulate effects traditionally attributed to neuron-to-neuron synaptic transmission, with significant implications for physiological functions.

## Discussion

As the basis for brain function, neurons connect to each other via synapses to form networks, while glial cells are generally regarded as essential for maintaining neuronal activity, including synapse stability and function, rather than as direct recipients of synaptic transmission. The data presented here indicate that tanycytes are an exception: unlike other glial cell types, they form direct structural and functional connections with neurons, suggesting a unique mode of integration into neural circuits.

Tanycytes have features of radial glial cells that persist in the mature brain^11^, one being the ability to form axoglial synaptoid junctions^67^, previously identified by EM^12–15,68,69^. While the term synaptoids was originally coined to describe “contacts which morphologically resemble synapses elsewhere in the CNS”^70^, we considered it likely that neuron-tanycyte synaptoid contacts also functioned in a similar way to synapses between neurons. Supporting this notion, we show here using imaging with high-resolution immunofluorescence microscopy that neuron-tanycyte synaptoids have a similar molecular composition to that of synapses, containing typical pre- and post-synaptic proteins known from neuron-neuron synapses and exhibiting a similar size and shape to the latter. In addition, synaptoids functionally connect neurons to tanycytes, as shown by the RbV-based retrograde tracing experiments.

The striking number of synaptoids per tanycyte, which we estimated at a few hundred to several thousand, being higher in α- than in β-tanycytes, is on a par with the number of synapses estimated for cortical neurons. Previous EM studies have yielded lower numbers of synaptoids per tanycyte, but may have underestimated the ramified morphology of tanycytic processes^13^. In addition, while only a few neuronal types, including TRH neurons in the PVN and glutamatergic neurons in the PBN^16,17^, were known to innervate tanycytes until now, the large diversity of innervating neurons that we have identified and the multiple neurotransmitters for which tanycytes express receptors and to which they respond are unprecedented. These connections seem to be specific, and not just determined by the proximity of neuronal terminals to tanycytes, since we found no evidence for the innervation of tanycytes by GnRH neurons of the POA or by POMC and AgRP neurons of the ARC, all of which send projections that closely intermingle with tanycytic processes in the mediobasal hypothalamus. However, it is possible that the apparent lack of retrograde labeling of a specific neuronal population is due to technical issues, as rabies virus transduction can down-regulate gene transcription, complicating the identification of retrogradely traced cells^71^.

The neurons that innervate tanycytes use the classical neurotransmitters glutamate, GABA, and acetylcholine, as well as neuromodulators. Traditionally, neuromodulators were thought to reach their receptors via volume transmission^72^, which may occur when neuromodulators spill out of the synaptoid cleft and diffuse to extrasynaptoid receptors or neighboring tanycytes. Signal spread may be increased by tanycyte coupling through gap junctions^73^. However, recent imaging studies have cast doubt on the idea that neuromodulators spread farther than neurotransmitters do^74,75^. Indeed, neuropeptide receptors are localized at least partially in neuronal postsynapses through interaction with postsynaptic scaffolding proteins^76,77^. The notion of a wired transmission and a “tanycytic connectome”, whereby specific neurons are connected with specific tanycytes, is supported by observations that different subpopulations of tanycytes receive inputs from different neurons and respond to distinct stimuli. For instance, α-tanycytes appear to respond primarily to cholinergic stimulation, β-tanycytes receive innervation from TRH neurons in the PVN and respond to a TRH agonist, while other stimuli, such as Kiss1, stimulate both α- and β-tanycytes.

Tanybase and other scRNAseq studies have also shown that tanycytes consist of multiple subpopulations^22,23,27^. In line with the concept that tanycytic heterogeneity is under transcriptional control, short promoter sequences of some genes were sufficient to direct gene expression to different tanycyte subpopulations. Taking advantage of this transcriptional specificity, we found that, while Kiss1R activation stimulates both α- and β-tanycytes, Kiss1R signaling in α- and dorsal β1-tanycytes selectively regulates LH pulse frequency, especially in males. Indeed, these tanycytes lie close to Kiss1 neurons in the ARC, which fire in a synchronized manner, releasing Kiss1^64^ and thereby driving the pulsatile release of GnRH and LH, essential for sexual function^78–80^. In contrast, Kiss1R signaling in β-tanycytes, including the ventral β1 population that ensheathes GnRH neuronal terminals in the ME, shapes the full pulsatile LH profile, modulating pulse frequency, amplitude, and basal LH release in both sexes, but the direction of the change in basal LH levels is inverted in males versus females. β-tanycytes of the ME receive preferential input from Kiss1 neurons in the AVPV, a neuronal population that controls LH release and its surge during proestrus^78,81^. Morphological changes in ventral β1-tanycytes, namely the retraction of tanycytic endfeet, are known to control the access of GnRH terminals, which they ensheathe, to the portal vessels underlying the ME^18,21,82^. Since Kiss1 via Kiss1R stimulates Gα_q/11_-Ca^2+^ signaling, which regulates the morphology of tanycytic endfeet^17^, it is easy to imagine how Kiss1 acting on specific subpopulations of β-tanycytes could influence GnRH and subsequently LH release. How α + β1-tanycytes influence LH pulse frequency is less clear, but this may involve the synchronization of Kiss1 neurons in the ARC that accompanies pulsatile LH release^83^. The projection of Kiss1 neurons of the ARC to tanycytes, which at the level of the ARC act as a syncytium due to their gap junctions, and their hypothetical feedback to Kiss1 neurons, as has been shown for other ARC neurons^20,21,73,84^, may contribute to this synchronization.

The differential effects of α + dorsal β1- and ventral β1 + β2-tanycytes on the HPG axis support the concept that distinct neuronal populations are connected to tanycytes in a specific manner, and that tanycytes function as integral components of neuroendocrine networks. Among the hypothalamic neurons that project to tanycytes are CRH and vasopressin neurons of the PVN, the master regulators of the hypothalamic-pituitary-adrenal (HPA) axis. These neuromodulators stimulated a Ca^2+^ response in tanycytes that may underlie changes in tanycytic endfoot size after stress and the regulation of the HPA axis by tanycytes^85,86^. In addition, innervation by various neurons of the hippocampus, brainstem, and basal forebrain could provide input about memory, emotion, autonomic control, and other processes that are required to drive endocrine regulation and homeostasis^87,88^. Hippocampal neuron-to-tanycyte connections are particularly intriguing as they could conceivably participate in modulating the tanycyte-mediated CSF‑to‑blood efflux of pathogenic proteins (recently shown for Tau), which first accumulate in the hippocampus when clearance mechanisms fail and thereby contribute to neuropathology^89^. Thus, tanycytes could function as a central hub that coordinates the actions of a variety of neuroendocrine, “metacrine” (a term first coined in ref. ^89^ to refer to the 2-way, functionally relevant secretion of molecules not produced by tanycytes themselves) and metabolic networks by receiving information from various populations via synaptoid connections.

## Methods

### Animals

Genetically modified mouse lines were established on a C57BL/6 background. The sex of mice in individual experiments are given in the Supplementary Data File 5. Adult mice (8–26 weeks of age) were maintained at a constant temperature (21–22 °C) on a 12-h light/dark cycle with *ad libitum* access to standard autoclaved laboratory chow (3.3 kcal/g; Altromin 1314 M fortified, Hanover, Germany) and water in individually ventilated cages. The *GlastCreER*^*T2*^ (Slc1a3^tm1(cre/ERT2)Mgoe^), *mT/mG* (Gt(ROSA)26Sor^tm4(ACTB-tdTomato,-EGFP)Luo^/J, RRID:IMSR_JAX:007676), *Ai14* (Gt(ROSA)26Sor^tm14(CAG-tdTomato)Hze^, RRID:IMSR_JAX:007914), *Kiss1-Cre* (Kiss1^tm1.1(cre/EGFP)Stei^/J, RRID:IMSR_JAX:017701), *Gα*_*11*_^*−/−*^*;Gα*_*q*_^*fl/fl*^ (Gna11^tm1Soff^; Gnaq^tm2Soff^), and *hM4Di* (Gt(ROSA)26Sor^tm1(CAG-CHRM4*,-mCitrine)Ute^/J, RRID:IMSR_JAX:026219*)* have previously been described^28,90–94^. For *Gα*_*q/11*_ deletion in glial cells, we used *GlastCreER*^*T2*^*;Gα*_*11*_^*−/−*^*;Gα*_*q*_^*fl/fl*^ mice (*Gα*_*q/11*_^*gliaKO*^) and as controls Cre-negative *Gα*_*11*_^*+/−*^*;Gα*_*q*_^*fl/fl*^ littermates, both treated with tamoxifen^17^. To induce recombination, we treated mice harboring the *GlastCreER*^*T2*^ allele with tamoxifen (1 mg, dissolved in 90% miglyol 812/10% ethanol per 20 g body weight, i.p.) twice per day on five consecutive days.

Wild-type C57BL/6N mice and Sprague Dawley rats were obtained from Charles River.

### Study approval

All animal experiments were performed with the approval of the local animal ethics committees (Ministerium für Landwirtschaft, ländliche Räume, Europa und Verbraucherschutz, 1–1/20, 8–2/20, 34–5/21, 60–7/18, 17–3/22; 74–11/24; 14–2/25; Institutional Ethics Committees for the Care and Use of Experimental Animals of the University of Lille and the French Ministry of National Education, Higher Education and Research, APAFIS#29172-2020 121811279767 v5) and under the guidelines defined by the European Union Council Directive of September 22, 2010 (2010/63/EU).

### AAV production

Promoter sequences were amplified from mouse genomic DNA by PCR using primers containing restriction sites for *MluI* at the 5’-end and either *NheI*, *XbaI*, or *AgeI* at the 3’-end (Supplementary Data File 1). The promoter sequences were first inserted into the pMini 2.0 construct (NEB PCR Cloning Kit) and then transferred into the plasmid *pAAV-Dio2-Cre-2A-GFP*, substituting the *Dio2* promoter^17^ using the restriction sites for *MluI* and either *NheI*, *XbaI*, or *AgeI* depending on the promoter sequence. *Cers6* was synthesized by Vectorbuilder. For transduction of tanycytes, AAVs were produced with a mosaic capsid of serotypes 1 and 2 in HEK293 cells and purified by an iodixanol gradient as previously described^17^. For labeling of Cre-expressing neurons, we amplified the coding sequence of mGFP from *mT/mG* mice^28^ to generate *pAAV-CMV-flex-mGFP* that was packaged in AAV9 vectors.

### Virus injections

Mice were anesthetized with ketamine (65 µg/g body weight, i.p.) and xylazine (14 µg/g, i.p.) or with isoflurane (induction, 3%; maintenance, 1%), and fixed in a stereotaxic frame (Kopf Instruments) as previously described in ref. ^17^. For transduction of tanycytes, AAVs were injected into the lateral ventricle. Two weeks after vector injection, mice were re-anesthetized and transcardially perfused first with Ringer’s solution containing heparin (10 IU/ml) and then with 4% paraformaldehyde (PFA) in PBS. Viral doses are listed in Supplementary Data File 4. Brains were postfixed in 4% PFA for 6–7 h at 4 °C.

For retrograde tracing from *Glast*-targeted tanycytes, we injected *AAV-EF1a-flex-TVA-G*^95^ into the lateral ventricle of *GlastCreER*^*T2*^ mice. From the next day, tamoxifen was injected i.p. twice a day for five days to induce recombination in the tanycytes. Two to three weeks after AAV injection, pseudotyped, glycoprotein-deleted rabies virus (SADΔG-EGFP or SADΔG-RFP)^95^ was injected bilaterally into the ARC. After 7 days, mice were perfused as described above.

For retrograde tracing from *Vcan*-targeted tanycytes, *AAV-Vcan-Cre-2A-GFP* and *AAV-EF1a-flex-TVA-G* were injected into the lateral ventricle, and after two to three weeks, SADΔG-RFP was injected into the ARC.

### Primary tanycyte culture

Primary tanycytes were isolated from P10 rats by dissecting the wall of the 3rd ventricle of the mediobasal hypothalamus and ME as described previously^96,97^. Briefly, after washing the brains three times in ice-cold PBS, the mediobasal hypothalamus and ME were dissected under a binocular microscope. The tissue was collected in growth medium (DMEM high-glucose medium containing 10% donor bovine serum (DBS), 1% penicillin/streptomycin, and 2 mM L-glutamine (Thermo Fisher)) on ice. Tissue fragments were then dissociated through a nylon mesh (70 µm, Merck Millipore) and centrifuged at 440 × *g* for 8 min at 4 °C. The cells were resuspended in fresh growth medium and seeded on T75 cell culture flasks (Cellstar, Greiner Bio-One). After 10 days, the medium was changed twice a week until the cells reached confluence. Three weeks after preparation, cultures typically contained >80% vimentin-positive tanycytes. Primary tanycytes were split using Accutase (Sigma-Aldrich).

### Measurement cAMP and intracellular Ca^2+^ in primary tanycytes

For the cAMP-Glo assay (Promega), primary tanycytes were seeded on 96-well plates (0.4 × 10^5^ cells per well). When the cells reached approximately 80% confluence, the medium was removed, and the tanycytes were stimulated for 30 min with the following compounds dissolved in induction buffer (PBS containing 500 µM IBMX): human CRH (0.021 µM, Ferring Pharmaceuticals), human GHRH (1 µM, Ferring Pharmaceuticals), or forskolin (0.25 µM, Sigma-Aldrich). Controls were treated with the induction buffer only. Cells were then washed with PBS and lysed in lysis buffer. cAMP-Glo detection solution (40 µl provided by the kit) was added to the cell lysate and incubated for 20 min at room temperature (RT). After the addition of Kinase-Glo (80 µl) and incubation for 10 min at RT, luminescence was measured using the Clario Star Plate Reader (BMG Labtech) with a detection wavelength range between 320 and 700 nm.

For [Ca^2+^]_i_ measurements, primary tanycytes were loaded with Fluo4-AM. Cells were seeded on poly-D-lysine-coated cover slips (1 × 10^5^ per well in a 24-well plate) and incubated with Fluo4-AM solution (2 µM Fluo4-AM, 0.02% pluronic 127 (Invitrogen), 2.5 mM probenecid (Thermo Fisher Scientific) in medium) for 30 min at 37 °C and 5% CO_2_. The coverslips were subsequently transferred in a flow chamber and perfused with artificial cerebrospinal fluid (aCSF; 124 mM NaCl, 26 mM NaHCO_3_, 1.25 mM NaH_2_PO_4_, 3 mM KCl, 1 mM MgSO_4_, 2 mM CaCl_2_, 5% CO_2_/95% O_2_) containing 1 mM glucose and 9 mM sucrose at a flow rate of 2 ml/min. After 1 min of baseline recording, primary tanycytes were stimulated with one of the following compounds: 200 µM rat kisspeptin 10 (Kp10, Tocris, #4243), 1 mM oxytocin (Tocris, #1910), 21 µM human CRH (Ferring Pharmaceuticals), 1 mM [Arg^8^]-vasopressin (Tocris, #2935), or 1 mM carbachol (Sigma-Aldrich). The respective stimuli (0.2 µl) were applied over 10 s using a nanoinjector (Nanoliter 2020 Injector, WPI) directly into the recording area, allowing distribution of the compounds across the cells of interest via the perfusion flow. At the end of each experiment, primary tanycytes were stimulated with 333 mM ATP applied via the perfusate as a positive control. Fluorescence was imaged for 5 min at 1 image/s (300 images), using a setup (Till Photonics) mounted on the Axio Examiner D1 upright fluorescence microscope (Zeiss). For analysis, labeled tanycytes were marked as regions of interest (ROI), and the fluorescence intensity of each ROI was normalized to baseline (30 s before stimulation). Cells were only included in the analysis if the fluorescence intensity reached a peak within 60 s after stimulation and subsequently returned to baseline levels.

### Measurement of tanycytic cAMP and Ca^2+^ levels in acute brain slices

Two weeks after injection of *AAV-CAG-GCaMP6s* or *AAV-CAG-Flamindo2*^17,98^ into the lateral ventricle of wild-type mice, brains were dissected, and acute coronal brain slices (180 µm thick) were prepared using a vibrating blade microtome (Leica VT1000S) in ice-cold aCSF containing 1 mM glucose, 9 mM sucrose, 10 mM MgSO_4_ at pH 7.4. For experiments, slices were placed in a flow chamber continuously perfused with oxygenated aCSF (5% CO_2_, 95% O_2_) containing 1 mM glucose and 9 mM sucrose at a flow rate of 2 ml/min. After 1 min of baseline recording, drugs or solvents were applied via two different routes:

1. Rat kisspeptin-10 (Kp-10; 0.2 or 1 mM; Tocris, #4243), kisspeptin-234 (Kp234; 0.8 mM; Tocris, #3881), oxytocin (1 mM; Tocris, #1910), human CRH (21 µM; Ferring Pharmaceuticals), [Arg⁸]-vasopressin (1 mM; Tocris, #2935), somatostatin (50 µM; Tocris, #1157) were puffed to the ventricular zone over 10 s in a volume of 0.2 µl using a nanoinjector (Nanoliter 2020 Injector, WPI). This approach enabled rapid distribution of compounds along the ventricular wall, with subsequent washout via the perfusate.
2. Carbachol (333 µM; Sigma-Aldrich, #C4382), taltirelin (33 µM; Tocris, #2672), clonidine (Sigma-Aldrich, #C7897), baclofen (Sigma-Aldrich, #B-112), epinephrine (Sigma-Aldrich, #E4375), dopamine (Sigma-Aldrich, #H8502) and quinpirole (500 µM, Sigma-Aldrich, #Q111) were applied via the perfusate for 30 s.

For testing inhibitors, slices were preincubated for 15 min with the respective drugs before stimulation. The following inhibitors were used: atropine (10 µM; Sigma-Aldrich, #A0257), muscarinic toxin 7 (MT7; 0.5 µM; Smartox Biotechnology), 2APB (100 µM; TOCRIS, #1224), and U73122 (100 µM; Sigma-Aldrich, #U6756). For concentration-response experiments, slices were sequentially stimulated with increasing concentrations of the respective compound, starting with the lowest concentration. A washout period of at least 15 min was allowed between stimulations to ensure full recovery to baseline. Because of the thickness of the slices and the brief stimulation period, the drug concentrations applied to the tissue likely do not reflect those achieved at the tanycyte membranes.

In *Gα*_*q/11*_^*gliaKO*^ and control mice, we used not GCamP6s but Fura-2 to measure [Ca^2+^]_i_. Acute brain slices were loaded with Fura-2-AM. Slices were incubated with Fura-2-AM (12.5 μg/ml with 0.5% DMSO and 0.05% pluronic 127, Invitrogen) in aCSF containing 1 mM glucose and 9 mM sucrose for 30 min at 37 °C. Then, they were incubated in aCSF containing probenecid (1.25 mM, 30 min 37 °C) and kept at 4 °C until imaging.

Tanycytic [Ca^2+^]_i_ and [cAMP]_i_ were measured using an imaging setup (Till Photonics) mounted on an Axio Examiner D1 upright fluorescence microscope (Zeiss)^17,98^. After intraventricular injection, AAV1/2 vectors transduced tanycytes. We confirmed that tanycytes were the source of the fluorescence signal based on the shape of the cells expressing the fluorescent sensors.

The image series were exported for analysis of fluorescence changes. ROIs corresponding to the different tanycyte subtypes were defined along the ventricular wall. Background fluorescence was measured at a remote tissue site. Using FIJI (ImageJ, version 1.52i), the mean fluorescence intensity of each ROI was determined at each time point, and the background intensity was subtracted. The average fluorescence intensity during the 30 s preceding stimulation was defined as *F*₀, and fluorescence *F* was expressed as *F*/*F*₀. The same strategy was employed for Fura2 measurements, with the quotient between the intensity obtained by excitation with 340 and 380 nm being calculated. To calculate the area under the curve (AUC), the baseline was set to 0.8, and the AUC was determined over 60 s after stimulation. No further baseline or bleaching corrections were performed.

### Imaging glutamate release in tanycyte synaptoids

Two weeks after injection of *AAV-CAG-iGluSnFR3.v857.GPI*^34^ into the lateral ventricle of wild-type mice, brains were dissected and acute brain slices (150 µm thick) were prepared using a vibrating blade microtome (Leica VT1000S) in ice-cold cutting buffer containing (in mM): 92 NMDG, 20 HEPES, 25 glucose, 30 NaHCO_3_, 1.25 NaH_2_PO_4_, 2.5 KCl, 5 ascorbic acid, 3 Na-pyruvate, 2 thiourea, 10 MgSO_4_ × 7 H_2_O, 0.5 CaCl_2_ (305–310 mOsm, pH 7.4). After an additional incubation for 60 min in holding buffer containing (in mM): 92 NaCl, 20 HEPES, 12.5 glucose, 30 NaHCO_3_, 1.25 NaH_2_PO_4_, 2.5 KCl, 5 ascorbic acid, 3 Na-pyruvate, 2 thiourea, 2 MgSO_4_ × 7 H2O, 2 CaCl_2_ (305–310 mOsm, pH 7.4, room temperature), slices were placed in a flow chamber that was perfused with aCSF containing 12.5 mM glucose. All buffers were constantly gassed with 5% CO_2_/95% O_2_. Slices were stimulated with 0.1 mM glutamate or 30 mM KCl for 30 s by bypassing the stimulus into the flow. To block action potentials, TTX (1 µM, Tocris) was added to the aCSF. TTX was washed out for 15 min. Fluorescence changes were obtained with a confocal microscope (Stellaris, Leica) with an imaging rate of 0.5 s per image. To visualize changes in fluorescence, images were converted with FIJI (ImageJ 1.52i) into surface plots with the look-up table Rainbow as false color. As shown in Supplementary Fig. 3a–f, time series were summed up for 350 s.

### Western blotting

For Western blotting, primary tanycytes were seeded into 12-well plates (2 × 10^5^ cells per well) and, after two days, were starved (Dulbecco’s modified Eagle’s medium DMEM/F12 without phenol red, 0.15% insulin, 1% penicillin/streptomycin, and 0.3% putrescin) for 24 h. Cells were washed with PBS and then homogenized in 150 µl lysis buffer while shaking (50 mM Tris-HCl pH 7.5, 1 mM EGTA, 1 mM EDTA, 1% Triton ×-100, 1 mM sodium orthovanadate, 50 mM sodium fluoride, 5 mM sodium pyrophosphate, 0.27 M sucrose, 1 mM phenylmethylsulfonyl fluoride (Carl Roth 6367.2), and 2 µM protease inhibitor (cOmplete, Roche 11836153001). Cell lysates were centrifuged at 18,620 × *g* for 5 min at 4 °C. The supernatant was mixed with SDS buffer (0.75 M Tris-HCl, 0.08 g/ml SDS, 40% glycerol, 0.4 mg/ml bromophenol blue, and 62 mg/ml DTT) in a 3:1 ratio. After samples were subjected to SDS-PAGE, the proteins were transferred to polyvinylidene difluoride (PVDF, Bio-Rad 1620177). Membranes were incubated with primary antibodies against PSD95 and NLGN2 (Supplementary Data File 2) diluted in blocking solution overnight at 4 °C and the respective HRP-conjugated secondary antibodies (Supplementary Data File 2) for 2 h at RT. Finally, we detected the signal using enhanced chemiluminescence (SuperSignal West Pico Substrate, Thermo Scientific, 34580) and a digital detection system (Fusion Solo S, Viber).

### Electron microscopy

To visualize tyrosine hydroxylase (TH)-positive nerve terminals in the median eminence at the ultrastructural level, we used a pre-embedding 3,3’diaminobenzidine-(DAB)-immunoamplification procedure. As described previously^65^, brains of male C57/BL6 mice were perfusion-fixed with 2% PFA, 0.2% picric acid, 0.25% glutaraldehyde in 0.1 M phosphate buffer and post-fixed overnight at 4 °C. Brains were then, cut into 80-μm thick coronal sections on a vibratome in 0.1 M PBS. Brain sections underwent aldehyde inactivation in sodium tetrahydroborate (0.1% in PBS) for 20 min, followed by permeabilization with Triton ×-100 (30 min, 0.05% in PBS). The tissue was incubated for 40 min in a blocking solution containing 1% of BSA and 1% of normal goat serum before overnight incubation at 4 °C in mouse anti-TH clone LNC1 (Millipore MAB318, 1:3000) in incubation buffer (Triton X 0.01%, BSA 0.1%, and normal goat serum 1% in PBS). Sections were then incubated with biotinylated goat anti-mouse IgGs (1:400 in Triton ×-free PBS containing 0.1% BSA, 1% normal goat serum; 2.5 h; room temperature) and post-fixated for 30 min with 2% glutaraldehyde in 0.1 M phosphate buffer. We used avidin-biotin–peroxidase complex (Vector Laboratories, Burlingame, CA, USA; 30 min, room temperature) and a DAB kit (Vector Laboratories, Burlingame, CA, USA) to detect the secondary antibodies. Then, the sections were postfixed with 0.5% OsO_4_ in phosphate buffer for 15 min at room temperature. After dehydrating tissues through a graded series of ethanol steps and embedding in Araldite, semithin sections (1–2 μm thick) were used to progressively approach the median eminence and ultrathin sections (90 nm thick) of the region of interest were collected on Parlodion 0.8%/isoamyl acetate-coated 100 mesh grids (EMS, Fort Washington, PA). The sections were then counterstained with uranyl acetate and lead citrate before imaging. Ultrathin immunolabeled sections were imaged using a Zeiss transmission electron microscope 900 (Leo, Rueil-Malmaison, France) and a Gatan Orius SC1000 CCD camera (Gatan France, Grandchamp, France) at a magnification of 20,000 × *g*.

### Fluorescent in situ hybridization

Mice were perfused with 4% PFA and 40-µm thick coronal brain sections were prepared. In situ hybridization was performed using RNAScope Multiplex Fluorescent Reagent Kit v2 (ACD). When hybridizing with probes for *Agrp* (#400711-C3, NM_001271806.1), *Chat* (#408731-C2, **NM_009891.2)*, Crh* (#316091-C2, NM_205769.2)*, Ghrh* (#470991-C2, NM_010285.2)*, Kiss1* (#500141-C3, NM_178260.3)*, Pomc* (#314081-C2, NM_008895.3)*, Slc17a7 (Vglut2;* #319171-C3, NM_080853.3*), Slc32a1 (Vgat;* #319191, NM_009508.2*), Sst* (#404631-C3, NM_009215.1), and *Trh* (#436811-C2, NM_009426.3), sections were washed in PBS before mounting on Superfrost slides and drying. Then, sections were dehydrated with increasing ethanol concentrations, baked in the hybridization oven (HybEZ II Oven, ACD, 37 °C, 30 min), and incubated first in hydrogen peroxide solution (10 min at RT) and then in Protease IV solution (20 min, RT). For in situ hybridization to *Kiss1r* (#408001, NM_053244.5), *Trhr1* (#443771-C2, NM_013696.2), *Chrm1* (#495291, NM_001112697.1), *Sstr2* (#437681-C3, NM_001042606.2), *Oxtr* (#412171, NM_001081147.1) and *Avpr1a* (#418061-C3, NM_016847.2), a different pre-treatment protocol was employed. After washing off cryoprotectants with Tris-buffered saline (TBS), sections were incubated in H_2_O_2_ (45 min, RT). Then, sections were mounted on Superfrost slides and dried at 60 °C for 3 h. RNAScope Target Retrieval was performed for 10 min in boiling solution (provided by kit). Sections were shorty washed in H_2_O and dried. Then, sections were incubated with protease plus for 8 min before washing with H_2_O. For hybridization, sections were incubated in C2- and/or C3- probes (diluted 1:50 either in C1-probes or, if no C1-probe was used, in probe diluter) in the hybridization oven (40 °C for 2 h), washed in RNAScope Wash buffer, and stored in 5× SSC overnight at 4 °C. The next day, the sections were incubated successively with AMP1, AMP2, and AMP3 (15–30 min, 40 °C), with washing steps in between. Then, the sections were incubated successively with HRP-C1, if C1-probe was used, HRP-C2, and HRP-C3 (15 min, 40 °C), followed by incubation with a fluorescent marker (TSA Plus Cyanine 3 or 5, 30 min, 40 °C) with washing and HRP blockade in between. After in situ hybridization, immunohistochemistry for RFP, GFP, or vimentin was performed. Nuclei were stained with DAPI (1 µg/ml) during incubation with the secondary antibody. Sections were mounted using Mowiol–Dabco. Images were obtained with a confocal microscope (Stellaris, Leica).

### Immunofluorescence staining

Deeply anesthetized mice were transcardially perfused with PBS containing heparin (10 IU/ml), followed by 4% PFA. Brains were then postfixed in 4% PFA for 6–7 h at 4 °C. For vibratome sectioning, 50-µm thick brain sections were cut using a vibrating blade microtome (Leica VT1200S). Sections were stored at −20 °C in antifreeze (13.09 mM NaH_2_PO_4_, 38.4 mM Na_2_HPO_4_, 33% ethylene glycol, 33% glycerol, and 33% distilled water). After washing in PBST (PBS, 0.3% Triton ×-100), sections were blocked in a 5% solution of serum from the host of the secondary antibody in PBST for 2 h at RT, incubated with primary antibodies overnight at 4 °C, and then with secondary antibodies, including DAPI (1 µg/ml) for 2 h at RT (Supplementary Data File 2). After three washes in PBST, sections were mounted using Mowiol–Dabco. In the case of two rabbit primary antibodies, sections were sequentially stained with additional blocking steps (4% rabbit serum in PBST) and anti-rabbit Fab fragment (1:100 in PBST, Jackson ImmunoResearch), each 1 h at RT with washing steps in between, after the first secondary antibody.

### Imaging and image analysis of synaptoids

For fluorescence microscopy, we used an Axio Imager.Z2 ApoTome (Zeiss, Jena, Germany) or a Stellaris 5 confocal microscope (Leica) equipped with a 63× objective (HC PL APO 63×/1.40 OIL CS2). Before processing images with Imaris, images were processed with the Leica software “Lightning” to reduce background. Tanycytic surfaces were reconstructed using the surface tool, while postsynaptic marker proteins were reconstructed using the spots tool. Synaptic protein spots were then filtered for those in close contact with the tanycytic surface (Threshold distance: SHANK2 to tanycyte surface −0.5 to −0.01 µm, NLGN2 to tanycyte surface −0.15 to +0.15 µm). Tanycyte surface was virtually cut into sections (cell body, then every 30 µm, after 120 µm “rest”). The number of closely adjacent spots was normalized to the tanycyte surface area. Imaris processing was performed independently by two researchers.

A custom-built STED microscope (Abberior GmbH) was used for super-resolution microscopy. The setup included a 560- or 640-nm diode excitation laser, a 775-nm STED laser, both pulsed at 40 MHz, and a 1.4 NA objective (UPlanSApo 100×/1.40 oil, Olympus). A z-scanning piezo (PIFOC, Physik Instrumente) was used for focusing, and a spatial light modulator (Hamamatsu) was used in the depletion beam to generate a top-hat (3D-STED) phase mask. Sample induced aberrations were corrected using a deformable mirror. Images were acquired using the settings described in Supplementary Data File 3. Images acquired with the STED microscope were first deconvolved using the Richardson-Lucy algorithm (RL value = 3) with the “DeconvolutionLab2” plugin (FIJI) and then drift-corrected with the StackReg plugin (FIJI).

To determine the relative spatial proximity of synaptoid puncta and tanycyte membrane, an intensity profile of synaptic protein and tanycyte membrane was generated and plotted as normalized data.

Imaris software was used for the analysis of 3D-STED synaptoid puncta^99^. Surfaces were first generated for both tanycytes and synaptic protein markers. Synaptic protein surfaces were then filtered for synaptoids. For post-synaptoid proteins, its surface must touch the tanycyte surface defined by the plasma membrane and a minimum of 60% of the volume must lie within the tanycyte surface. For presynaptic proteins, surfaces were selected if at least 40% of the surface volume was outside the tanycyte surface and both surfaces were in contact.

Tanycyte subpopulations were classified according to previously published criteria^11^ based on the proximity to hypothalamic nuclei and regions, and the structures they contact; for brevity, the traditional names (α1, α2, β1, β2) were used.

### Optimal transport colocalization analysis (OTC)

After immunofluorescence staining of the tanycyte membrane marker MCT8, the excitatory postsynaptic markers SHANK2 and HOMER1, or the inhibitory postsynaptic marker NLGN2 in wild-type brain sections, we performed 2D-STED for image acquisition. STED images were deconvolved using the Abberior TRUESHARP deconvolution tool. OTC was analyzed and plotted for seven individual ROIs (600 × 600 nm, 0.36 µm^2^, 40 nm/pixel). We performed the computations with the OTC package (v 1.0)^30^ in R (v 4.3.2) using IBM ILOG CPLEX Optimization Studio (v 22.1.1) solver. Signals of the colocalized proteins SHANK2 and HOMER1 served as positive control, while as a negative control, a further seven individual ROIs, in which the fluorescence intensities between MCT8 and SHANK2 did not overlap in line plots, were selected from the same larger image.

### Study of the estrous cyclicity

The estrous cycle of the mice was determined between 10:00 and 11:00 am daily by a microscopic observation of the cell types present in the vaginal smear as previously described^100^. A fully successful cycle (4–5 days length) was considered when a proestrus was followed by 1 or 2 estrus and then diestrus (we considered diestrus to be both metestrus and diestrus 2). Except when indicated, all the blood sampling and end-point experiments were performed in estrus. Mice of different experimental groups were co-housed in the same cages. Experimenters were blinded to group allocation in the phenotype analysis.

### Tail-tip bleeding and LH measurements

Mice were acclimated to daily handling for at least 2 weeks prior to blood collection. For tail-tip blood sampling^101^, a small cut was made in the tail tip, and blood (3 µl) was collected at 6 or 10-min intervals. The blood sample was immediately diluted in 60 µl PBS (0.01 M, pH 7.4, containing 0.05% Tween 20), mixed, and stored at −80 °C until measurement. For investigating basal LH pulsatility, a 3-h tail-tip blood sampling was conducted in male mice starting 1 h after lights on in the morning. Female mice were cycled for 15 days before a 2-h tail-tip blood sampling was performed during the morning of estrus.

For the kisspeptin response assay, a 2-h and 20-min tail-tip blood sampling at 10-min intervals was performed in the morning of diestrus. Four basal tail-blood samples were obtained before administering CNO (Tocris/Bio-Techne #63295, 1 mg/kg) or saline i.p. to the mice. After 10 min, a tail blood sample was collected just before the injection of Kisspeptin-10 (Kp10; GeneCust YY-10-NH2, 1 nmol/mouse) diluted in saline (100 μl). After the Kp10 injection, the mice were bled for an additional 90 min. After one week of rest, mice were tip-tail bled again but received saline if CNO had been administered in the first round, and CNO if saline had been administered before.

Circulating LH levels were measured with a well-established ultrasensitive ELISA^102^. Briefly, 96-well high-affinity binding plates (CLS3369, Corning®) were coated with a bovine LHβ518B7 monoclonal antibody (1 µg/ml in PBS; 50 μl per well; UC Davis, #518B7). The detection antibody 5303 SPRN-5 (Medix Biochemica, #100588) was biotinylated using EZ-Link NHS-PEG4 Biotinylation Kit (Thermo Scientific, #PI21455)^103^ and was added in a concentration of 4 µg/ml to the wells. The functional sensitivity of the assay was 15 pg/ml. Intra- and inter-assay variations were 7 and 15%, respectively. Absorbance was measured in a Varioskan LUX plate reader (Thermo-Fisher).

To characterize pulsatile LH secretion, we analyzed pulse frequency, inter-pulse interval, pulse amplitude, basal release, and area under the curve (AUC), as previously reported in mice^21,104^. Briefly, an LH pulse was manually identified as *a* > 20% increase from one of the two previous time points, followed by *a* > 10% decrease in one of the subsequent time points. Additionally, to qualify as a pulse, the pulse amplitude must be 1.5 times the standard deviation of the average LH levels. If any pulse amplitude met these conditions, the frequency was considered zero. The results of LH pulse frequency are reported as the number of pulses per 60 min. The pulse interval (aka pulse duration) was defined as the average time between the start and end of each independent pulse. Pulse amplitude was defined as the average magnitude of the increase in blood LH levels for each pulse (the difference between the nadir and the maximum blood LH levels at each peak). The basal LH level was defined as the average of all nadir LH values. The AUC was determined for each LH profile; the AUC represents the total LH increase in the blood during the analyzed period.

### Bi-lateral ovariectomy (OVX)

Adult mice were anesthetized with isoflurane (3%) in an induction chamber and placed on a surgical table equipped with a mask to maintain anesthesia during the procedure (1% isoflurane). After the i.p. injection of carprofen (5 mg/kg), ovaries and uterine horns were exteriorized through a dorsal incision in the skin and the ovary, vessels, fat, ligament, and oviduct surrounding the ovary were ligated and cut with the help of an electrocautery. After suture, mice were placed in a clean cage, and body core temperature was maintained at 37 °C with an electrical blanket until mice recovered from the anesthesia. An additional injection of carprofen (5 mg/kg) was performed in mice that presented signs of discomfort the morning after the surgery. Fifteen days after OVX, the ME was dissected to perform FACS of tanycytes.

### Isolation of tanycytes using fluorescence-activated cell sorting (FACS)

Mice received a stereotaxic injection of a 1:1 mix of either (1) *AAV-Dio2-Cre* plus *AAV-flex-shKiss1R-GFP*, (2) *AAV-Vcan-Cre-2A-GFP* plus *AAV-flex-shKiss1R-GFP;* (3) *AAV-Dio2-Cre* plus *AAV-flex-shScrambled-GFP*, or (4) *AAV-A2m-Cre-2A-GFP* plus *AAV-flex-shKiss1R-GFP*. After 4 to 5 weeks, ME were microdissected and enzymatically dissociated using a Papain Dissociation System (Worthington, Lakewood, NJ) to obtain a single-cell suspension that was directly used for FACS as described elsewhere^18,19,105^. FACS was performed using an ARIA SORP cell sorter cytometer (BD Bioscience, Inc). The sorting parameters were based on GFP fluorescence (excitation, 488 nm, 50 mW; detection, GFP bandpass 530/30 nm, autofluorescence bandpass 695/40 nm). Autofluorescence was compensated by comparing fluorescence in ME cell suspensions and cerebral cortex in which genetic recombination did not occur, as illustrated in Supplementary Fig. 13a. For each mouse, a minimum of 500 GFP-positive and 500 GFP-negative cells were sorted directly into lysis buffer (10 μl containing 0.1% Triton ×-100 and 0.4 unit/μl RNaseOUT, 10777019, Invitrogen).

### Quantitative real time PCR analyses

RNA from 500 to 1000 FACS-sorted GFP-positive and GFP-negative cells were reverse transcribed using High-Capacity cDNA Reverse transcription kit (#4368814, Applied Biosystems™) after DNAse treatment (#18068015, DNase I, Amplification Grade, Invitrogen™). After a linear preamplification step was performed using the TaqMan® PreAmp Master Mix Kit (4488593, Applied Biosystems™), real-time PCR was carried out on QuantStudio 3 Thermo-Fisher Real-Time PCR System using the TaqMan™ Universal Master Mix II (4440049, Applied Biosystems™) and the TaqMan® probes: *Kiss1r* (Mm00475046_m1), *Vimentin* (Mm01333430_m1), *Elavl3* (Mm01151962_m1), and *Pecam1* (Mm01242576_m1). Control housekeeping genes *18S* (Mm03928990_g1) and *Actb* (Mm00607939_s1) were used for analysis. Gene expression data were analyzed using the 2^−ΔΔCt^ method.

### Whole-mount immunolabeling, tissue-clearing, and lightsheet microscopy

Two weeks after unilateral injection of *AAV-CMV-flex-mGFP* into the AVPV of *Kiss1-Cre* mice, Kiss1 neurons expressed mGFP. Mice were intracardially perfused with 4% PFA in PBS (0.01 M, pH 7.4). The brains were post-fixed in the same fixative solution overnight and processed with an adapted iDISCO+ protocol^106,107^. Brains were gradually dehydrated with increasing concentrations of methanol (20, 40, 60, and 80% methanol in PBS, followed by 2 × 100% methanol, one hour each), delipidated overnight in 66% dichloromethane/33% methanol at 4 °C with agitation, rinsed twice in methanol, and bleached overnight at 4 °C in methanol with 5% H_2_O_2_. After gradual rehydration (100, 80, 60, 40, and 20% methanol in PBS, followed by 2 rinses in PBS, one hour each), the samples were incubated for 4 days at 37 °C with rotation in permeabilization/blocking solution containing 1% Triton ×-100, 0.2% gelatin, and 0.05% sodium azide in PBS (PBS-GT). The samples were further incubated with primary anti-GFP antibodies in PBS-GT for 10 days at 37 °C with rotation, rinsed 6 × 1 h in PBS + 1% Triton ×-100, and incubated for 7 days with secondary goat anti-rabbit antibodies (Alexa Fluor 647) in PBS-GT at 37 °C with rotation. Brains were next washed 6 × 1 h in PBS + 1% Triton ×-100 to remove free antibodies. For tissue clearing, the samples were first gradually dehydrated with increasing concentrations of methanol (20, 40, 60, and 80% methanol in PBS, followed by 2 × 100% methanol, one hour each). Methanol was washed out with an overnight incubation in 66% dichloromethane/33% methanol at 4 °C with agitation, followed by 2 × 1 h in 100% dichloromethane at 20 °C with agitation. The samples were finally transferred to dibenzylether (DBE) for refractive index matching during 2 h at 20 °C with rotation. A fresh solution of DBE was used for storage and samples were kept at 20 °C in the dark until imaging.

3D Imaging was performed on the Ultramicroscope I (LaVision BioTec) equipped with a 1.1×/0.1NA objective and an Andor Neo 5.5 sCMOS camera. The light sheet was generated by a laser (wavelength 488, 568, or 647 nm, Coherent Sapphire Laser, LaVision BioTec) and two cylindrical lenses. Samples were placed in an imaging reservoir made of 100% quartz (LaVision BioTec) filled with DBE and illuminated from the side by the laser light. ImspectorPro software (v5.1.363, LaVision BioTec) was used for image acquisition: the z-step was set to 5 µm and the laser numerical aperture was set to 0.030. The resulting sequences of tiff files were processed with Imaris Converter v9.6 and Imaris Stitcher v9.6 (Oxford Instruments). Imaris 10.1 (Oxford Instruments) was used for visualization and analysis of the datasets.

### scRNAseq data processing

Two scRNAseq databases of mouse tanycytes, Hypomap and Tany-seq, were combined to the unified database Tanybase^23,27^. Overall, Tanybase is based on 18 studies including data from 158 mice of various ages that were fed different diets, as well as 34 slices containing the suprachiasmatic nucleus. From the Hypomap database, all cells named as tanycytes either by the original study or by Hypomap were subsetted. We removed cells from the Tany-seq database that were included in Hypomap^22,26^. Hypomap and Tany-seq databases were combined using shared parameters, including diet, age, tanycyte subclusters, original identity, and dataset. Cell ids were unified and duplicates were cleaned. Cells with more than 15% mitochondrial transcripts, total RNA count below 600, and unique features less than 200 were removed. We included only cells that labeled as tanycyte according to the Allen Institute MapMyCells software (RRID:SCR_024672). For integration, Tanybase was split into different layers for each dataset containing more than 100 cells and datasets that have less than 100 cells were combined (“others”). Tanybase was scaled, normalized, and variable features were determined using Seurat functions. Principle-component analysis was performed as well as integration for batch correction using canonical correlation analysis. After the integration, layers were re-joined. For dimensionality reduction, we performed UMAP clustering with FindClusters and FindNeighbors functions. Labels were transferred from corresponding databases; unlabeled cells were labeled using module scores and the markers provided in Tany-seq^27^. For Tanybase, we used Seurat (v.5.2.0), AnnData, Matrix packages. Plots were generated with ggplot2 and Seurat packages.

### Statistics

Data were analyzed using GraphPad Prism 8 and 10 (GraphPad Software) and SPSS 28 (IBM). Significance was considered when *p* < 0.05. Depending on the dataset and experimental design, different statistical methods were used as indicated in the figure legends. Parametric statistics (e.g., *t*-Test, ANOVA) were only applied if assumptions were met, i.e., datasets were examined for Gaussian distribution by D’Agostino-Pearson test, aided by visual inspection of the data, and homogeneity of variances by Brown–Forsythe, Levene’s, or *F*-test (depending on the statistical method used). If assumptions for parametric procedures were not met or could not be reliably assumed, non-parametric methods were used as indicated. Two-tailed tests were applied if not indicated otherwise. Greenhouse-Geisser correction was used in ANOVA statistics if the sphericity assumption was violated (Mauchly test). Detailed statistical information is provided in Supplementary Data File 5. No data points were excluded if not stated otherwise in Supplementary Data File 5.

### Reporting summary

Further information on research design is available in the Nature Portfolio Reporting Summary linked to this article.

## Supplementary information

Supplementary File

Description of Additional Supplementary Files

Supplementary Data file 1

Supplementary Data file 2

Supplementary Data file 3

Supplementary Data file 4

Supplementary Data file 5

Supplementary Video 1

Supplementary Video 2

Supplementary Video 3

Supplementary Video 4

Supplementary Video 5

Supplementary Video 6

Supplementary Video 7

Supplementary Video 8

Reporting Summary

Transparent Peer Review file

## Source data

Source Data

## Data Availability

The Tanybase data are publicly accessible in the repository Mendeley data, 10.17632/p6jkzkpdd6.1. Other datasets generated during the current study are included in the Source data file. The plasmids used to generate the AAVs and their genetic sequences will be provided by the corresponding author M.Sc. upon request. Source data are provided with this paper.

## Code availability

The code used for Tanybase is publicly accessible on GitHub, https://github.com/umitozorhan/Tanybase with the 10.5281/zenodo.19912027^108^.
