# Supplementary material for: A synaptoid connectome differentiates tanycytic subpopulations and underlies neuroglial communication and neuroendocrine regulation
Source: Nat Commun. 2026 Jun 20;17:7797. doi: 10.1038/s41467-026-74598-5 (PMC13439102; doi:10.1038/s41467-026-74598-5)
Supplement: Supplementary file 2 [file 41467_2026_74598_MOESM17_ESM.pdf]

nature portfolio

Peer Review File

A Synaptoid Connectome Differentiates Tanycytic Subpopulations and Underlies Neuroglial Communication and Neuroendocrine Regulation

Corresponding Author: Professor Markus Schwaninger

This file contains all reviewer reports in order by version, followed by all author rebuttals in order by version.

Version 0:

Reviewer comments:

Reviewer #1

(Remarks to the Author)
This is an interesting report that provides unprecedented detail regarding neuronal connection to tanycytes lining the 3rd ventricle and reveals much wider spread and more specific connections to subpopulations of these unique glia. A functional role in the control of LH pulsatility and release amplitude increases the significance of the findings. Some improvements in presentation would further impact.
1) The expertise of the authors in reproductive neuroendocrinology is evident in the scholarly review of the literature and detailed discussion of many nuanced aspects of the control of LH release. This strength is also a weakness in that there seems to be an assumption that all readers of Nature Communications are familiar with the neural control of the anterior pituitary and in particular the HPGA, including that there is both pulsatile and basal release of LH, as well as an LH surge in females which is never mentioned. It is also assumed that readers are familiar with the ME and its anatomy, and the role of tanycytic endfeet. However, many readers are not and will be quickly confused by the introductory portions of the manuscript. Framing early on the unique anatomy and physiology of this region, and perhaps focusing solely on the GnRH system, would increase accessibility.
2) Related to the above, the manuscript appears to be trying to achieve two things at once. First is to say the projections to tanycytes are far and wide, and second they are highly specific. Both can be true at the same time but as presented it is a bit head spinning. A clearer framing of what are the important discoveries here would help.
3) There is no mention of a role for neuronal synaptoids on tanycytes in the control of the HPAA yet there are strong projections from the PVN. Is this an omission or is there actually no role? This would be an important specificity to highlight if the latter.
4) The lack of consideration of sex of the animals and any discussion of the sex-specific LH surge in females is a noticeable gap given the HPGA is one of the more dimorphic systems in the brain. At the least the sex of the mice should be mentioned for all experiments, not just those involving the estrus cycle, and a more systematic comparison between males and females included.
5) The introductory section on the number of puncta of various synapse related proteins is too “in the weeds” with excessive amounts of detail that do not provide any functional relevance to the findings. The number of references to supplemental figures far out weighs those to primary figures, again risking the loss of interest in the confused reader.
6) Related to the above, the reader is not provided any concept of how the synaptoids on the tanycytes could regulate LH release until the Discussion and then only vaguely so, with mention of the movement of the endfeet. Again most readers will not immediately grasp the idea that the Ca++ changes could be having such an effect nor how the endfeet are regulating the release of GnRH, not LH directly.
7) The statistical tests used, in the limited cases in which they are, are not mentioned and no F values or d.f.s provided following ANOVA.

(Remarks on code availability)

Reviewer #2

(Remarks to the Author)
In this very exciting study, Neve et al. establish that neurons can communicate with tanycytes via a synaptic-like structure and demonstrate the functional significance of this communication. This is a fundamental discovery that represents a major advance in our understanding of brain function and brain–body communication.
Using sophisticated genetic approaches (including viral tracing), super-resolution imaging, scRNA-seq, DREADDs, calcium, cAMP, and glutamate imaging, as well as endocrine assays, the authors demonstrate the presence of abundant neuron–tanycyte contacts resembling classical neuronal synapses — “synaptoids.” They characterize the neuronal populations that form these contacts with tanycytes and show that synaptoids play essential roles in regulating kisspeptin’s effects on LH release and pulsatility, as well as on reproductive functon(e.g., the estrous cycle).

There is an overwhelming amount of novel and exciting data presented, the methodology is elegant, and the conclusions are paradigm-shifting. There are only few minor suggestions:

1. The sample size is not always clearly indicated. For example, for fig 1c – scRNAseq, how many samples (mice) were analyzed? I couldn’t find this information in supplemental tables or the method section.
For figure 1j showing calcium imaging, based on the extended table 4 it seems like it was done in one mouse, is that the case? If so, the sample size should be increased.
Figure 3c includes 2 females and 1 male. It would be stronger to have a larger sample size to enable comparison between sexes.
Fig 3d-m – based on the table, it seems that the data represent 2 animals. The legend states that the data were from 5 mice, but it’s unclear if every agonist was tested in 5 mice? If not, the sample size seems too low. Also, quantification of these effects would be helpful.
Same concerns for fig 4f and g, is there sex differences? For 4h, what’s the n, are there sex differences, can the number of traced neurons be quantified?

2. The ex vivo experiments involving neuropeptide application were conducted using concentrations that would not be expected under physiological condition. For example, 1 mM of Kp10 was applied to brain slices, whereas only 1 nmol per 25g mouse (more than 100-fold lower) was used to stimulate LH secretion in vivo. Similar discrepancies apply to other neuropeptides used in the study. The authors should discuss a rationale for the doses used—for instance, if such high concentrations could be reached locally near the release site (e.g., within the synaptic cleft). If that is not the case, it would be important to discuss tanycyte-mediated effects of these peptides under physiological conditions.

(Remarks on code availability)

Reviewer #3

(Remarks to the Author)
Summary:
The study by Nava and colleagues characterized the heterogeneity of neuronal and the tanycytic populations by studying the synaptoid connectome between these two populations. The researchers examined this synaptoid connectivity by utilizing high-resolution microscopy to identify post-synaptic proteins residing in tanycytes, rabies monosynaptic retrograde tracing to identify the unique neuronal inputs to subpopulations of tanycytes, Ca^{2+} imaging to functionally confirm tanycyte responsiveness to various neurotransmitters and neuropeptides, and genetic screening to confirm tanycytic transcriptional heterogeneity. They further investigate the Kiss1 neuron population and its connection to tanycytes in the context of reproductive function. The authors propose subpopulations of tanycytes may differentially regulate the effects of kisspeptin on downstream LH release patterns. Much of this work is carefully done and detailed that considerably expands our understanding of the extent to which glia are integrated in neuronal networks and their contribution to physiological behavior. Some of the stronger statements about the control of LH release will likely require additional studies and/or explanation:

Comments:
1) The authors make the criteria for distinguishing between β and α tanycytes clear, but what were the criteria for distinguishing β1 from β2 (and α1 from α2) tanycytes in Extended data Fig. 1c-h? It will be useful to explicitly state these criteria given the conclusions about some of the effects on reproduction being specific to β2 tanycytes.
2) The authors suggest that β2 regulate basal LH release based on the different effects of DIO::shKiss1R and Vcan::shKiss1R. What is meant by basal LH release (also mentioned in abstract and discussion)? Odd term to describe a pulsatile system. Is there a specific promoter for β2 tanycytes to directly assess effects on this mode of LH?
3) Given that the viruses are injected into the lateral ventricles, have the authors checked for “off target” expression of these novel AAVs across all ventricular space (e.g., subventricular zone)?
4) Colocalization of some markers with Rbv (e.g., kiss, VGAT AVP ChAT TH) is clear, but other markers are difficult to visualize as double labeled (Vglut) as there is a general background fluorescence in this channel. Adding DAPI or other nuclear marker might help with this. Extended data Fig. 4e, f, and g, it is difficult to visualize and confirm coexpression of the makers when only the merged channels are shown. Green arrow in ext data 4 is not defined.

5) What was the methodology for calculating the ΔF/F0 for the calcium imaging experiments? E.g., how was bleaching corrected, how was baseline determined, was any post-hoc smoothing done?
6) Fura-2-AM is mentioned in the methods for the Ca2+ level detection. Fluo4 is also mentioned in Extended data Fig. 7. However, the bulk of the data appear to be with AAV-CAG-GCaMP6s . What is the rationale for using all of these calcium sensor indicators? There do not appear to be any studies included with Fura2.
7) The use of AAV to deliver specific promoter-driven cres does a nice job of distinguishing different populations of tanycytes. Activation of hM4Di blunted LH release when Dio and A2m cells, but not Vcan cells, were targeted. Interesting finding but the effect is mild. The authors need to discuss the different timing of the peak in LH between the DIo2 and A2m mice and also the increase in LH in the CNO treated mice at the final timepoint, is there a different physiological effect at a delay? These data should be on the same scale to facilitate comparison. The CNO-induced LH release from A2m::hM4Di mice in Fig. 5c is dramatically lower and has different kinetics than in 5a and 5b (not to mention the differences in kinetics between 5a and 5b, especially the LH rise in the hM4Dq group). Is it possible other non-tanycytic populations along the ventricles are contributing to these differences in LH patterns? If not, what other explanations are there for these differences, especially between the saline + Kp10 mice across 5a, b, and c? Perhaps there is CNO-independent activation of the hM4Di receptors?
8) Blood sampling was performed during estrus for female mice. The patterns observed are substantially different than published work, which report 1 pulse/4h for LH (doi: 10.1210/en.2016-1351) or 1 calcium event per 10h in vivo for arc kiss neurons (doi: 10.1210/en.2019-00193). Information on assay sensitivity and pulse detection algorithms must be provided (the decay phase of some pulses is quite abrupt 5i, 5n) and at minimum an explanation provided for the different results. It is also puzzling why A2m targeted cells were not included given that is what was mediating effects in diestrus and that these cells are closer to GnRH terminals in the median eminence. Ideally, the studies should be repeated on diestrus and A2m-targeted tanycytes included because the statement on line 359 that "α+β1 tanycytes exert a Kiss1-mediated slowdown of GnRH pulsatility" requires rigorous support as kisspeptin has been viewed as an activator in this system, as do the statements on lines 430-435 of the discussion.
9) The term LH baseline is used in Fig. 5, how is this calculated? Is this perhaps interpulse nadir (baseline like basal an odd term for a pulsatile system). Reporting LH data as AUC (ext data 10) is not helpful as both frequency and amplitude information are eliminated and these are critical elements for assessing neuroendocrine activity.
10) Cycle success is quite variable in controls- 50% in one study and 75% in another, suggesting cycles in these mice are already poor. Were these mice verified to be cycling before treatment. The more traditional method of evaluating % days spent per stage does not reveal a difference, although % data should not be compared with t test, more appropriate to use chi square or Fisher exact.

Other points
L77-78 it is an overstatement that the connection of tanycytes to specific neurons is unique among glia cells as not all glial populations have been investigated

L118, citation missing

L141 TTX attenuated not prevented

L143 response is not dependent on TTX but on what TTX blocks

L379-381 The view that glia are no integral components of neural networks is outdated. For example, the tripartite synapse emerged as a concept about 30 years ago.

L382 the data clearly demonstrate very interesting projections and interactions for tanycytes with glia but other types of glia have also been shown to functionally interact with neurons, so unusual is not an accurate term.

(Remarks on code availability)

Version 1:

Reviewer comments:

Reviewer #1

(Remarks to the Author)
The authors had done an excellent job responding to both mine and other reviewers concerns, I remain enthusiastic about this manuscript

(Remarks on code availability)

Reviewer #2

(Remarks to the Author)
The authors have adequately addressed all my comments. I believe the manuscript is now suitable for publication.

(Remarks on code availability)

Reviewer #3

(Remarks to the Author)
The authors have included new data, analyses and images and rewritten aspects of the manuscript. These result in a markedly improved presentation of this work, which is important and impactful. The addition of the sex comparisons is notable, but the authors should indicate they've chosen to compare the postovulatory state in females (estrus) to males, rather than an arguably less sexually differentiated state. Future work examining other cycle stages and sex differences for these interesting findings would be welcome.

(Remarks on code availability)
I have no expertise in code.

Open Access This Peer Review File is licensed under a Creative Commons Attribution 4.0 International License, which permits use, sharing, adaptation, distribution and reproduction in any medium or format, as long as you give appropriate credit to the original author(s) and the source, provide a link to the Creative Commons license, and indicate if changes were made.
In cases where reviewers are anonymous, credit should be given to 'Anonymous Referee' and the source.
The images or other third party material in this Peer Review File are included in the article's Creative Commons license, unless indicated otherwise in a credit line to the material. If material is not included in the article's Creative Commons license and your intended use is not permitted by statutory regulation or exceeds the permitted use, you will need to obtain permission directly from the copyright holder.
To view a copy of this license, visit https://creativecommons.org/licenses/by/4.0/

We thank the reviewers for their insightful comments, which helped improve the manuscript. After conducting additional experiments, we addressed all reviewer comments in the revised manuscript, as outlined below. The main additional experiments are as follows:

- RbV tracing experiments in mice of both sexes
- Ca^{2+} imaging in mice of both sexes
- Investigation of the effect of a Kiss1R antagonist on Ca^{2+} stimulation by Kiss1
- Investigation of LH pulsatility and cyclicity in mice with A2m-targeted *Kiss1r* knockdown

Reviewer #1 (Remarks to the Author):

Reviewer #1: "This is an interesting report that provides unprecedented detail regarding neuronal connection to tanycytes lining the 3rd ventricle and reveals much wider spread and more specific connections to subpopulations of these unique glia. A functional role in the control of LH pulsatility and release amplitude increases the significance of the findings. Some improvements in presentation would further impact."
Response: We thank the reviewer for the positive evaluation of the manuscript and for the guidance in improving its impact.

Reviewer #1: "1) The expertise of the authors in reproductive neuroendocrinology is evident in the scholarly review of the literature and detailed discussion of many nuanced aspects of the control of LH release. This strength is also a weakness in that there seems to be an assumption that all readers of Nature Communications are familiar with the neural control of the anterior pituitary and in particular the HPGA, including that there is both pulsatile and basal release of LH, as well as an LH surge in females which is never mentioned. It is also assumed that readers are familiar with the ME and its anatomy, and the role of tanycytic endfeet. However, many readers are not and will be quickly confused by the introductory portions of the manuscript. Framing early on the unique anatomy and physiology of this region, and perhaps focusing solely on the GnRH system, would increase accessibility."
Response: We gratefully accept this advice and have included explanatory text on the neural control of the anterior pituitary in the introduction (page 3, line 20 – page 4, line 7).

Reviewer #1: "2) Related to the above, the manuscript appears to be trying to achieve two things at once. First is to say the projections to tanycytes are far and wide, and second they are highly specific. Both can be true at the same time but as presented it is a bit head spinning. A clearer framing of what are the important discoveries here would help."
Response: We thank the reviewer for the suggestion to structure the results in this way. The reviewer has indeed identified the two most important conclusions of the manuscript. We believe that highlighting this point will help readers and have included this point in the revised manuscript (abstract; page 4, lines 16-18; page 21, line 19 – page 23, line 2).

Reviewer #1: "3) There is no mention of a role for neuronal synaptoids on tanycytes in the control of the HPAA yet there are strong projections from the PVN. Is this an omission or is there actually no role? This would be an important specificity to highlight if the latter."

Response: The point is well taken. Retrograde tracing experiments identified both CRH and vasopressin neurons in the PVN that project to tanycytes. To illustrate this more clearly, we have replaced the image showing *in situ* hybridization of CRH in retrogradely labeled PVN neurons (Supplementary Fig. 6d). Additionally, both peptides induced a marked Ca^{2+} response in tanycytes, which has been quantified during the revision (Fig. 3i, j). Based on these findings, we have followed the reviewer's suggestion to discuss the evidence that tanycytes regulate the hypothalamic-pituitary-adrenal axis (page 24, lines 7-11).

Reviewer #1: "4) The lack of consideration of sex of the animals and any discussion of the sex-specific LH surge in females is a noticeable gap given the HPGA is one of the more dimorphic systems in the brain. At the least the sex of the mice should be mentioned for all experiments, not just those involving the estrus cycle, and a more systematic comparison between males and females included."

Response: We thank the reviewer for raising this important issue. The sex of all animals is listed in Supplementary Table 5. In response to the reviewer's comment, we performed additional RbV tracing experiments to enable comparison between the sexes. Interestingly, the distribution of retrogradely labeled neurons did not differ between the sexes when tanycytes were targeted as starter cells using either the *GlastCreER*^{T2} mouse line or the *AAV-Vcan-Cre-2A-GFP* vector (Supplementary Fig. 4). Additionally, we analyzed the effect of kisspeptin on $[Ca^{2+}]_i$ in slices of the mediobasal hypothalamus from both sexes, but we found no statistically significant difference (Fig. 3e). However, the effect of knocking down *Kiss1r* in *A2m*-targeted β-tanycytes on basal LH levels and pulse amplitude differed between female and male mice (Fig. 5q, r; Supplementary Fig. 13aa-ae). This sexual dimorphism may reflect stronger Kiss1 input from the AVPV in female mice, which are known to have more Kiss1 neurons in the AVPV than males (Clarkson and Herbison, 2006). In the revised manuscript, we discuss how neuron-tanycyte connections exhibit sexual dimorphism (page 19, lines 7-13).

Reviewer #1: "5) The introductory section on the number of puncta of various synapse related proteins is too "in the weeds" with excessive amounts of detail that do not provide any functional relevance to the findings. The number of references to supplemental figures far out weighs those to primary figures, again risking the loss of interest in the confused reader."

Response: In the original manuscript we included a lot of detailed information to convey that the detection of synaptoids is reliable and that they resemble typical synapses in many ways. However, we acknowledge that the abundance of information, primarily in the Supplement, might overwhelm readers. Therefore, we have now omitted some findings that were of minor relevance. Specifically, we have removed panels n – u of former Extended data Fig. 2, which showed no difference in the area, volume, or sphericity of synaptoids compared to synapses.

Reviewer #1: "6) Related to the above, the reader is not provided any concept of how the synaptoids on the tanycytes could regulate LH release until the Discussion and then only vaguely so, with mention of the movement of the endfeet. Again most readers will not immediately grasp the idea that the Ca++ changes could be having such an effect nor how the endfeet are regulating the release of GnRH, not LH directly."

Response: We would like to thank the reviewer for pointing out the absence of this important information in the original manuscript. In the revised manuscript, we have presented this concept in

the introduction (page 3, line 22 – page4, line 4). This change will undoubtedly make the text more readable.

Reviewer #1: "7) The statistical tests used, in the limited cases in which they are, are not mentioned and no F values or d.f.s provided following ANOVA."
Response: To provide an easier overview, we have summarized all the statistical information in Supplementary Table 5. This table specifies F values, degrees of freedom, and sample sizes. Additionally, we have mentioned the statistical tests used in the figure legends of the revised manuscript.

Reviewer #2: "In this very exciting study, Neve et al. establish that neurons can communicate with tanycytes via a synaptic-like structure and demonstrate the functional significance of this communication. This is a fundamental discovery that represents a major advance in our understanding of brain function and brain–body communication.
Using sophisticated genetic approaches (including viral tracing), super-resolution imaging, scRNA-seq, DREADDs, calcium, cAMP, and glutamate imaging, as well as endocrine assays, the authors demonstrate the presence of abundant neuron–tanycyte contacts resembling classical neuronal synapses — "synaptoids." They characterize the neuronal populations that form these contacts with tanycytes and show that synaptoids play essential roles in regulating kisspeptin's effects on LH release and pulsatility, as well as on reproductive functon(e.g., the estrous cycle).
There is an overwhelming amount of novel and exciting data presented, the methodology is elegant, and the conclusions are paradigm-shifting. There are only few minor suggestions:"
Response: We thank the reviewer for the positive evaluation.

Reviewer #2 "1. The sample size is not always clearly indicated. For example, for fig 1c – scRNAseq, how many samples (mice) were analyzed? I couldn't find this information in supplemental tables or the method section."
Response: We apologize for the missing information in the original manuscript. The sample size used in Fig. 1c has been specified in the Methods section (scRNAseq data processing); we refer to this section in Supplementary Table 5. Additionally, we have depicted the sample size in individual graphs whenever possible (Fig. 2c; Fig. 4 f, g). If the sample size could not be included in the graph, it is noted in the figure legend. All sample sizes are also listed in Supplementary Table 5.

Reviewer #2 "For figure 1j showing calcium imaging, based on the extended table 4 it seems like it was done in one mouse, is that the case? If so, the sample size should be increased."
Response: We apologize for the unclear presentation of the sample size. The experiments depicted in Fig. 1j were performed on 8 mice, as mentioned in the text (page 8, line 11). We have included the sample size in Supplementary Table 5. Supplementary Table 4 refers to the genotype of the mice, as well as the viral vector and dose.

Reviewer #2 "Figure 3c includes 2 females and 1 male. It would be stronger to have a larger sample size to enable comparison between sexes."
Response: We assume that this comment refers to Fig. 2c. In response to the reviewer's comment, we have repeated the experiment with more mice and can now compare results between sexes.

Moreover, to support the comparison between sexes, we have performed the RbV tracing starting from *Vcan*-targeted tanycytes in additional mice. The distribution of connected neurons was strikingly similar between sexes in both experiments (Supplementary Fig. 4). While some differences between the sexes were apparent, they did not reach statistical significance due to the limited number of mice and the need to correct *p* values for multiple testing given the numerous brain areas in which retrogradely labeled neurons were found.

Reviewer #2 "Fig 3d-m – based on the table, it seems that the data represent 2 animals. The legend states that the data were from 5 mice, but it's unclear if every agonist was tested in 5 mice? If not, the sample size seems too low. Also, quantification of these effects would be helpful."
Response: We apologize for the unclear presentation of the sample size. Each stimulus was investigated using slices derived from at least 4 mice. This information is provided in Supplementary Table 5. In response to the reviewer's comment, we quantified $[Ca^{2+}]_i$ as maximal F/F_0 in all experiments in the new Fig. 3i-p, in which we depict the sample size (number of mice).

Reviewer #2 "Same concerns for fig 4f and g, is there sex differences? For 4h, what's the n, are there sex differences, can the number of traced neurons be quantified?"
Response: We appreciate the comment and have repeated the experiments on more mice. This has allowed us to compare the number of retrogradely traced neurons connected to *Vcan*-targeted tanycytes between the sexes. The number of traced neurons shown in Fig. 4h is quantified in Fig. 4g. There was no difference between the sexes (Supplementary Fig. 4e-g).

Reviewer #2 "2. The ex vivo experiments involving neuropeptide application were conducted using concentrations that would not be expected under physiological condition. For example, 1 mM of Kp10 was applied to brain slices, whereas only 1 nmol per 25g mouse (more than 100-fold lower) was used to stimulate LH secretion in vivo. Similar discrepancies apply to other neuropeptides used in the study. The authors should discuss a rationale for the doses used—for instance, if such high concentrations could be reached locally near the release site (e.g., within the synaptic cleft). If that is not the case, it would be important to discuss tanycyte-mediated effects of these peptides under physiological conditions."
Response: We are grateful to the reviewer for bringing up this important topic. As the reviewer correctly stated, we applied high concentrations of stimuli due to the thickness of the slices (180 µm) and the short stimulation period of 10 seconds. This protocol supports tanycyte viability and enabled us to evaluate the time course of $[Ca^{2+}]_i$ or $[cAMP]_i$. While the concentrations in the perfusate in this protocol may not reflect those at the tanycyte membrane, we believe the stimulation was specific because the effect of the stimuli was concentration-dependent, as demonstrated with carbachol (Supplementary Fig. 8d), and could be blocked by receptor antagonists. The cholinergic antagonists atropine and MT7 inhibited stimulation of $[Ca^{2+}]_i$ by carbachol (Supplementary Fig. 8e, f), confirming that the effect was mediated by cholinergic M1 receptors. Furthermore, while revising the manuscript, we tested the effect of the kisspeptin receptor 1 antagonist Kp234 (Chen et al., 2025) and found that it inhibited $[Ca^{2+}]_i$ stimulated by Kisspeptin-10 (Fig. 3f-h). Previously, when we applied this protocol, we observed that the effect of the TRH analogue taltirelin was lost in TRH receptor 1 knockout slices, but not in TRH receptor 2 knockouts (Müller-Fielitz et al., 2017). Therefore, we conclude that the effects are mediated by typical membrane receptors for the various agonists and that the high concentrations are due to technical details of the experimental model, not reflecting

receptor affinity. These considerations have been included in the method section, "Measurement of tanycytic cAMP and Ca^{2+} levels in acute brain slices" (page 30, lines 18-20).

Reviewer #3: "Summary:
The study by Nava and colleagues characterized the heterogeneity of neuronal and the tanycytic populations by studying the synaptoid connectome between these two populations. The researchers examined this synaptoid connectivity by utilizing high-resolution microscopy to identify post-synaptic proteins residing in tanycytes, rabies monosynaptic retrograde tracing to identify the unique neuronal inputs to subpopulations of tanycytes, Ca2+ imaging to functionally confirm tanycyte responsiveness to various neurotransmitters and neuropeptides, and genetic screening to confirm tanycytic transcriptional heterogeneity. They further investigate the Kiss1 neuron population and its connection to tanycytes in the context of reproductive function. The authors propose subpopulations of tanycytes may differentially regulate the effects of kisspeptin on downstream LH release patterns. Much of this work is carefully done and detailed that considerably expands our understanding of the extent to which glia are integrated in neuronal networks and their contribution to physiological behavior. Some of the stronger statements about the control of LH release will likely require additional studies and/or explanation:"
Response: We appreciate the positive evaluation and hope that that the additional data we have provided to support our conclusions meet the reviewer's expectations.

Reviewer #3: "Comments: 1) The authors make the criteria for distinguishing between β and α tanycytes clear, but what were the criteria for distinguishing β1 from β2 (and α1 from α2) tanycytes in Extended data Fig. 1c-h? It will be useful to explicitly state these criteria given the conclusions about some of the effects on reproduction being specific to β2 tanycytes."
Response: The distinction between different tanycyte subpopulations was based on their location along the 3rd ventricle and proximity to hypothalamic nuclei or regions (DMH, VMH, ARC, and median eminence). In particular, β2-tanycytes are characterized by their projections into the median eminence. This classification has also been used by others (e.g., Langlet, 2019). We have defined the criteria for tanycyte subpopulations in the revised manuscript (page 38, lines 1-3).

Reviewer #3: "2) The authors suggest that β2 regulate basal LH release based on the different effects of DIO::shKiss1R and Vcan::shKiss1R. What is meant by basal LH release (also mentioned in abstract and discussion)? Odd term to describe a pulsatile system."
Response: The term "basal LH levels" does not really contradict the notion of pulsatile secretion, since the pulses are superimposed on a persistent low, i.e. basal, level of LH. Indeed, the term is firmly established in the literature on LH release in mice and has a clear definition. It has been used by Czieselsky et al (2016) and other groups (Kreisman et al., 2022; Patel et al., 2023; Talbi et al., 2025). Basal LH levels were determined as described in the revised manuscript (page 40, lines 5-18).

Reviewer #3: "Is there a specific promoter for β2 tanycytes to directly assess effects on this mode of LH?"
Response: The *A2m* promoter is specific for β1+β2 tanycytes. In response to the reviewer's question, we have complemented the subpopulation-specific knockdown of *Kiss1r* by using the *AAV-A2m-Cre* vector to target the β1+β2-tanycytes. As expected, the basal LH release was reduced in female

tanycyte$^{A2m::shKiss2R}$ mice (Fig. 5q). In contrast, the basal LH release increased in male tanycyte$^{A2m::shKiss2R}$ mice (Supplementary Fig. 13ab). The cause of this sexual dimorphism is still unclear. We hypothesize that it may be related to sexually dimorphic kisspeptin input from the AVPV to ventral β1-tanycytes, which are the one ensheathing GnRH neuronal terminals and controlling GnRH release. Females have significantly more Kiss1 neurons in the AVPV than males of different species, including mice, rats, and humans (Clarkson and Herbison, 2006; Hrabovszky et al., 2010; Kauffman et al., 2007). Consistent with the idea that Kiss1 neurons in the AVPV stimulate basal LH release, especially in females, knockdown of *Kiss1r* in tanycytes reduced basal LH levels in female mice.

Reviewer #3: "3) Given that the viruses are injected into the lateral ventricles, have the authors checked for "off target" expression of these novel AAVs across all ventricular space (e.g., subventricular zone)?"
Response: We appreciate this important question. We have imaged sections of the whole brain for each of the novel vectors. Occasionally, transduction occurred in the ependymal lining of other ventricles, but we did not observe off-target reporter gene expression in the parenchyma or the subventricular zone in any case. In the revised manuscript, we have included representative images of the subventricular zone (Supplementary Fig. 10).

Reviewer #3: "4) Colocalization of some markers with Rbv (e.g., kiss, VGAT AVP ChAT TH) is clear, but other markers are difficult to visualize as double labeled (Vglut) as there is a general background fluorescence in this channel. Adding DAPI or other nuclear marker might help with this. Extended data Fig. 4e, f, and g, it is difficult to visualize and confirm coexpression of the makers when only the merged channels are shown. Green arrow in ext data 4 is not defined."
Response: Following the reviewer's suggestion, we have added the DAPI channel to images in Supplementary Fig. 5e, f, and g (former Extended data Fig. 4e, f, and g) to improve the image quality. Additionally, we have made the arrows in Supplementary Fig. 5 the same color (yellow).

Reviewer #3: "5) What was the methodology for calculating the ΔF/F0 for the calcium imaging experiments? E.g., how was bleaching corrected, how was baseline determined, was any post-hoc smoothing done?"
Response: We agree with the reviewer that the description of the methodology for these experiments in the original manuscript was too brief. We have revised the Methods section describing the stimulation of slices and the quantification of fluorescence ('Measurement of tanycytic cAMP and Ca^{2+} levels in acute brain slices', pages 29-30). In response to the reviewer's specific question, the baseline was recorded for one min prior to stimulation. The fluorescence was so stable that post-smoothing was unnecessary. Bleaching was also not an issue during the short-term fluorescence recording because we only stimulated primary tanycytes or slices 3-times. If bleaching had occurred, it would have reduced the F/F_0 ratio, where F_0 is determined before F, but we found an increase in most cases.

Reviewer #3: "6) Fura-2-AM is mentioned in the methods for the Ca2+ level detection. Fluo4 is also mentioned in Extended data Fig. 7. However, the bulk of the data appear to be with AAV-CAG-GCaMP6s . What is the rationale for using all of these calcium sensor indicators? There do not appear to be any studies included with Fura2.

Response: We used Fluo4 in primary tanycytes to measure intracellular Ca^{2+} because these cells are difficult to transfect. To measure Ca^{2+} levels in brain slices with high image quality, we transduced tanycytes with a GCamP6s vector. The only exception was that we measured Ca^{2+} levels in brain slices of $G\alpha_{q/11}^{gliaKO}$ and control mice using Fura2. We have reworded the Methods section to emphasize that Fura2 was only used in this experiment (page 30, line 21).

Reviewer #3: "7) The use of AAV to deliver specific promoter-driven cres does a nice job of distinguishing different populations of tanycytes. Activation of hM4Di blunted LH release when Dio and A2m cells, but not Vcan cells, were targeted. Interesting finding but the effect is mild. The authors need to discuss the different timing of the peak in LH between the DIo2 and A2m mice and also the increase in LH in the CNO treated mice at the final timepoint, is there a different physiological effect at a delay? These data should be on the same scale to facilitate comparison. The CNO-induced LH release from A2m::hM4Di mice in Fig. 5c is dramatically lower and has different kinetics than in 5a and 5b (not to mention the differences in kinetics between 5a and 5b, especially the LH rise in the hM4Dq group). Is it possible other non-tanycytic populations along the ventricles are contributing to these differences in LH patterns? If not, what other explanations are there for these differences, especially between the saline + Kp10 mice across 5a, b, and c? Perhaps there is CNO-independent activation of the hM4Di receptors?"

Response: The reviewer correctly notes that the time course of LH release varies slightly between experiments. Unfortunately, we can only speculate about the reasons for this variation, which may include differences in Kp10 lots or LH ELISA batches. It is important to note that the two groups (saline + Kp10 vs. CNO + Kp10) in experiments Fig. 5a, 5b, and 5c were performed in the same mice with a cross-over design. The order of the treatment was randomly assigned (page 39, lines 9-16). Therefore, comparing the two treatment groups is meaningful and should not be confounded by variability in the time course. For technical reasons, we had to test the three viral vectors (*AAV-Dio2-Cre*, *AAV-Vcan-Cre-2A-GFP*, and *AAV-A2m-Cre-2A-GFP*) in separate animal cohorts and experiments, which likely explains the variability in the time course. In other cases as well, the time to peak and LH amplitude in response to Kp10 varied between experiments in female mice. Importantly, we determined that non-tanycytic cell populations along the ventricles did not contribute to LH release by monitoring transduction specificity with the mCitrine reporter in *hM4Di* mice. These results are included in Supplementary Fig. 12a. Furthermore, CNO itself did not affect LH release in *hM4Di* mice without Cre-mediated recombination and Kp10 treatment in 5 mice. These data are shown in Supplementary Fig. 12b. In the legend to Fig. 5a-c, we explicitly state that experiments in Fig. 5a, b and Fig. 5c were not performed in parallel and that y axis differ between panels.

Reviewer #3: "8) Blood sampling was performed during estrus for female mice. The patterns observed are substantially different than published work, which report 1 pulse/4h for LH (doi: 10.1210/en.2016-1351) or 1 calcium event per 10h in vivo for arc kiss neurons (doi: 10.1210/en.2019-00193). Information on assay sensitivity and pulse detection algorithms must be provided (the decay phase of some pulses is quite abrupt 5i, 5n) and at minimum an explanation provided for the different results."

Response: We thank the reviewer for addressing the method of pulse detection. The reviewer is correct that there is significant biological variability in pulse frequency, particularly during estrus. The reviewer cites two papers, one of which reports a frequency of 1 pulse/4 h (Czieselsky et al., 2016, i.e., DOI: 10.1210/en.2016-1351). This frequency is below the approximately 0.5 pulses/h that we observed (Fig. 5f, k, p). The other report, however, (McQuillan et al., 2019, i.e., doi:

10.1210/en.2019-00193) describes a broad range of frequencies, from 0.4 ± 0.1 /h (Fig. 1A-E of McQuillan et al., 2019) to a low frequency of 1 pulse/10 h. We contend that our determined frequency fits the upper range of frequencies in the McQuillan et al. (2019) paper. The variability in LH pulse frequencies reported between studies may be caused by subtle differences in how the mice were handled, when the investigation was conducted, or the animals' genetic background. Importantly, the frequencies in Fig. 5 align well with our observations in other cases. In the revised manuscript, we provide a detailed description of pulse detection and assay sensitivity (page 40, lines 3 onwards; page 39, lines 22-23).

Reviewer 3: "8) It is also puzzling why A2m targeted cells were not included given that is what was mediating effects in diestrus and that these cells are closer to GnRH terminals in the median eminence. Ideally, the studies should be repeated on diestrus and A2m-targeted tanycytes included because the statement on line 359 that "α+β1 tanycytes exert a Kiss1-mediated slowdown of GnRH pulsatility" requires rigorous support as kisspeptin has been viewed as an activator in this system, as do the statements on lines 430-435 of the discussion."

Response: In response to the reviewer's comment, we investigated LH release in tanycytes$^{A2m::shKiss1R}$ mice while revising the manuscript. For these new experiments, we selected estrus because previous experiments in tanycytes$^{Dio2::shKiss1R}$ and tanycytes$^{Vcan::shKiss1R}$ mice (Fig. 5d-m) were also performed during this stage. The new data confirmed that *Kiss1r* knockdown in *A2m*-targeted β1+β2-tanycytes decreased basal LH release in female mice. Concurrently, it increased LH pulse frequency, similar to the effect observed with *Kiss1r* knockdown in Vcan- and Dio2-targeted tanycytes. This suggests that a population of tanycytes targeted by all three vectors (i.e., dorsal β1-tanycytes) modulates frequency in a kisspeptin-dependent manner. In males, *Kiss1r* knockdown in *A2m*-targeted β-tanycytes reproduced the effect on LH pulse frequency observed in females (Supplementary Fig. 13aa). However, surprisingly, *Kiss1r* knockdown in *A2m*-targeted β-tanycytes had the opposite effect on basal LH levels in males than in females (Supplementary Fig. 13ab). The causes of this sexual dimorphism are unclear, but we speculate that Kiss1 neurons in the AVPV have a smaller impact on β-tanycytes in male mice due to their lower number (Clarkson and Herbison, 2006). Thus, the indirect effects of *Kiss1r* knockdown and increased LH frequency may have a stronger impact on basal LH levels in males. Indirect effects could include the negative feedback by steroid hormones. The reviewer also suggested that ideally all experiments should be repeated in diestrus, probably because we had selected this stage for the Kp10 stimulation of LH release in the experiments shown in Fig. 5a-c. However, when it comes to investigating the pulse frequency, we are concerned that the higher pulse frequency in diestrus may conceal the accelerating effect of the *Kiss1r* knockdown in tanycytes. Therefore, we doubt that a repetition of experiments in diestrus will be rewarding. Furthermore, we would not be able to complete the extensive experimentation, including 3 viral treatment groups, in a timely manner.

Reviewer #3: "9) The term LH baseline is used in Fig. 5, how is this calculated? Is this perhaps interpulse nadir (baseline like basal an odd term for a pulsatile system). Reporting LH data as AUC (ext data 10) is not helpful as both frequency and amplitude information are eliminated and these are critical elements for assessing neuroendocrine activity."

Response: We thank the reviewer for raising this point. In the revised manuscript, we included a detailed description of how the basal LH levels were determined (page 40, lines 12-14). We agree with the reviewer that the AUC, which depends on both frequency and amplitude, would not be

sufficient as the only readout. However, we included the AUC in addition to frequency and amplitude to remain consistent with several other studies in this field.

Reviewer #3: "10) Cycle success is quite variable in controls- 50% in one study and 75% in another, suggesting cycles in these mice are already poor. Were these mice verified to be cycling before treatment. The more traditional method of evaluating % days spent per stage does not reveal a difference, although % data should not be compared with t test, more appropriate to use chi square or Fisher exact."

Response: The mice's imperfect cyclicity is likely caused by the stereotactic surgery performed for the AAV vector injection. In response to the reviewer's comment, we have presented the results in days per stage rather than as a percentage**.**

Reviewer 3: "Other points
L77-78 it is an overstatement that the connection of tanycytes to specific neurons is unique among glia cells as not all glial populations have been investigated"

Response: We agree with the reviewer that the wording may have been too strong in the original manuscript and have reworded the text to take this aspect into consideration (page 4, lines 4-5).

Reviewer 3: "L118, citation missing"

Response: In response to reviewer 1, we have removed the comparison of synaptoids with classical synapses from the text.

Reviewer 3: "L141 TTX attenuated not prevented"

Response: We have corrected the text according to the reviewer's suggestion (page 8, line 12).

Reviewer 3: "L143 response is not dependent on TTX but on what TTX blocks"

Response: We thank the reviewer for pointing out this mistake. The text has been corrected accordingly (page 8, line 15).

Reviewer 3: "L379-381 The view that glia are no integral components of neural networks is outdated. For example, the tripartite synapse emerged as a concept about 30 years ago."

Response: In line with the reviewer, we acknowledge the important role of tripartite synapses repeatedly in the manuscript (e.g., Introduction, page 3, lines 4-6 where it says "astrocytes… stabilize or prune neuron-neuron synapses" and first paragraph of the discussion page 19). Here we intended to stress that tanycytes are not simply involved in synapses in a manner similar to astrocytes, but that they themselves are the post-synaptoid target. The text has been reworded to avoid giving the wrong impression (page 21, lines 5-7).

Reviewer 3: "L382 the data clearly demonstrate very interesting projections and interactions for tanycytes with glia but other types of glia have also been shown to functionally interact with neurons, so unusual is not an accurate term."

Response: We have followed the reviewer's suggestion and revised the wording to "… tanycytes are an exception …" (page 21, line 5).

References

Chen, X., S. Yang, N.D. Shaw, and M. Xia. 2025. Kisspeptin Receptor Agonists and Antagonists: Strategies for Discovery and Implications for Human Health and Disease. *International Journal of Molecular Sciences* 26:4890.

Clarkson, J., and A.E. Herbison. 2006. Postnatal Development of Kisspeptin Neurons in Mouse Hypothalamus; Sexual Dimorphism and Projections to Gonadotropin-Releasing Hormone Neurons. *Endocrinology* 147:5817–5825.

Czieselsky, K., M. Prescott, R. Porteous, P. Campos, J. Clarkson, F.J. Steyn, R.E. Campbell, and A.E. Herbison. 2016. Pulse and Surge Profiles of Luteinizing Hormone Secretion in the Mouse. *Endocrinology* 157:4794–4802.

Hrabovszky, E., P. Ciofi, B. Vida, M.C. Horvath, E. Keller, A. Caraty, S.R. Bloom, M.A. Ghatei, W.S. Dhillo, Z. Liposits, and I. Kallo. 2010. The kisspeptin system of the human hypothalamus: sexual dimorphism and relationship with gonadotropin-releasing hormone and neurokinin B neurons. *European Journal of Neuroscience* 31:1984–1998.

Kauffman, A.S., M.L. Gottsch, J. Roa, A.C. Byquist, A. Crown, D.K. Clifton, G.E. Hoffman, R.A. Steiner, and M. Tena-Sempere. 2007. Sexual differentiation of Kiss1 gene expression in the brain of the rat. *Endocrinology* 148:1774–1783.

Kreisman, M.J., R.B. McCosh, and K.M. Breen. 2022. A Modified Ultra-Sensitive ELISA for Measurement of LH in Mice. *Endocrinology* 163:

Langlet, F. 2019. Tanycyte Gene Expression Dynamics in the Regulation of Energy Homeostasis. *Frontiers in endocrinology* 10:

McQuillan, H.J., S.Y. Han, I. Cheong, and A.E. Herbison. 2019. GnRH Pulse Generator Activity Across the Estrous Cycle of Female Mice. *Endocrinology* 160:1480–1491.

Müller-Fielitz, H., M. Stahr, M. Bernau, M. Richter, S. Abele, V. Krajka, A. Benzin, J. Wenzel, K. Kalies, J. Mittag, H. Heuer, S. Offermanns, and M. Schwaninger. 2017. Tanycytes control the hormonal output of the hypothalamic-pituitary-thyroid axis. *Nat Commun* 8:484.

Patel, B., K. Koysombat, E.G. Mills, J. Tsoutsouki, A.N. Comninos, A. Abbara, and W.S. Dhillo. 2023. The emerging therapeutic potential of kisspeptin and neurokinin B. *Endocrine Reviews*

Talbi, R., T.L. Stincic, K. Ferrari, C. Ji Hae, K. Walec, E. Medve, A. Gerutshang, S. Leon, E.A. McCarthy, O.K. Rønnekleiv, M.J. Kelly, and V.M. Navarro. 2025. POMC neurons control fertility through differential signaling of MC4R in kisspeptin neurons. *eLife* 13:RP100722.

Reviewer #3:
The authors have included new data, analyses and images and rewritten aspects of the manuscript. These result in a markedly improved presentation of this work, which is important and impactful. The addition of the sex comparisons is notable, but the authors should indicate they've chosen to compare the postovulatory state in females (estrus) to males, rather than an arguably less sexually differentiated state. Future work examining other cycle stages and sex differences for these interesting findings would be welcome.

Response: With respect to the reviewer's comment, we explicitly state that we have analyzed LH pulsatility during estrus (page 18, line 21; page 39, line 12).
