# Supplementary material for: A synaptoid connectome differentiates tanycytic subpopulations and underlies neuroglial communication and neuroendocrine regulation
Source: Nat Commun. 2026 Jun 20;17:7797. doi: 10.1038/s41467-026-74598-5 (PMC13439102; doi:10.1038/s41467-026-74598-5)
Supplement: Supplementary file 3 [file 41467_2026_74598_MOESM1_ESM.pdf]

Supplementary Information

A Synaptoid Connectome Differentiates Tanycytic Subpopulations and Underlies Neuroglial Communication and Neuroendocrine Regulation

Vanessa Neve†, Daniela Fernandois†, Surya Rai, Ümit Özorhan, Sreekala Nampoothiri, Gaetan Ternier, Spyridon Sideromenos, Sarah Gallet, Cecile Allet, Marcus Stahr, Natascha Klaus, Ines Martinez-Corral, Caio Fernando Ferreira Coêlho, Akila Chandrasekar, Andreea Constantinescu, Manon Rivagorda, Simon Dührkop, Julia Cases Bazarra, Sonja Binder, Paolo Giacobini, Sowmyalakshmi Rasika, Ruben Nogueiras, Tibor Harkany, Martin K. Schwarz, Helge Müller-Fielitz‡, Vincent Prevot*‡, Markus Schwaninger*‡

Corresponding authors: markus.schwaninger@uni-luebeck.de, vincent.prevot@inserm.fr

The PDF file includes:

Supplementary Fig. 1 to 13
References

Other Supplementary Materials for this manuscript include the following:

Supplementary Data file 1 to 5
Supplementary Video 1 to 8

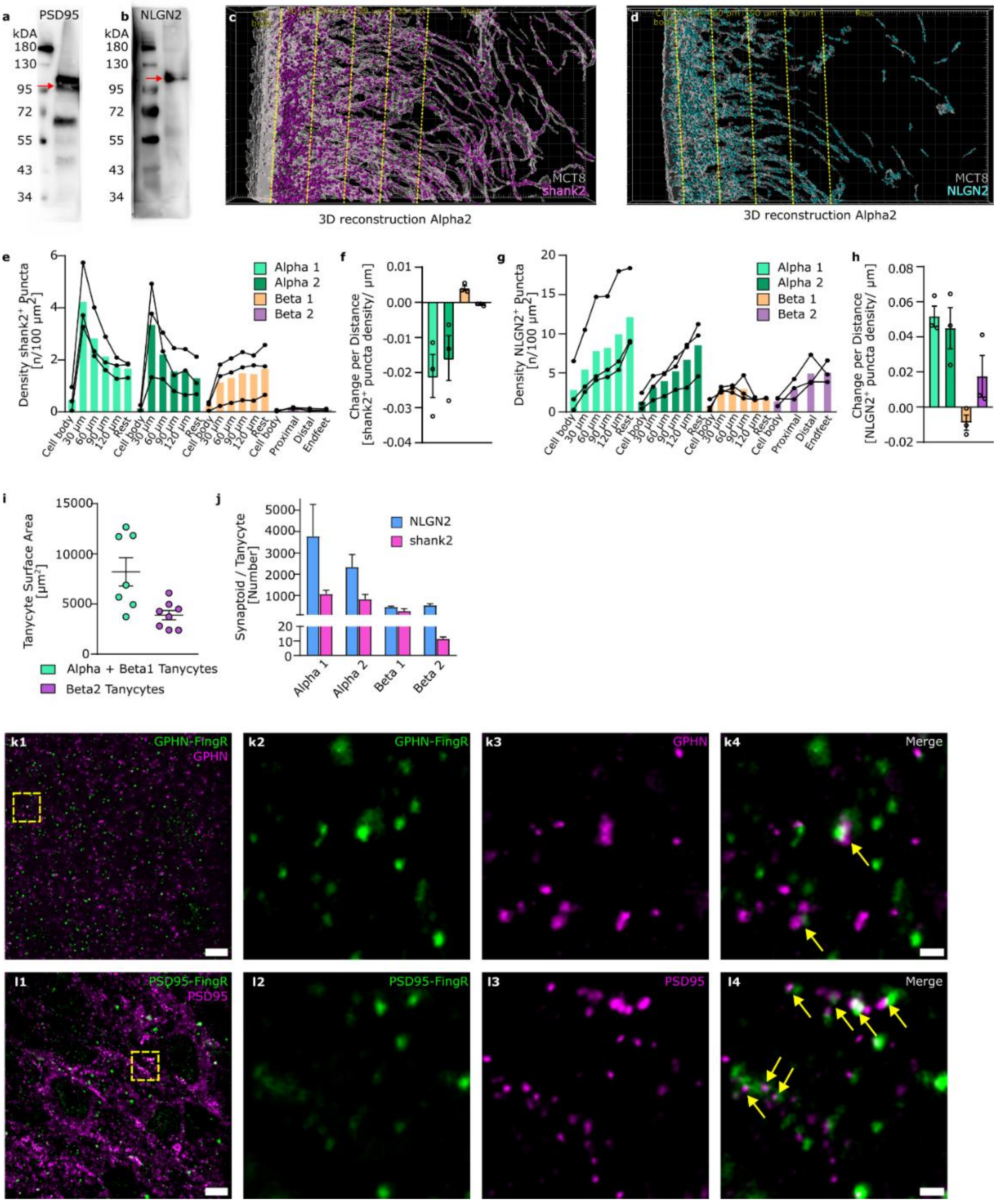

Supplementary Fig. 1. Synaptoid terminals on tanycytes. a, b) PSD95 (95 kDa) and NLGN2 (110-120 kDa) in primary tanycyte cultures as detected by Western blotting. Arrows, specific bands. **c, d)** 3D reconstruction of α2 tanycyte membrane using MCT8 (grey) and colocalizing with either SHANK2 (pink, **c**) or NLGN2 puncta (cyan, **d**). Yellow lines mark 30-µm zones in which SHANK2 and NLGN2 puncta were counted. **e-h)** Density of SHANK2 (**e, f**) or NLGN2 (**g, h**) puncta relative to the distance from the ventricle wall in the different tanycyte subpopulations. Mean ± SEM. **i)** Estimated surface area of α+β1-tanycytes and β2-tanycytes. After sparsely labelling tanycytes by injecting AAV-Cre into *mT/mG* reporter mice and confocal imaging of mGFP, the surface was reconstructed in 3D (not shown). Mean ± SEM. **j)** Estimated number of NLGN2- and SHANK2-positive puncta per tanycyte shown as mean + SEM based on estimated tanycyte surface areas. **k, l)** *GlastCreER*^{T2} mice were injected with *AAV-EF1a-flex-Gphn-FingR-GFP* (**k**) or *AAV-EF1a-flex-PSD95-FingR-GFP* (**l**) to label endogenous tanycytic postsynaptic proteins. Co-staining with GPHN or PSD95 confirmed the correct labelling of endogenous proteins. Overviews (**k1, i1**) were imaged by confocal microscopy; scale bars 5 µm. Magnified images (**k2, 3; i2,**

3) were imaged using 2D-STED microscopy; scale bars, 0.5 µm. Detailed information on the sex of the animals is provided in Supplementary Data file 5.

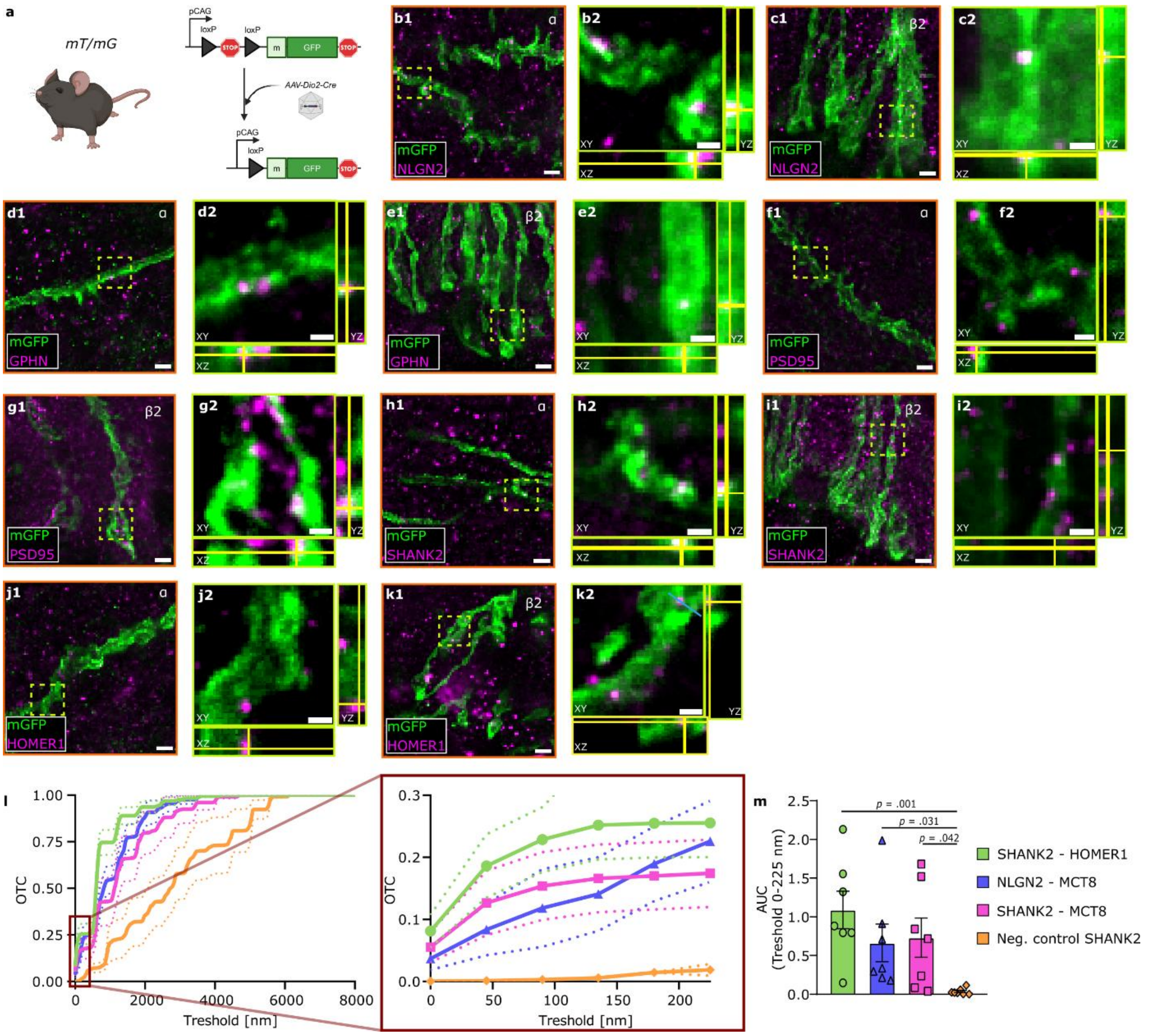

Supplementary Fig. 2. STED analysis of synaptoids. a) Scheme of *mT/mG* mouse model to express membrane bound GFP after Cre-recombinase induction. Created in BioRender. Schwaninger, M. (2026) https://BioRender.com/2o35jyv. **b-k)** Post-synaptic proteins are localized in the tanycytic plasma membrane. After sparsely labelling tanycytes by injecting AAV-Cre into *mT/mG* reporter mice, mGFP was detected by confocal microscopy and NLGN2 (**b, c**), GPHN (**d, e**), PSD95 (**f, g**), SHANK2 (**h, i**) and HOMER1 (**j, k**) with 3D-STED super-resolution microscopy in α1-tanycyte process (**b1**, **d1, f1, h1, and j1**) and β2-tancyte process (**c1**, **e1**, **g1, i1** and **k1**). Scale bars, 2 µm. **b2 – k2**) High magnification of the boxed areas in **b1-k1** with orthogonal views (YZ and XZ views). Scale bars, 0.5 µm. **l, m)** Optimal transport colocalization (OTC) analysis showed spatial proximity between synaptoids and tanycytic plasma membranes in the range that is characteristic for super-resolution microscopy. The fraction of pixel intensities that were matched (colocalized) between NLGN2 or SHANK2 and MCT8 as marker of the tanycyte plasma membrane were plotted. The post-synaptic proteins SHANK2 and HOMER1 served as colocalized control. Area under the curve for the threshold of 0 – 225 nm was used for statistical analysis (**m**). Means ± SEM are shown, *N* denotes the number of ROIs. Two-tailed Kruskal-Wallis followed by Dunn´s post hoc tests. Detailed information on the test statistics, sex and number of animals is provided in Supplementary Data file 5.

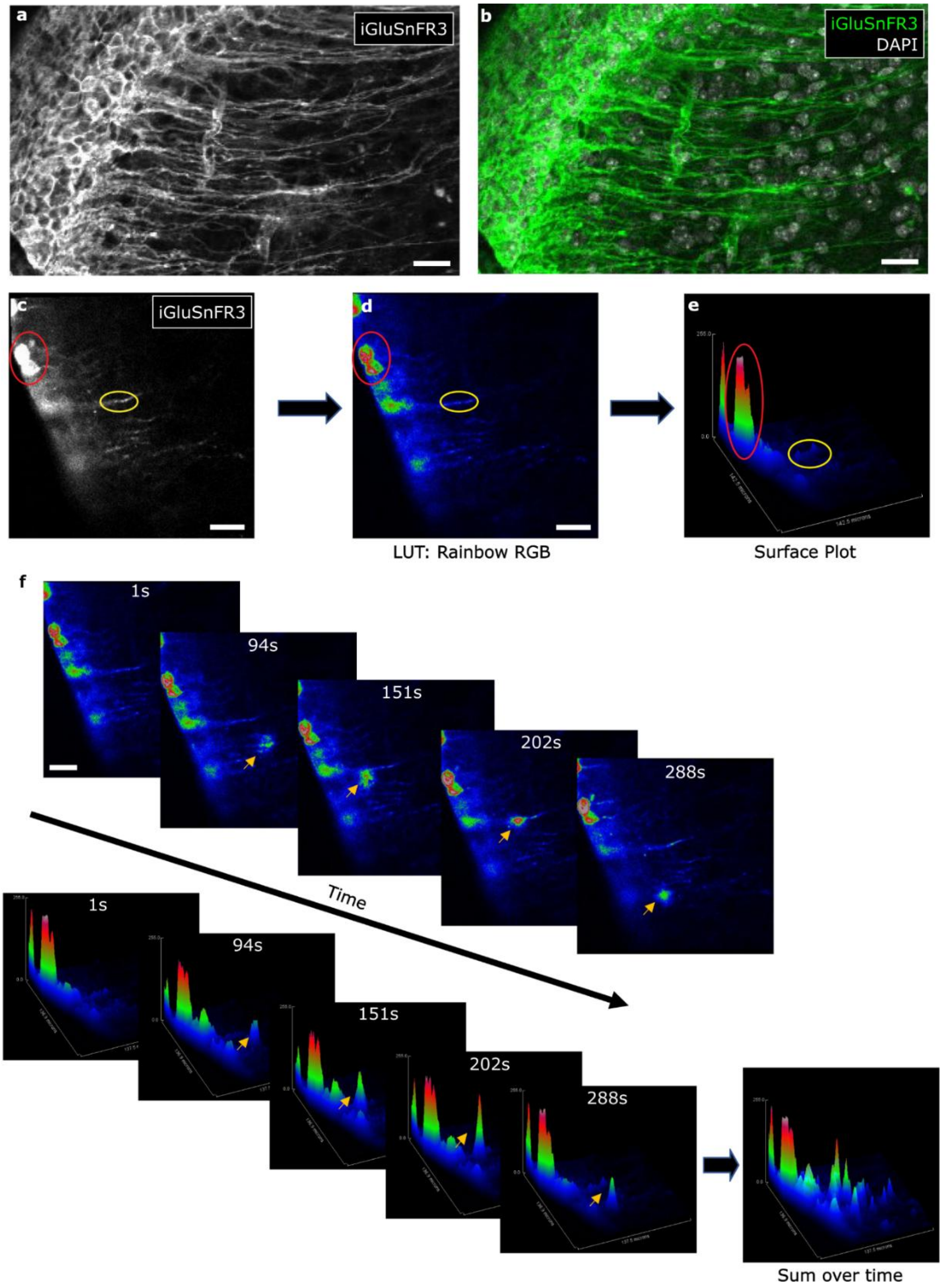

Supplementary Fig. 3. Imaging glutamate release in tanycyte synaptoids. a–b) Two weeks after transducing tanycytes *in vivo* with AAV-CAG-iGluSnFR3, the glutamate sensor was selectively detected in the plasma membrane of tanycytes. After intravital imaging, the slices were fixed in 4% PFA and immunostained to detect the sensor distribution along the tanycytes (green). **c–f)** After expressing iGluSnFR3 in tanycytes, glutamate imaging was carried out in acute coronal brain slices. Fluorescence images (**c**) were converted to a false color scale (FIJI, LUT Rainbow RGB, **d**) and further transformed into a surface plot (FIJI, **e, f**), displaying the local fluorescence intensity of x/y images in a third

dimension. To better visualize the fast (<1 seconds) and localized glutamate release after the bath application of KCl (30 mM, 30 seconds), the resulting 3D time stacks (videos 5–7) were summed over time. Scale bars, 20 µm. Representative processing of a replicate of 8 animals.

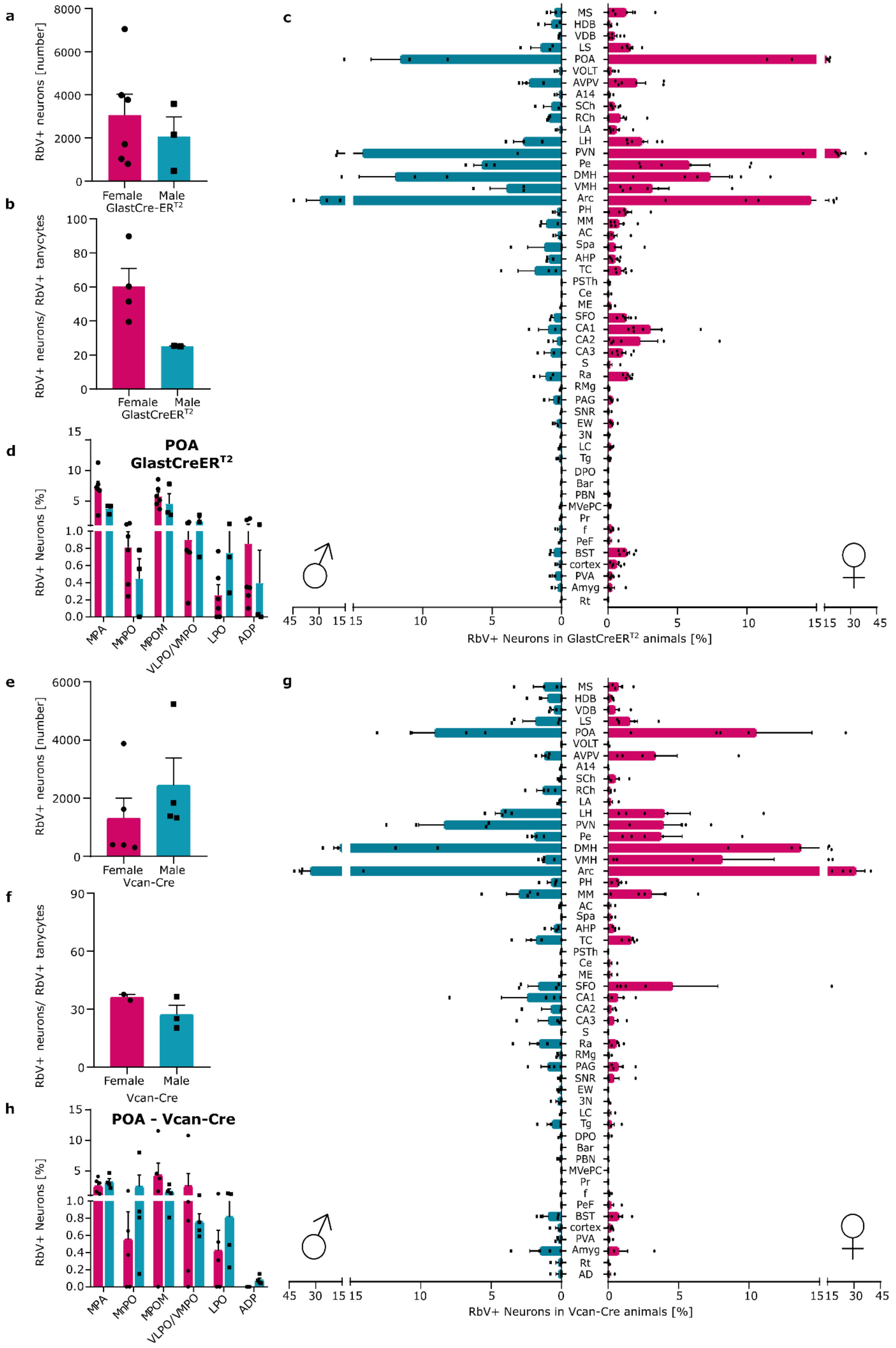

Supplementary Fig. 4. The distribution of retrogradely labelled neurons did not differ between female and male mice. a-d) Analysis of the data shown in Fig. 2 according to the sex of mice. Retrograde tracing was performed in *GlastCreER^{T2} mice*. **a**) Total number of retrogradely traced RFP-

positive neurons in female and male mice. **b**) Ratio of RFP-positive neurons per RFP-positive starter tanycytes in female and male mice. **c**) The distribution of retrogradely traced neurons in the brain regions of male and female mice is expressed as a percentage per mouse. **d**) Distribution of retrogradely traced neurons within nuclei of the preoptic area (POA). **e-h**) Analysis of the data shown in Fig. 4d-f according to the sex of mice. Retrograde tracing starting from *Vcan*-targeted tanycytes. **e**) Total number of retrogradely traced RFP-positive neurons in female and male mice. **f**) Ratio of RFP-positive neurons per RFP-positive starter tanycytes in female and male mice. **g**) The distribution of retrogradely traced neurons in the brain regions of male and female mice is expressed as a percentage per mouse. **h**) Distribution of retrogradely traced neurons within nuclei of the POA. Values are means + SEM, *N* denotes the number of mice. ADP, anterodorsal preoptic nucleus; LPO, lateral preoptic area; MnPO, median preoptic nucleus, MPA, medial preoptic area; MPOM, medial preoptic nucleus, medial part; VLPO/VMPO, ventrolateral/ ventromedial preoptic nucleus. Detailed information on the test statistics, sex and number of animals is provided in Supplementary Data file 5.

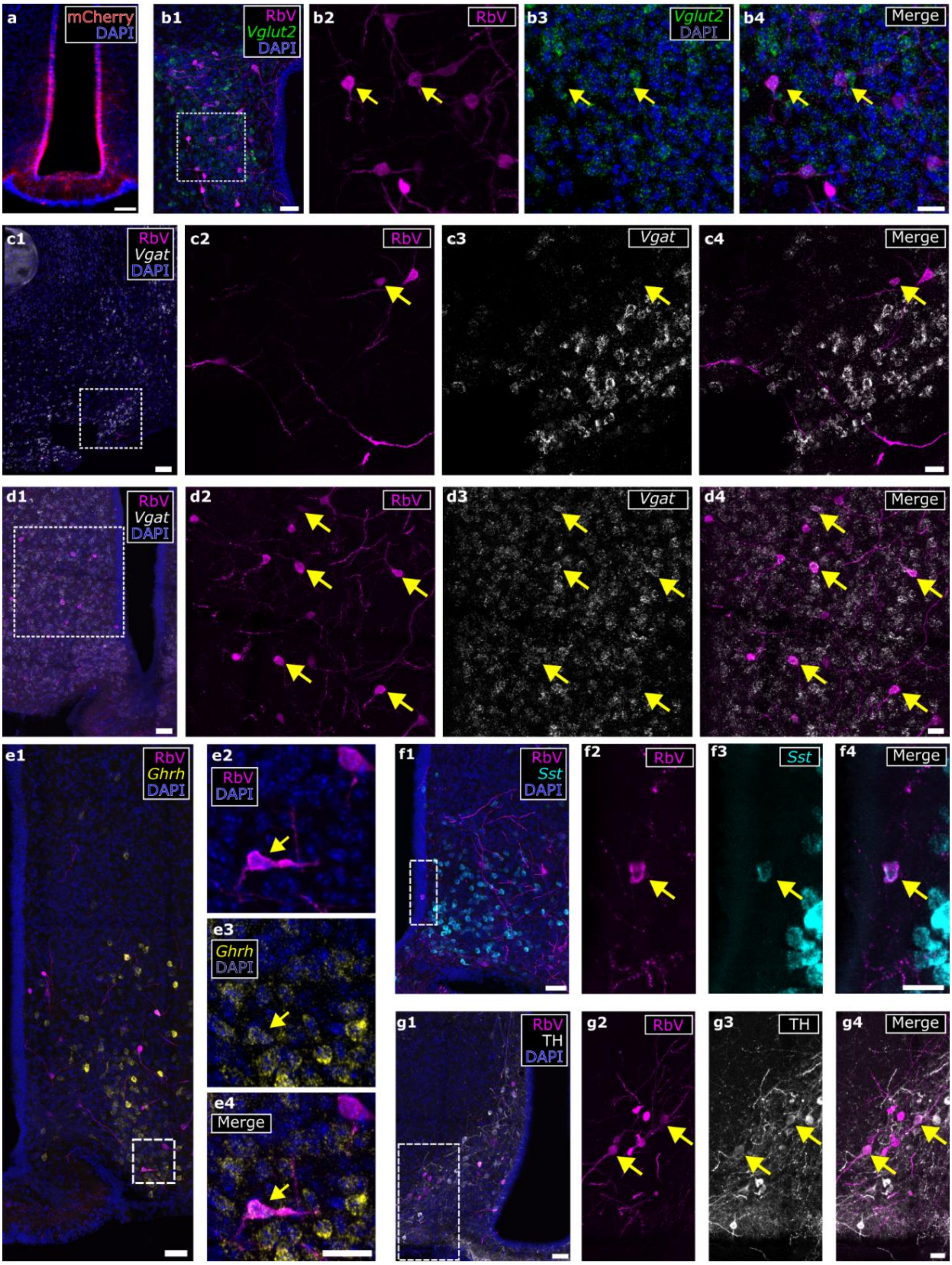

Supplementary Fig. 5 Identification of retrogradely labelled neurons in the hypothalamus. Retrogradely traced RFP-positive neurons (RbV, magenta) were characterized by immunofluorescence staining or *in situ* hybridization. Yellow arrows show co-labelled cells. **a)** mCherry was expressed specifically in tanycytes after injecting *AAV-flex-mCherry* i.c.v. in *GlastCreER*^{T2} mice. Scale bar, 100 µm. **b)** In the paraventricular nucleus (PVN), RbV-positive neurons expressed *Vglut2* (green). Scale bar b1, 50 µm; magnified images (b2-b4), 25 µm. **c, d)** RbV-positive neurons expressed the GABAergic marker *Vgat* in the medial preoptic area (MPA, **c**), the ventromedial hypothalamus (VMH) and the arcuate nucleus (ARC, **d**). Scale bar c1, 100 µm; magnified images, 25 µm. Scale bar d1, 50 µm; magnified images, 25 µm. **e-g**) In the ARC, RbV neurons expressed *Ghrh* (**e**), Sst (**f**) and TH (**g**). Scale

bar e1, f1 and g1, 100 µm; magnified images, 25 µm. Detailed information on the sex of the animals is provided in Supplementary Data file 5.

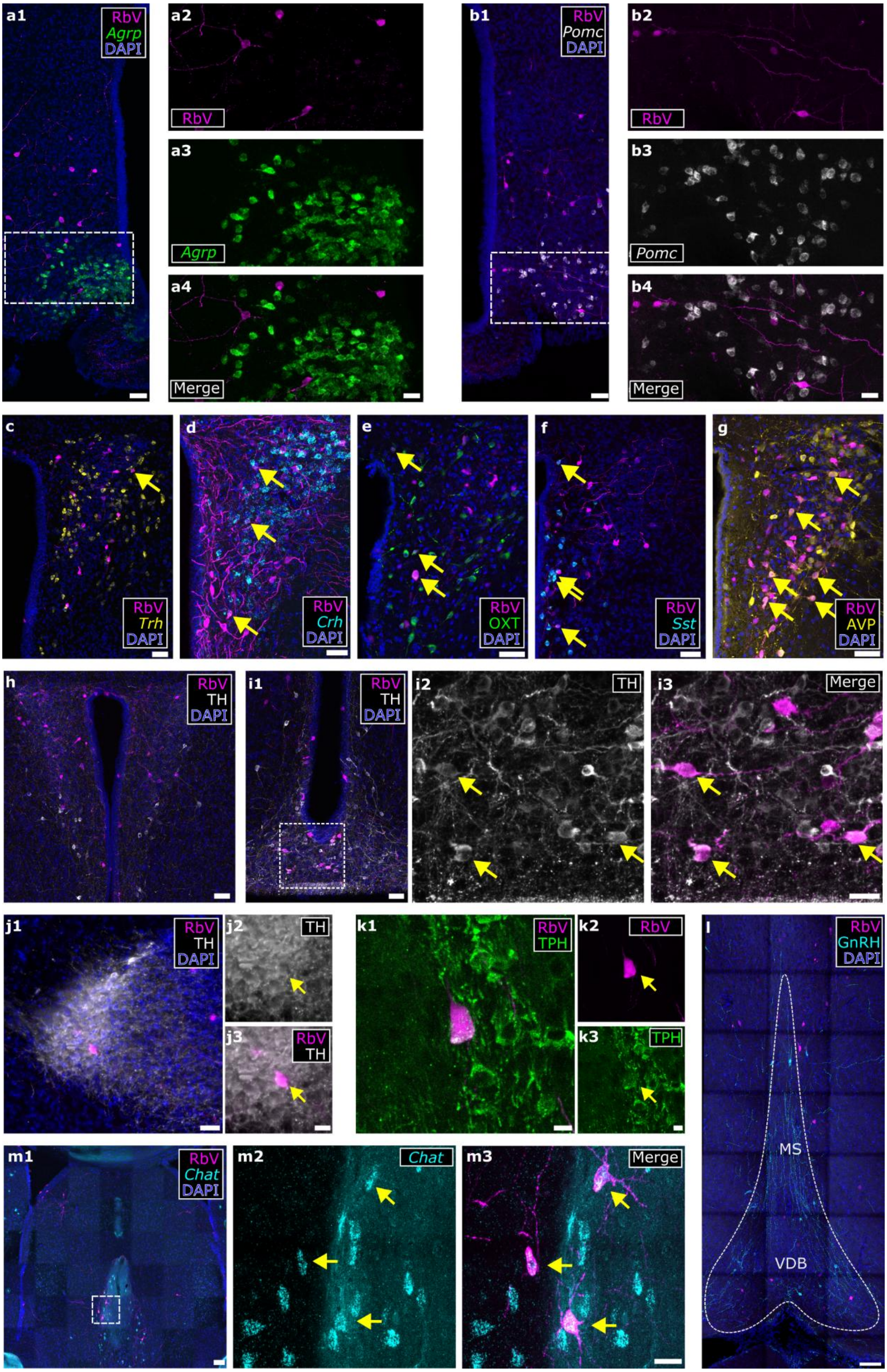

Supplementary Fig. 6. Identification of retrogradely labelled neurons in the brainstem and hypothalamus. Retrogradely traced RFP-positive neurons (RbV, magenta) were characterized by

immunofluorescence staining or *in situ* hybridization. Arrows show co-labelled cells. **a, b)** In the ARC, RbV neurons expressed neither *Agrp* (**a**) nor *Pomc* (**b**). Scale bars a1, b1, 100 µm; magnified images, 25 µm. **c-g)** In the PVN, RbV-positive neurons expressed *Trh* (yellow, **c**), *Crh* (cyan, **d**), oxytocin (OXT, green, **e**), *somatostatin* (*Sst*, cyan, **f**), and arginine-vasopressin (AVP, yellow, **g**). Scale bars in c-g, 50 µm. **h-j)** While no RbV-positive neurons within the PVN were positive for TH (**h**), we found co-labelling of RbV-positive neurons with TH in the retrochiasmatic area (RCh) (**i**) and the LC (**j**). Scale bar h, 100 µm; i1 and j1, 50 µm, i2, i3, j2 and j3, 25µm. **k)** In the raphe nucleus, a TPH neuron was retrogradely traced by RbV. Scale bar k1, 50 µm; magnified images k2-3, 25 µm. **l)** In the POA, GnRH-expressing neurons were not retrogradely traced by RbV. Scale bar, 100 µm. **m)** *Chat in situ* hybridization showed that cholinergic neurons in the medial septal nucleus (MS) were retrogradely traced from tanycytes. Overview scale bar, 100 µm; magnified images, 25 µm. Detailed information on the sex of the animals is provided in Supplementary Data file 5.

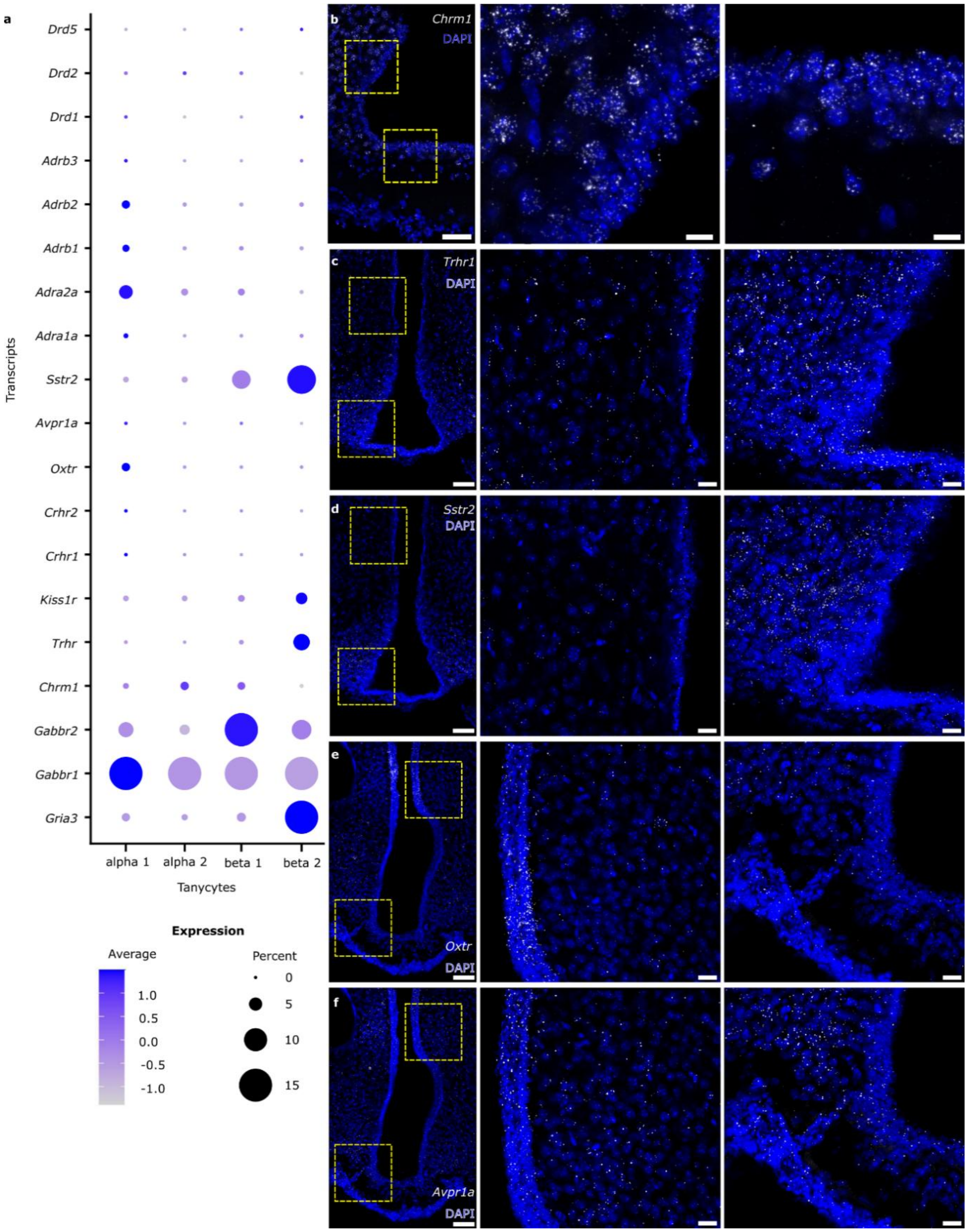

Supplementary Fig. 7. Tanycytes express RNA for neurotransmitter and neuropeptide receptors. a) ScRNAseq analysis of the expression of receptors whose neurons were identified by rabies tracing. The analysis indicates that expression levels differ between tanycyte subpopulations. **b-f)** *In situ* hybridization of *Chrm1* (**b**), *Trhr1* (**c**), *Sstr2* (**d**), *Oxtr* (**e**) and *Avpr1a* (**f**) showed that tanycytes expressed mRNA in accordance to the differential expression pattern shown by scRNAseq. Scale bar overviews 100 µm, magnified images 50 µm. Detailed information on the sex of the animals is provided in Supplementary Data file 5.

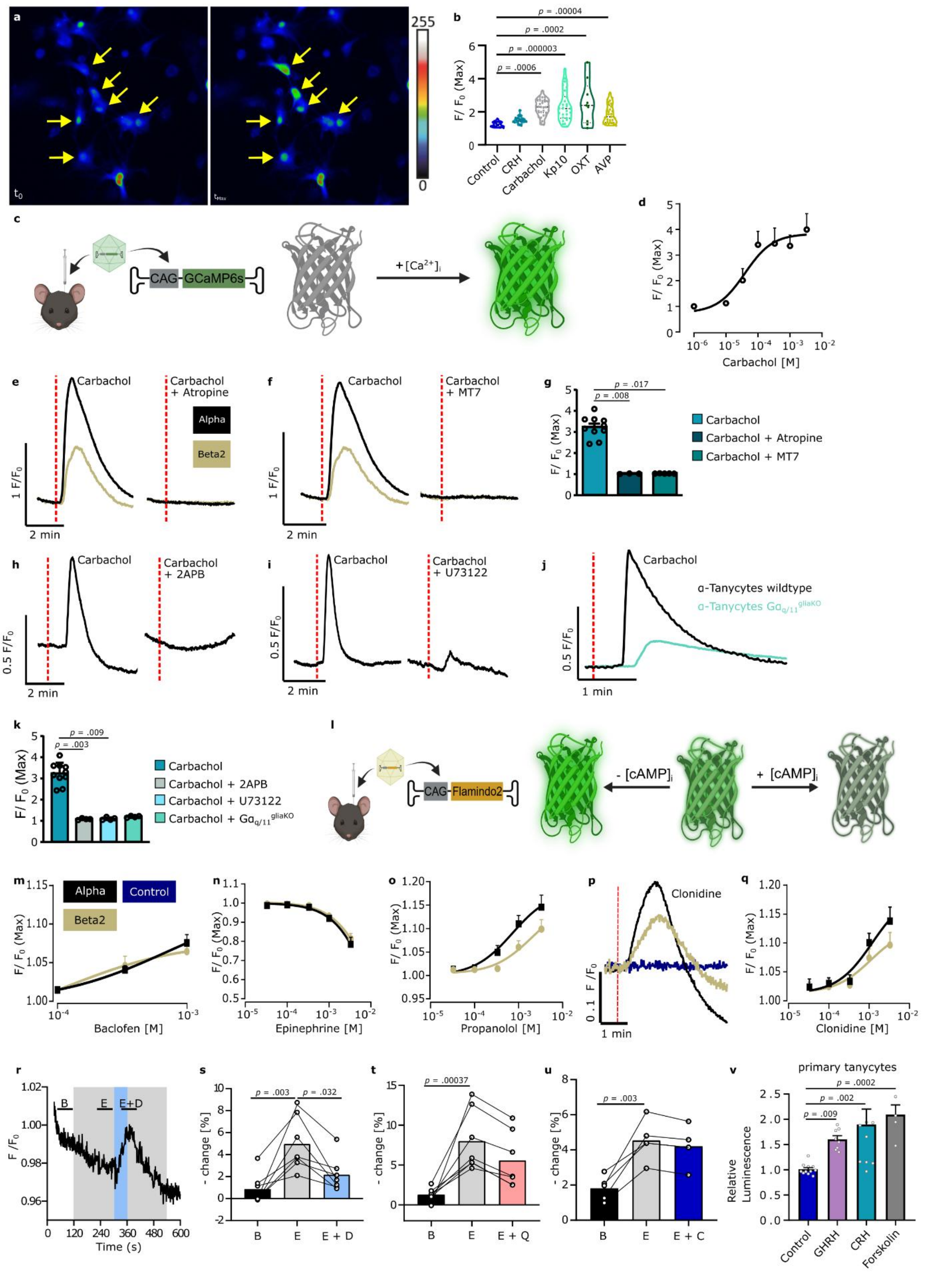

Supplementary Fig. 8. Imaging of intracellular calcium and cAMP concentrations after stimulating tanycytes with neurotransmitters. a) Fluorescence heatmaps of primary tanycytes stained with the calcium sensor Fluo4 before stimulation and at the maximum fluorescence after stimulation with 21 µM CRH. Arrows mark single tanycytes reacting to CRH with an increase $[Ca^{2+}]_i$. **b)** $[Ca^{2+}]_i$ in primary tanycytes was increased by CRH (21 µM), carbachol (1 mM), Kp10 (1 mM), and AVP (1 mM). Each dot represents a single tanycyte. Peak fluorescence intensities relative to baseline (F/F_0 max) are shown. For statistical analysis, two-tailed Kruskal-Wallis and targeted Dunn´s post hoc tests were performed. **c)** Injection of a tanycyte-specific AAV vector encoding the calcium sensor GCaMP6s

into the lateral ventricle of mice to express GCaMP6s in tanycytes. Fluorescence intensity of GCaMP6s is a measure of tanycytic $[Ca^{2+}]_i$. **d)** $[Ca^{2+}]_i$ in tanycytes of acute brain slices was increased by muscarinic receptor agonist carbachol to the perfusate in a concentration-dependent manner. Dots represent the mean of the F/F_0 max of 4 mice with 3 slices per animal. **e-k)** The stimulation of tanycytic $[Ca^{2+}]_i$ in acute slices by carbachol (333 µM) was inhibited by the muscarinic receptor blockers atropine (10 µM) and MT7 (0.5 µM), the IP3-receptor antagonist 2APB (100 µM), the phospholipase C inhibitor U73122 (100 µM) and the genetic deletion of $G\alpha_{q/11}$ proteins in $G\alpha_{q/11}^{gliaKO}$ mice. For statistical analysis, two-tailed Kruskal-Wallis and Dunn´s post hoc tests were performed. **l)** Injection of a tanycyte-specific AAV vector encoding the cAMP sensor Flamindo2 into the lateral ventricle of mice to express Flamindo2 in tanycytes. The fluorescence intensity of Flamindo2 decreases with increasing $[cAMP]_i$. **m-o)** Flamindo2 fluorescence in acute brain slices increased or decreased after treatment with baclofen (**m**, n = 8 mice), epinephrine (**n**, n = 6 mice), or propranolol (**o**, n = 6 mice) in a concentration-dependent manner. **p)** Clonidine (1 mM) lowered $[cAMP]_i$ in tanycytes of acute slices. Traces of the fluorescence relative to baseline (F/F_0) in the α- (black), β-tanycyte region (beige) are shown, representative of experiments in 11 mice. Red line, start of stimulation. **q)** Clonidine concentration-response relationship for $[cAMP]_i$ in tanycytes of acute slices. **r)** The increase in $[cAMP]_i$ stimulated by epinephrine (E, 500 µM, grey area) was reversed by dopamine (E+D, 500 µM, blue area). Representative traces are shown. B, baseline. **s-u**) Quantification of the maximal changes in tanycytic $[cAMP]_i$ induced by epinephrine (500 µM, E) with or without dopamine (500 µM, D), the selective D2R-agonist quinpirole (500 µM, Q) or vehicle as control (C) in acute brain slices. If cAMP production was stimulated, dopamine or the D2 agonist quinpirole were able to inhibit cAMP production. For statistical analysis, Friedman and targeted Dunn´s post hoc tests were performed. **v)** Stimulation of primary tanycytes with GHRH (1 µM), CRH (0.021 µM) or forskolin (0.25 µM) increased cAMP levels significantly compared to control. cAMP was measured by cAMP-Glo assay. For statistical analysis, two-tailed Kruskal-Wallis and targeted Dunn´s post hoc tests were performed. Representative traces of F/F_0 or the quantification of $F/F_{0\ Max}$ are shown. Each dot represents one mouse (**s-u**) or one well (**v**). **c** and **l** were created in BioRender. Schwaninger, M. (2026) https://BioRender.com/2o35jyv. Mean ± SEM. Detailed information on the test statistics, sex and number of the animals is provided in Supplementary Data file 5.

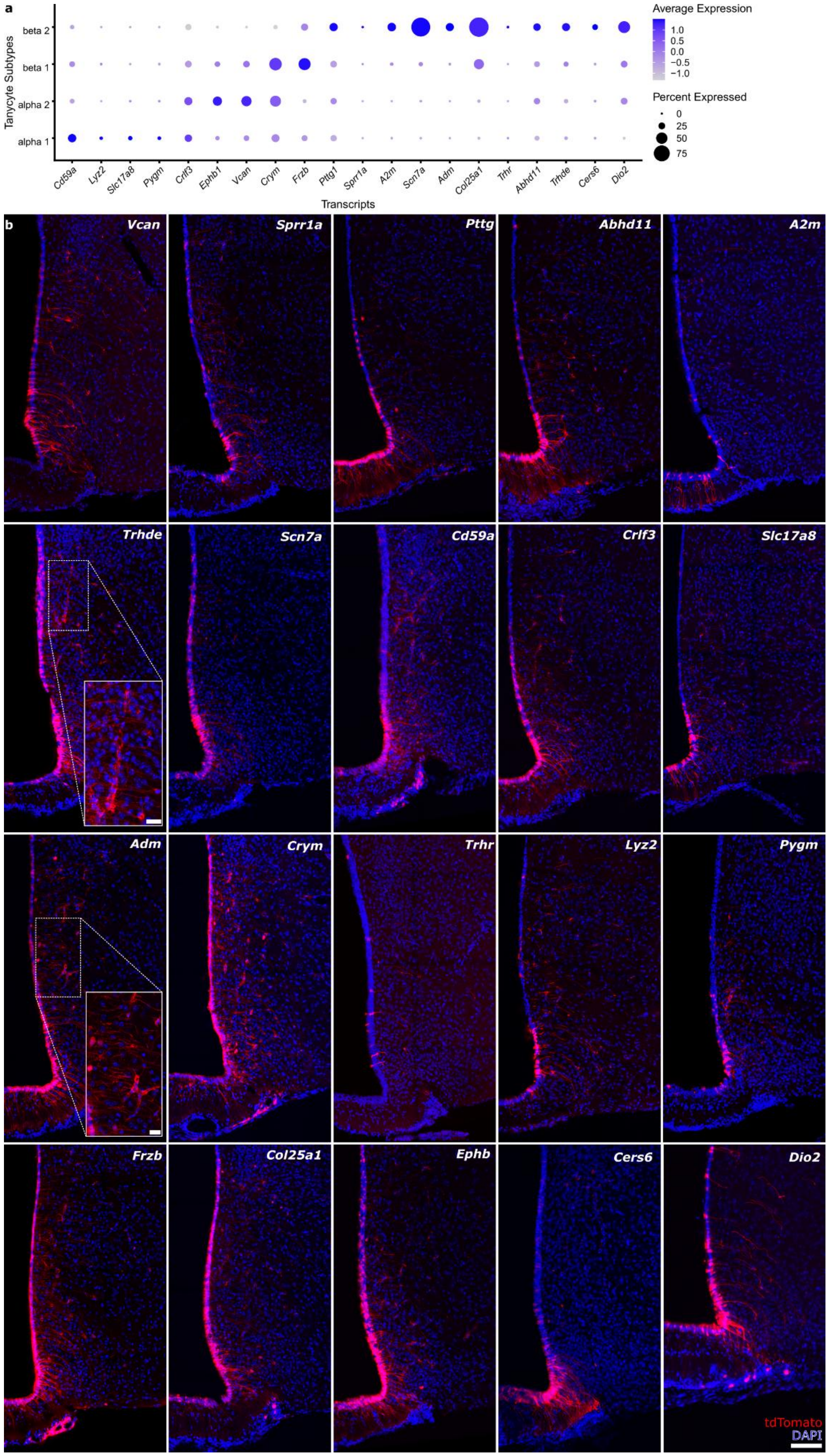

Supplementary Fig. 9. The promoter sequence of a panel of genes enables the targeting of

specific tanycyte subpopulations. a) ScRNAseq analysis demonstrates that a panel of 20 tanycyte genes exhibits a differential expression in subpopulations of tanycytes. The analysis is based on Tanybase scRNAseq data. **b)** AAV vectors encoding Cre under transcriptional control of the indicated gene promoters target different tanycyte subpopulations. In the top row the promoters with the highest specificity for subpopulations are shown from α-tanycytes (left) to β-tanycytes (right). Promoters in the following rows showed a broader specificity. Staining of the Cre reporter tdTomato on coronal sections is depicted after injecting the vectors into the lateral ventricle of *Ai14* mice. Scale bar, 100 µm, magnified images 20 µm. Detailed information about the reproducibility of the vectors is provided in Supplementary Data file 5.

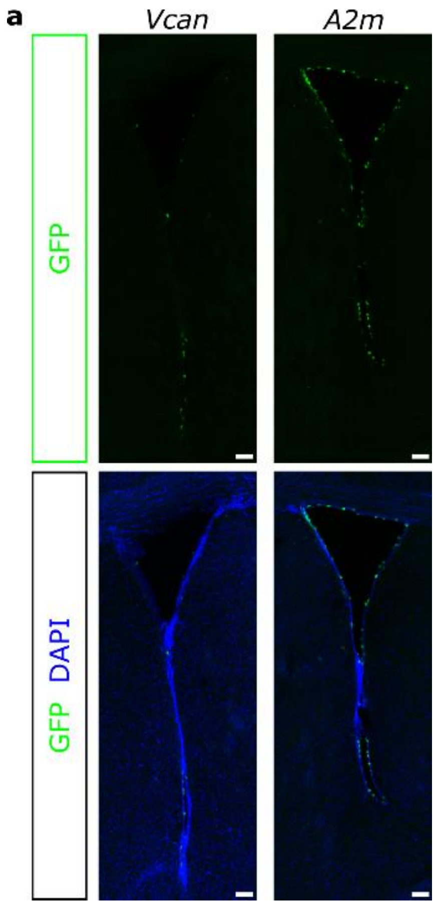

Supplementary Fig. 10. AAV-mediated transduction does not target the parenchyma. Mice that received *AAV-Vcan-Cre-2A-GFP* (*Vcan*) or *AAV-A2m-Cre-2A-GFP* (*A2m*) did not exhibit Gfp expression in the parenchyma, such as the subventricular zone. The images are representative of > 5 mice. Scale bars, 100 µm.

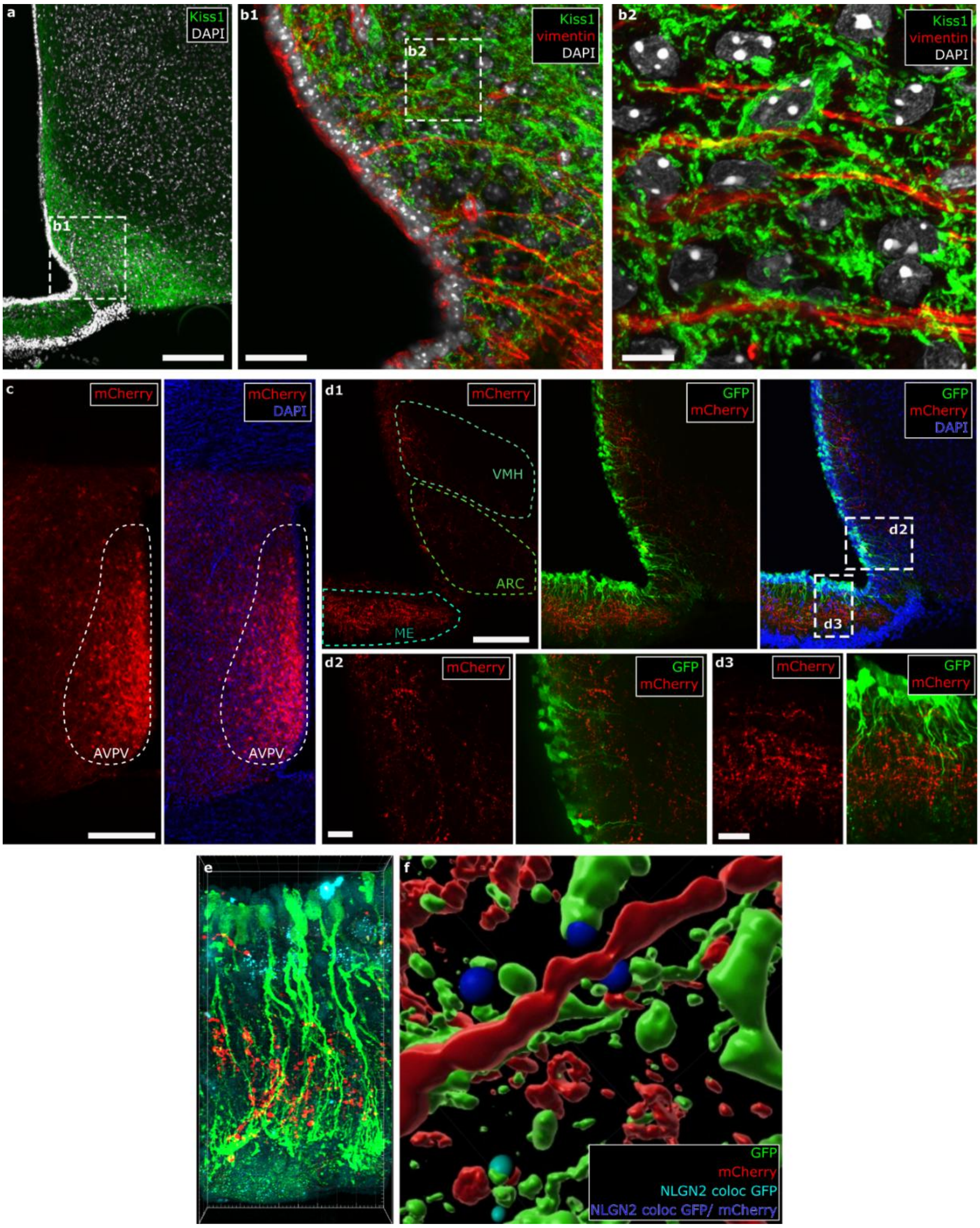

Supplementary Fig. 11. Kisspeptin (Kiss1) neurons project to tanycytes.
a, b) Labeling of Kiss1 neurons by the membrane-bound GFP (green) in *Kiss1-Cre*;*mT/mG* mice demonstrated that Kiss1 neurons send projections to vimentin-stained tanycytes (red) in arcuate nucleus and the median eminence. Images are representative for experiments in 5 mice. Scale bar **a**, 200 µm. Scale bar **b1**, 25 µm; magnified image **b2**, 10 µm. **c, d)** Labeling of Kiss1 neurons in the AVPV using *AAV-flex-mCherry* (red) in *Kiss1-Cre* mice. Kiss1 neurons and neurites in the site of stereotaxic injection (AVPV) (**c**) and the kisspeptin fiber projection into th ARC (**d**).Tanycytes labelled with GFP (green) by AAV-mediate transduction. The images are representative for experiments in 8 mice. Scale bar c, and d1 100 µm, magnified images d2 and d3, 25 µm. **e, f**) mCherry-labelled Kiss1 neurites in the median eminence (red) are closely apposed to NLGN2-positive post-synaptoid puncta (cyan) in GFP-labelled tanycytes (green) (**f**). 3D reconstruction shows NLGN2 puncta in less than 1 µm distance to mCherry in dark blue (**f**).

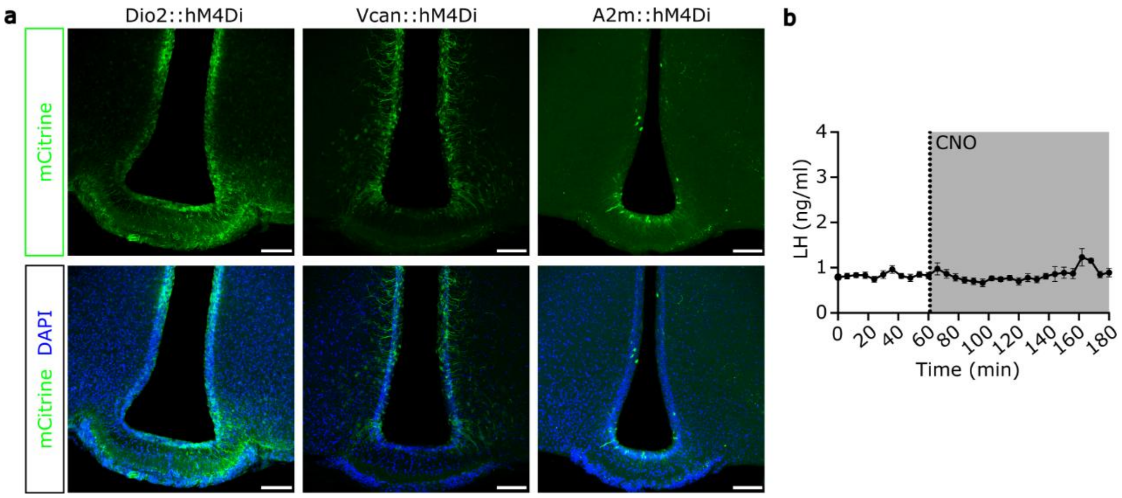

Supplementary Fig. 12. Specificity of the DREADD approach to inhibit tanycyte subpopulations. **a**) To target tanycyte subpopulations, *hM4Di* mice received either *AAV-Dio2-Cre* (α+β-tanycytes), *AAV-Vcan-Cre-2A-GFP* (α+β1-tanycytes), or *AAV-A2m-Cre-2A-GFP* (β-tanycytes). Expression of the mCitrine reporter in *hM4Di* mice demonstrated the specificity of the viral approach. Scale bars, 100 µm. **b**) When recombination was not induced in *hM4Di* mice, clozapine-N-oxide (CNO, 1 mg/kg body weight) had no significant effect on LH blood concentrations. Values are means ± SEM (*N* = 5 mice).

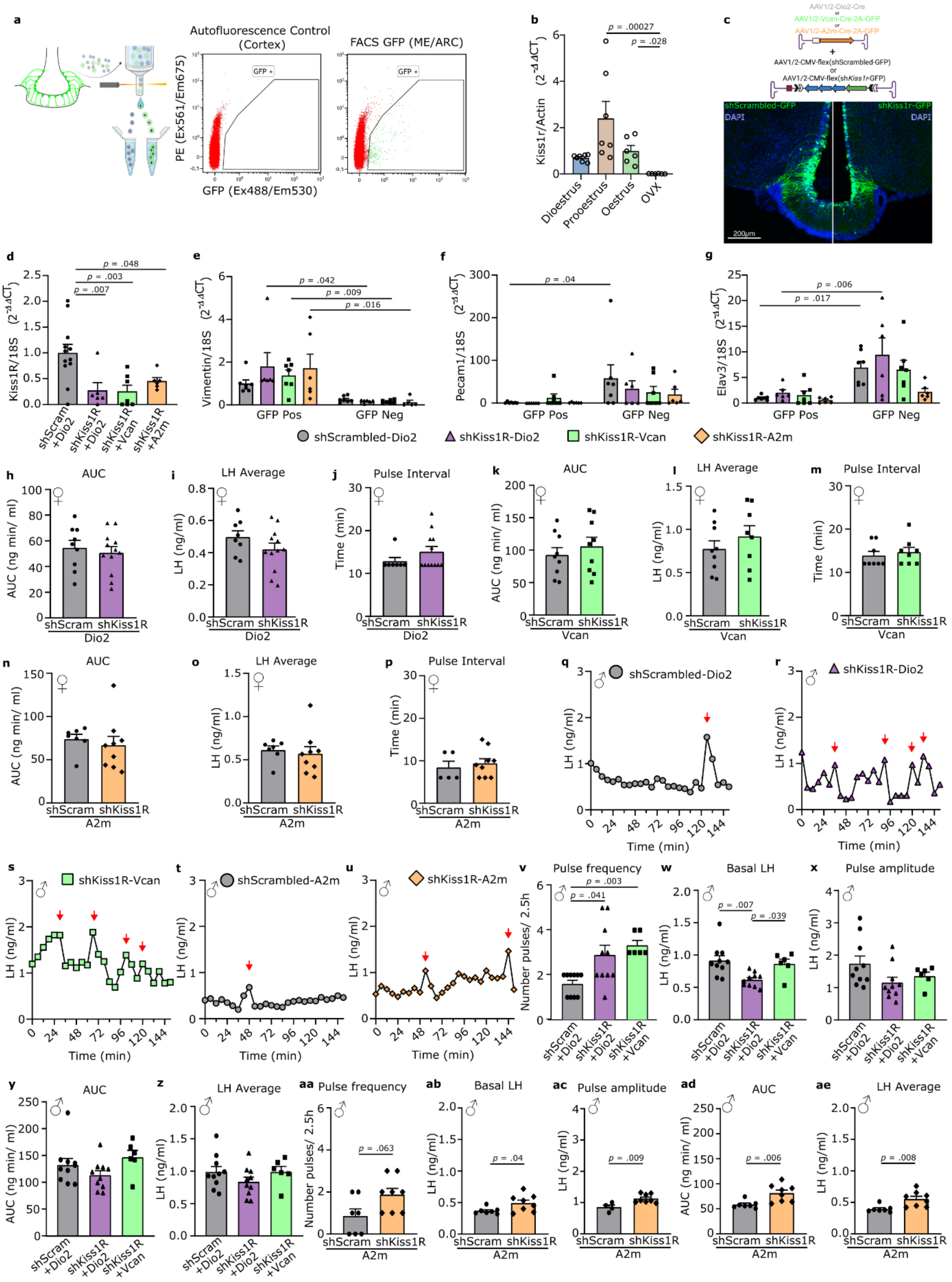

Supplementary Fig. 13. Tanycyte subpopulations mediate a differential effect of kisspeptin on LH secretion in both sexes. a) Scheme of FACS of fluorescently labelled tanycytes. Dot-plots showing sorted cells from the ME/ARC after dissection of AAV-injected animals and the gating strategy for GFP-positive cells. Parts created in BioRender. Schwaninger, M. (2026) https://BioRender.com/erufvsa. **b)** Real-time qPCR for *Kiss1r* in sorted tanycytes during different stages of the estrus cycle and in OVX mice. *AAV-Dio2-Cre* was injected in the lateral ventricle of *Ai14* mice to enable sorting of tanycytes. For

statistical analysis, two-tailed Kruskal-Wallis and Dunn´s post hoc tests were performed. **c)** Strategy of inducing *Kiss1r* knockdown in tanycytes by co-injection of tanycyte specific AAVs encoding Cre-recombinase together with either *AAV-flex-shKiss1r-GFP* or *AAV-flex-shScramble-GFP*, respectively. Confocal images confirming the transduction of tanycytes with *AAV-flex-shScramble-GFP* (left) and *AAV-flex-shKiss1r-GFP* (right) together with *AAV-Dio2-Cre*. **d-g)** mRNA of *Kiss1r* (**d**), the tanycytic marker gene *Vimentin* (**e**), and the non-tanycytic genes *Pecam1* (**f**), and *Elav3* (**g**) in GFP-positive tanycytes and GFP-negative cells isolated by FACS from mice treated either by *AAV-flex-shScramble-GFP* or *AAV-flex-shKiss1r-GFP* together with either *AAV-Dio2-Cre, AAV-Vcan-Cre-2A-GFP, or AAV-A2m-Cre-2A-GFP*. mRNAs were determined by RT-qPCR. For statistical analysis, one-way ANOVA followed by targeted Dunnett's post hoc tests (**d**) or RM-two-way ANOVA followed by targeted Sidak´s post-hoc tests (**e-g**) were performed. **h-p)** Area under the curve (AUC), LH average concentrations, and pulse intervals in female mice in which *Kiss1r* was knocked down in *Dio2*-targeted α+β-tanycytes (**h-j**), in *Vcan*-targeted α+β1-tanycytes (**k-m**), or in *A2m*-targeted β-tanycytes (**n-p**). **q-u)** Representative LH profiles in male mice treated with *AAV-flex-shScramble-GFP* plus *AAV-Dio2-Cre* (**q**) or *AAV-A2m-Cre-2A-GFP* (**t**); with *AAV-flex-shKiss1R-GFP* plus *AAV-Dio2-Cre* (**r**), *AAV-Vcan-Cre-2A-GFP* (**s**), or *AAV-A2m-Cre-2A-GFP* (**u**). Time points considered as a LH pulse were labelled with red arrows. **v-z)** LH pulse frequency, basal LH blood concentrations, pulse amplitude, AUC, and average LH concentrations in male mice in which *Kiss1r* was knocked down in *Dio2*-targeted α+β-tanycytes or in *Vcan*-targeted α+β1-tanycytes. **aa-ae**) LH pulse frequency, basal LH concentrations, pulse amplitude, AUC, and average LH concentrations in male mice in which *Kiss1r* was knocked down in *A2m*-targeted β-tanycytes. Values are means ± SEM, *N* denotes the number of mice. Detailed information on the test statistics, sex and number of the animals is provided in Supplementary Data file 5.

References

1. Mori, T.*, et al.* Inducible gene deletion in astroglia and radial glia--a valuable tool for functional and lineage analysis. *Glia* **54**, 21-34 (2006).
2. Muzumdar, M.D., Tasic, B., Miyamichi, K., Li, L. & Luo, L. A global double-fluorescent Cre reporter mouse. *Genesis* **45**, 593-605 (2007).
3. Madisen, L.*, et al.* A robust and high-throughput Cre reporting and characterization system for the whole mouse brain. *Nat Neurosci* **13**, 133-140 (2010).
4. Wettschureck, N.*, et al.* Absence of pressure overload induced myocardial hypertrophy after conditional inactivation of Galphaq/Galpha11 in cardiomyocytes. *Nat Med* **7**, 1236-1240 (2001).
5. Navarro, V.M.*, et al.* Regulation of NKB Pathways and Their Roles in the Control of Kiss1 Neurons in the Arcuate Nucleus of the Male Mouse. *Endocrinology* **152**, 4265-4275 (2011).
6. Zhu, H.*, et al.* Cre-dependent DREADD (Designer Receptors Exclusively Activated by Designer Drugs) mice. *Genesis* **54**, 439-446 (2016).
7. Müller-Fielitz, H.*, et al.* Tanycytes control the hormonal output of the hypothalamic-pituitary-thyroid axis. *Nat Commun* **8**, 484 (2017).
8. Schmid, L.C.*, et al.* Dysfunction of Somatostatin-Positive Interneurons Associated with Memory Deficits in an Alzheimer's Disease Model. *Neuron* **92**, 114-125 (2016).
9. Böttcher, M.*, et al.* NF-kappaB signaling in tanycytes mediates inflammation-induced anorexia. *Molecular metabolism* **39**, 101022 (2020).
10. Prevot, V., Cornea, A., Mungenast, A., Smiley, G. & Ojeda, S.R. Activation of erbB-1 signaling in tanycytes of the median eminence stimulates transforming growth factor beta1 release via prostaglandin E2 production and induces cell plasticity. *J Neurosci* **23**, 10622-10632 (2003).
11. Chandrasekar, A.*, et al.* Regulation of Thyroid Hormone Gatekeepers by Thyrotropin in Tanycytes. *Thyroid* **34**, 261-273 (2024).
12. Aggarwal, A.*, et al.* Glutamate indicators with improved activation kinetics and localization for imaging synaptic transmission. *Nature Methods* **20**, 925-934 (2023).
13. Ciofi, P., Leroy, D. & Tramu, G. Sexual dimorphism in the organization of the rat hypothalamic infundibular area. *Neuroscience* **141**, 1731-1745 (2006).
14. Kruzich, E.*, et al.* A pipeline for STED super-resolution imaging and Imaris analysis of nanoscale synapse organization in mouse cortical brain slices. *STAR Protoc* **4**, 102707 (2023).
15. Tameling, C.*, et al.* Colocalization for super-resolution microscopy via optimal transport. *Nat Comput Sci* **1**, 199-211 (2021).
16. Cora, M.C., Kooistra, L. & Travlos, G. Vaginal Cytology of the Laboratory Rat and Mouse: Review and Criteria for the Staging of the Estrous Cycle Using Stained Vaginal Smears. *Toxicologic pathology* **43**, 776-793 (2015).
17. Czieselsky, K.*, et al.* Pulse and Surge Profiles of Luteinizing Hormone Secretion in the Mouse. *Endocrinology* **157**, 4794-4802 (2016).
18. Steyn, F.J.*, et al.* Development of a methodology for and assessment of pulsatile luteinizing hormone secretion in juvenile and adult male mice. *Endocrinology* **154**, 4939-4945 (2013).
19. Kreisman, M.J., McCosh, R.B. & Breen, K.M. A Modified Ultra-Sensitive ELISA for Measurement of LH in Mice. *Endocrinology* **163**(2022).
20. Duquenne, M.*, et al.* Leptin brain entry via a tanycytic LepR-EGFR shuttle controls lipid metabolism and pancreas function. *Nat Metab* **3**, 1071-1090 (2021).
21. Langlet, F.*, et al.* Tanycytic VEGF-A boosts blood-hypothalamus barrier plasticity and access of metabolic signals to the arcuate nucleus in response to fasting. *Cell Metab* **17**, 607-617 (2013).

22. Parkash, J., *et al.* Semaphorin7A regulates neuroglial plasticity in the adult hypothalamic median eminence. *Nat Commun* **6**, 6385 (2015).
23. Renier, N., *et al.* Mapping of Brain Activity by Automated Volume Analysis of Immediate Early Genes. *Cell* **165**, 1789-1802 (2016).
24. Belle, M., *et al.* Tridimensional Visualization and Analysis of Early Human Development. *Cell* **169**, 161-173 e112 (2017).
25. Steuernagel, L., *et al.* HypoMap-a unified single-cell gene expression atlas of the murine hypothalamus. *Nat Metab* **4**, 1402-1419 (2022).
26. Sullivan, A.I., Potthoff, M.J. & Flippo, K.H. Tany-Seq: Integrated Analysis of the Mouse Tanycyte Transcriptome. *Cells* **11**(2022).
27. Campbell, J.N., *et al.* A molecular census of arcuate hypothalamus and median eminence cell types. *Nat Neurosci* **20**, 484-496 (2017).
28. Chen, R., Wu, X., Jiang, L. & Zhang, Y. Single-Cell RNA-Seq Reveals Hypothalamic Cell Diversity. *Cell reports* **18**, 3227-3241 (2017).
29. Prevot, V., Dutoit, S., Croix, D., Tramu, G. & Beauvillain, J.C. Semi-quantitative ultrastructural analysis of the localization and neuropeptide content of gonadotropin releasing hormone nerve terminals in the median eminence throughout the estrous cycle of the rat. *Neuroscience* **84**, 177-191 (1998).
