# Supplementary material for: A synaptoid connectome differentiates tanycytic subpopulations and underlies neuroglial communication and neuroendocrine regulation
Source: Nat Commun. 2026 Jun 20;17:7797. doi: 10.1038/s41467-026-74598-5 (PMC13439102; doi:10.1038/s41467-026-74598-5)
Supplement: Supplementary file 4 [file 41467_2026_74598_MOESM2_ESM.pdf]

Title: Supplementary Video 1

Description: Glutamate imaging under baseline conditions. Recording of an acute brain slice in the median eminence of AAV-CAG-iGluSnFR3 transduced mice, performed two weeks after AAV injection. The original recording lasted 5 min (300 images at 1 frame per second), compressed to a playback speed of 20 frames per second. Fluorescence intensity is visualized using a false color scale (Rainbow RGB) in FIJI.

Title: Supplementary Video 2

Description: Glutamate imaging after glutamate application. Fluorescence recording of the median eminence of an AAV-CAG-iGluSnFR3-transduced mouse, two weeks after AAV injection. Glutamate (1 mM) was applied via perfusion for 30 seconds, starting 30 seconds after the beginning of the recording. The original recording lasted 5 min (300 images at 1 frame per second), compressed to a playback speed of 20 frames per second. Fluorescence intensity is visualized using a false color scale (Rainbow RGB) in FIJI.

Title: Supplementary Video 3

Description: Glutamate imaging after KCl application – 1. Fluorescence recording of the median eminence of an AAV-CAG-iGluSnFR3-transduced mouse, two weeks after AAV injection. KCl (30 mM) was applied via perfusion for 30 seconds, starting 30 seconds after the beginning of the recording. The original recording lasted 5 min (300 images at 1 frame per second), compressed to a playback speed of 20 frames per second. Fluorescence intensity is visualized using a false color scale (Rainbow RGB) in FIJI.

Title: Supplementary Video 4

Description: Glutamate imaging after KCl application – 2. Fluorescence recording of a single tanycyte (shown in Fig 1j) in an AAV-CAG-iGluSnFR3 transduced mouse, two weeks after AAV injection. KCl (30 mM) was applied via perfusion for 30 seconds, starting 30 seconds prior to the beginning of the recording. The original recording consisted of 700 images acquired at 1 frame every 0.5 seconds, compressed to a playback speed of 7 frames per second. Fluorescence intensity is visualized using a false color scale (Rainbow RGB) in FIJI.

Title: Supplementary Video 5

Description: Glutamate imaging after KCl application shown as surface plot. Surface plot of the fluorescence recording in the median eminence from an AAV-CAG iGluSnFR3-transduced mouse, two weeks after AAV injection. The corresponding original image is shown in Supplementary Fig. 3c. KCl (30 mM) was applied via perfusion for 30 seconds, beginning 30 seconds after the start of the recording. The original recording lasted 5 min (300 images at 1 frame per second), compressed to a playback

speed of 20 frames per second. Fluorescence intensity is visualized using a false color scale (Rainbow RGB) in FIJI. The summed time stack is shown in Fig. 1l.

Title: Supplementary Video 6

Description: Glutamate imaging after KCl application in the presence of TTX. Surface plot of the fluorescence recording in the median eminence from an AAV-CAG iGluSnFR3-transduced mouse, two weeks after AAV injection. The corresponding original image is shown in Supplementary Fig. 3c. KCl (30 mM) was applied via perfusion for 30 seconds, beginning 30 seconds after the start of the recording. TTX (1 µM) was included in the perfusate 15 min prior to KCl stimulation. The original recording lasted 5 min (300 images at 1 frame per second), compressed to a playback speed of 20 frames per second. Fluorescence intensity is visualized using a false color scale (Rainbow RGB) in FIJI. The summed time stack is shown in Fig. 1l.

Title: Supplementary Video 7

Description: Glutamate imaging after KCl application and washout of TTX. Surface plot of the fluorescence recording in the median eminence from an AAV-CAG iGluSnFR3-transduced mouse, two weeks after AAV injection. The corresponding original image is shown in Supplementary Fig. 3c. KCl (30 mM) was applied via perfusion for 30 seconds, beginning 30 seconds after the start of the recording. TTX was washed out for 15 min prior to KCl stimulation. The original recording lasted 5 min (300 images at 1 frame per second), compressed to a playback speed of 20 frames per second. Fluorescence intensity is visualized using a false color scale (Rainbow RGB) in FIJI. The summed time stack is shown in Fig. 1l.

Title: Supplementary Video 8

Description: Selective tracing of Kiss1 neurons from the AVPV. AAV-flex-mGFP was injected into the AVPV of a Kiss1-Cre mouse, resulting in the labelling of the membrane with GFP. Following tissue clearing and light sheet microscopy, the projection of AVPV Kiss1 neurons was observed to contain axons extending into various brain regions, including the ME.

Title: Supplementary Data file 1

Description: Primers used to amplify promoter segments of tanycyte genes.

Title: Supplementary Data file 2

Description: Antibodies used for immunofluorescence, Western blot and ELISA.

Title: Supplementary Data file 3

Description: Image acquisition settings for STED.

Title: Supplementary Data file 4

Description: Viral vectors and doses.

Title: Supplementary Data file 5

Description: Sample size, sex of the animals and results of statistical tests.
