# Supplementary material for: A synaptoid connectome differentiates tanycytic subpopulations and underlies neuroglial communication and neuroendocrine regulation
Source: Nat Commun. 2026 Jun 20;17:7797. doi: 10.1038/s41467-026-74598-5 (PMC13439102; doi:10.1038/s41467-026-74598-5)
Supplement: Supplementary file 5 [file 41467_2026_74598_MOESM3_ESM.pdf]

Supplementary Data file 1. Primers used to amplify promoter segments of tanycyte genes.

Gene	Direction	Sequence	Gene Locus	Amplificate length [bp]
A2m	Forward<br>Reverse	5‘-GTC AGA ACG CGT AAA GCC AAC TCC CTT GAC CAC-3‘<br>5‘-CAG TAA ACC GGT CGG TGG ACT CAC GGT TTC TG-3‘	NM 175628.3 [-1322/122]	1,444
Abhd11	Forward<br>Reverse	5‘-ACG TCA ACG CGT CCT GTG AGG AGA CAG AGA GA-3‘<br>5‘-ACG TCA TCT AGA AGT AAG GGG GAG GAG AGA GT-3‘	NM 145215 [-1173/248]	1,421
Adm	Forward<br>Reverse	5‘-ACG TCA ACG CGT GGG GTC GCC AGG AAG AGG AC-3‘<br>5‘-ACG TCA GCT AGC CCC AAA GTC CAA GAT TGA CT-3‘	NM 009627 [-1301/101]	1,402
Cd59a1	Forward<br>Reverse	5‘-AGT CGA ACG CGT TGT CAT TTG GGA CAC AGC AG-3‘<br>5‘-AGT CGA ACC GGT AGA CAC TTT TCA GAT CGC CAG T-3‘	NM 007652.6 [-990/+32]	1,022
Cers6	Ordered via vectorbuilder		NC 000068 [-812/+695]	1507
Col25a1	Forward<br>Reverse	5‘-AGT GCA ACG CGT AGA GAT GAC AGT CTG GAG TTG GTT C-3‘<br>5‘-TGA GAC ACC GGT ACC CAA GCA GCG ATC GTA AGC TG-3‘	NM 198711 [-1325/43]	1,368
Crlf3	Forward<br>Reverse	5‘-AGT CGA ACG CGT CAC TGG CTG GGA CTA AAG GGG TT-3‘<br>5‘-ACT GAG ACC GGT GAA GCA GCA CCT CGG GCT CC-3‘	NM 018776 [-1399/71]	1,470
Crym	Forward<br>Reverse	5‘-ACG TCA ACG CGT CAC TCT CAC CTT TAG TGT GT-3‘<br>5‘-ACG TCA ACC GGT GCT GAA ACA CAC TCC ACG TT-3‘	NM 016669 [-1500/37]	1,537
Ephb1	Forward<br>Reverse	5‘-ACG TAA CGC GTA GGT AAG AGC TTT GGG GAT TC-3‘<br>5‘-ACT GAT CTA GAT GTG CGT CCT GGT GTC A-3‘	NM 173447 [-1439/66]	1,505
Frzb	Forward<br>Reverse	5‘-ACG TCA ACG CGT CCC TAG CCA CCA TCT GTT GT-3‘<br>5‘-ACG TCA TCT AGA TGC AGG GTC GGG AAG AT-3‘	NM 011356 [-1371/51]	1,422
Lyz2	Forward<br>Reverse	5‘-ACG TCA ACG CGT AAG ATT AAA TCT TGT CAC TG-3‘<br>5‘-ACG TCA TCT AGA CAA CGT TCA TAG ACC TTG GC-3‘	NM 017372 [-1501/102]	1,603
Pttg1	Forward<br>Reverse	5‘-ACT GCA ACG CGT GCT TTC TTG ATC TAC CAC TCA CC-3‘<br>5‘-ACG TCA TCT AGA GCT AAA CTC ACC GAA GAC TGC-3‘	NM 013917 [-1327/41]	1,368
Pygm1	Forward<br>Reverse	5‘-ATG CTA ACG CGT TGA CAG ACA AGG CTC CTT GA-3‘<br>5‘-ACG TAC TCT AGA AGA TGA TGG CCT CCT AGC TG-3‘	NM 011224 [-1401/102]	1,503
Scn7a	Forward<br>Reverse	5‘-AGT CGA ACG CGT AAA TGT GGG CAA GAA AAA CTG GAA-3‘<br>5‘-AGT CGA ACC GGT AAA ACT CCA GCA AGA ACC CCA-3‘	NM 009135.2 [-961/181]	1,142

Slc17a8	Forward<br>Reverse	5‘-ACG TCA ACG CGT GAA AGA AGC CAG CCA CAA GA-3‘<br>5‘-ACG TCA ACC GGT CAG CCC CTT GCA AAA AGC TA-3‘	NM 182959 [-1501/72]	1,573
Sprr1a	Forward<br>Reverse	5‘-ACG TCA ACG CGT TCT TTG AAT CAG GGT TAC CC-3‘<br>5‘-ACG TCA ACC GGT CAA ATC AGT TGC AAG CAG AGA C-3‘	NM 009264 [-1476/81]	1,557
Thrde	Forward<br>Reverse	5‘-AGT CGA ACG CGT TCT GGC GAG CTT GAT GTG TG-3‘<br>5‘-AGT CGA ACC GGT TCA CTC CCA CCG AGT CAG C-3‘	NM 146241 [-900/203]	1,103
Trhr1	Forward<br>Reverse	5‘-ACG TCA ACG CGT CAC CTG AAG GCT GTG TGT GG-3‘<br>5‘-ACG TCA TCT AGA CAG GAA GAT CAC TTA GCA GA-3‘	NM 013696 [-1301/102]	1,403
Vcan	Forward<br>Reverse	5‘-ACT GCA ACG CGT GAA GCC CTG TGT TTG ATG TC-3‘<br>5‘-ACG TCA ACC GGT TGA GTT CTT CGC CCA GA-3‘	NM 172955 [-1325/21]	1,346
