# Supplementary material for: A synaptoid connectome differentiates tanycytic subpopulations and underlies neuroglial communication and neuroendocrine regulation
Source: Nat Commun. 2026 Jun 20;17:7797. doi: 10.1038/s41467-026-74598-5 (PMC13439102; doi:10.1038/s41467-026-74598-5)
Supplement: Supplementary file 6 [file 41467_2026_74598_MOESM4_ESM.pdf]

Supplementary Data file 2. Antibodies used for immunofluorescence (IF), Western blot (WB) and ELISA.

Antigen	Host species	Source, Catalogue number	Dilution or final concentration	Method	Imaging
GFP	Chicken	Abcam, #AB13970	1:1,000	IF	Confocal
	Rabbit	Abcam #AB290	1:400		STED
	Chicken	Aves-Lab, #GFP-1020	1:1,000	IF	Confocal
	Rabbit	Invitrogen #A6455	1:5,000	IF	Lightsheet
Bassoon	Chicken	Synaptic System, #141016	1:250	IF	Confocal
			1:250		STED
Gephyrin	Guinea pig	Synaptic System, #147318	1:500	IF	Confocal
			1:250		STED
NLGN2	Rabbit	Synaptic System #129203	1:500	IF, WB	Confocal
			1:500		STED
			1:1,000		Fusion Solo S
NLGN2	Guinea pig	Synaptic System #129518	1:800	IF	STED
PSD95	Rabbit	Cell Signalling Technology #3450	1:500	IF, WB	Confocal
			1:600		STED
			1:1,000		Fusion Solo S
Shank2	Guinea pig	Synaptic System #162204	1:500	IF	Confocal
			1:250		STED
Homer1	Chicken	Synaptic System #160026	1:500	IF	Confocal
			1:600		STED
VGLUT2	Chicken	Synaptic System #135409	1:400	IF	STED
VGAT	Chicken	Synaptic System #131009	1:400	IF	STED
Synaptophysin1	Chicken	Synaptic System #101009	1:400	IF	STED
MCT8	Rabbit	Atlas antibodies #HPA072719	1:1,000	IF	Confocal
			1:600		STED
GnRH	Rabbit	Homemade[29]	1:500	IF	Confocal
DsRed	Rabbit	Takara Bio # 632496	1:500	IF	Confocal
mCherry	Rat	Thermo Scientific #M11217	1:500	IF	Confocal
Oxytocin	Rabbit	Immunostar #20068	1:500	IF	Confocal
Tryptophan-hydroxylase 2	Rabbit	Novus Biologicals #NB-100-74555	1:500	IF	Fluorescence

Arginine-Vasopressin	Rabbit	Immunostar #20069	1:500	IF	Confocal
Vimentin	Chicken	Thermo Fisher Scientific #PA1-16759	1:500	IF	Confocal
	Chicken	Sigma Aldrich #AB5733	1:1,000	IF	Confocal
LHβ518B7	Mouse	UC Davis #518B7	1 µg/ml	Elisa	Varioskan
AAV VP1/ VP2/ VP3	Mouse	Progen #61058S	1:250	WB	Fusion Solo S
Mouse Fab	Goat	Jackson ImmunoResearch #115-007-003		IF	
			1:100		STED
Rabbit IgG	Goat	Abberior StarGreen #STGREEN-1002-500UG	1:1,000	IF	Confocal
			1:400		STED
Rabbit IgG	Goat	Abberior StarRed #STRED-1002-500UG	1:1,000	IF	Confocal
			1:600		STED
Rabbit Alexa Fluor 647	Goat	Invitrogen #A21244	1:1,000	IF	Lightsheet
5303 SPRN-5	Mouse	Medix Biochemica #100588	4 µg/ml	Elisa	Varioskan
Guinea pig IgG	Goat	Abberior #STORANGE-1006-500UG	1:1,000	IF	Confocal
			1:400		STED
Chicken IgY Alexa Fluor 488	Donkey	Jackson ImmunoResearch Labs #703-545-155	1:1,000	IF	Confocal
Chicken IgY Alexa Fluor 633	Goat	Thermo Fisher (#A21103)	1:1,000	IF	Confocal
Rabbit IgG Alexa Fluor 488	Donkey	Thermo Fisher Scientific (#A-21206)	1:1,000	IF	Confocal
Rabbit IgG Alexa Fluor 555	Donkey	Molecular Probes (#A-31572)	1:1,000	IF	Confocal
Rabbit IgG Alexa Fluor 647	Donkey	Thermo Fisher Scientific (#A-31573)	1:1,000	IF	Confocal
Rabbit IgG HRP	Goat	DakoCytomation #P0448	1:5,000	WB	Fusion Solo S
Guinea pig IgG HRP	Rabbit	Invitrogen #61-4620	1:5,000	WB	Fusion Solo S
Mouse IgG HRP	Goat	Jackson #115-035-003	1:2,500	WB	Fusion Solo S
