# Supplementary material for: A synaptoid connectome differentiates tanycytic subpopulations and underlies neuroglial communication and neuroendocrine regulation
Source: Nat Commun. 2026 Jun 20;17:7797. doi: 10.1038/s41467-026-74598-5 (PMC13439102; doi:10.1038/s41467-026-74598-5)
Supplement: Supplementary file 7 [file 41467_2026_74598_MOESM5_ESM.pdf]

Supplementary Data file 3. Image acquisition settings for STED.

Marker	2nd anti-body	xyz [nm]	Dwell time [µs]	Line Re-peat	Conf [%]	STED [%]	3D-STED /CONF	Ratio [2D/3D]	STED repeat	Emis-sion Range [nm]
**3D STED imaging.** The image size was always 20 x 20 x 0.6 µm^3.										
SHANK2	StarRed	60 x 60 x 60	30	2	10	12	3D-STED	20/80	5	660-763
GEPHYRIN	StarRed	60 x 60 x 60	30	2	11	12	3D-STED	20/80	5	660-763
HOMER1	StarRed	60 x 60 x 60	30	2	10	11	3D-STED	20/80	5	660-763
PSD95	StarRed	60 x 60 x 60	30	2	10	11	3D-STED	20/80	4	660-763
NLGN2	StarRed	60 x 60 x 60	30	2	11	12	3D-STED	20/80	5	660-763
BASSOON	StarOrange	60 x 60 x 60	30	3	10	11	3D-STED	20/80	5	580-660
GFP	StarGreen	60 x 60 x 60	50	2	8		CONF			500-540
MCT8	StarGreen	60 x 60 x 60	50	2	8		CONF			500-540
**2D STED for OTC**. The image size was 6 x 6 µm^2										
SHANK2	StarRed	40 x 40	20	2	10	17	2D-STED	80/20	4	660-763
HOMER1	StarOrange	40 x 40	20	2	10	17	2D-STED	80/20	4	580-640
NLGN2	StarOrange	40 x 40	30	2	10	17	2D-STED	80/20	4	580-640
MCT8	StarGreen	40 x 40	50	2	24	7	2D-STED	80/20	4	500-540
