# Supplementary material for: A synaptoid connectome differentiates tanycytic subpopulations and underlies neuroglial communication and neuroendocrine regulation
Source: Nat Commun. 2026 Jun 20;17:7797. doi: 10.1038/s41467-026-74598-5 (PMC13439102; doi:10.1038/s41467-026-74598-5)
Supplement: Supplementary file 8 [file 41467_2026_74598_MOESM6_ESM.pdf]

Supplementary Data file 4. Viral vectors and doses.

If not specified otherwise, mice and AAV1/2 vectors were used.

Figure	Viral Vectors	Vector dose
1d	*Dio2-Cre-2A-GFP*	5×10^8
1i	*EF1a-GPHN-FingR-eGFP*	2×10^9
1 j-l<br>Supplementary 3<br>Supplementary Video 1-7	*CAG-iGluSnFR3*	2.6×10^9
2 b-h<br>3a<br>3b<br><br>Supplementary 4 a-d<br>Supplementary 5<br>Supplementary 6	*EF1a-TVA-G*<br>*RbV-RFP*	7.4×10^8<br>1.25×10^7
3d-m	*CAG-GCaMP6s*	2.6×10^9
3n-p<br>Supplementary 8m-u	*CAG-Flamindo2*	$2.4\text{x}10^9$
4a	*Dio2-Cre-2A-GFP*	5×10^8
4b	*Vcan-Cre-2A-GFP*	1×10^9
4c	*A2m-Cre-2A-GFP*	5×10^8
4 e-g<br>Supplementary 4 e-h	*EF1a-TVA-G*<br>*Vcan-Cre-2a-GFP*<br>*RbV-RFP*	5.4×10^8<br>1.95×10^9<br>1.25×10^7
4 h1	*EF1a-TVA-G*<br>*RbV-RFP*	7.4×10^8<br>1.25×10^7
4 h2	*EF1a-TVA-G*<br>*Vcan-Cre-2a-GFP*<br>*RbV-RFP*	5.4×10^8<br>1.95×10^9<br>1.25×10^7
4 h3	*RbV-RFP*	1.25×10^7
4 i1	*EF1a-TVA-G*<br>*RbV-GFP*	7.4×10^8<br>1.25×10^7
4 i2	*EF1a-TVA-G*<br>*Vcan-Cre-2a-GFP*<br>*RbV-RFP*	5.4×10^8<br>1.95×10^9<br>1.25×10^7
5a<br>Supplementary 12 a	*Dio2-Cre*	1.25×10^9
5b<br>Supplementary 12 b	*Vcan-Cre-2A-GFP*	1.2×10^9
5c<br>Supplementary 12 c	*A2m-Cre-2A-GFP*	1.1×10^9
5d, f-h	*Dio2-Cre*<br>*flex-shScramble-GFP*	1.25×10^9<br>2.5×10^9
5e, f-h	*Dio2-Cre*<br>*flex-shKiss1r-GFP*	1.25×10^9<br>2.5×10^9
5i, k-m	*Vcan-Cre-2A-GFP*<br>*flex-shScramble-GFP*	1.2×10^9<br>2.5×10^9
5j-m	*Vcan-Cre-2A-GFP*<br>*flex-shKiss1r-GFP*	1.2×10^9<br>2.5×10^9
5n, p-r	*A2m-Cre-2A-GFP*<br>*flex-shScramble-GFP*	1.1×10^9<br>2.5×10^9
5o-r	*A2m-Cre-2A-GFP*<br>*flex-shKiss1r-GFP*	1.1×10^9<br>2.5×10^9
5s, t, u	*Dio2-Cre*<br>*flex-shScramble-GFP*	1.25×10^9<br>2.5×10^9

	Dio2-Cre<br>*flex-shKiss1r-GFP*	1.25 x 10^{9}<br>2.5 x 10^{9}
5, v, w, x	*Vcan-Cre-2A-GFP*<br>*flex-shScramble -GFP*	1.2 x 10^{9}<br>2.5 x 10^{9}
	Vcan-Cre-2A-GFP<br>*flex-shKiss1r-GFP*	1.2 x 10^{9}<br>2.5 x 10^{9}
5y, z, aa	*A2m-Cre-2A-GFP*<br>*flex-shScramble-GFP*	1.1 x 10^{9}<br>2.5 x 10^{9}
	A2m-Cre-2A-GFP<br>*flex-shKiss1r-GFP*	1.1 x 10^{9}<br>2.5 x 10^{9}
Supplementary 1i	*Sprr1a-Cre-2A-GFP*	2 x 10^{8}
	Dio2-Cre-2A-GFP	5 x 10^{8}
Supplementary 1k	*EF1a-flex-GPHN-FingR-eGFP*	5 x 10^{9}
Supplementary 1l	*EF1a-flex-PSD95-FingR-eGFP*	5 x 10^{9}
Supplementary 2 b-k, n-s	*Ephb1-Cre-2A-GFP*	1 x 10^{9}
Supplementary 2n - t	*Ephb1-Cre-2A-GFP*	1 x 10^{9}
Supplementary 5a	*CMV-flex-mCherry*	2.3 x 10^{9}
Supplementary 9 b	*Vcan-Cre-2A-GFP*	1 x 10^{9}
	Sprr1a-Cre-2A-GFP	7.2 x 10^{8}
	Pttg1-Cre-2A-GFP	5 x 10^{8}
	Abhd11-Cre-2A-GFP	5 x 10^{8}
	A2m-Cre-2a-GFP	1 x 10^{9}
	Trhde-Cre-2A-GFP	1.5 x 10^{9}
	Scn7a-Cre-2A-GFP	2.2 x 10^{8}
	Cd59a-Cre-2A-GFP	0.8 x 10^{10}
	Crlf3-Cre-2A-GFP	3.2 x 10^{9}
	Slc17a8-Cre-2A-GFP	4.4 x 10^{8}
	Adm-Cre-2A-GFP	5 x 10^{8}
	Crym-Cre-2A-GFP	0.8 x 10^{10}
	Trhr-Cre-2A-GFP	1 x 10^{8}
	Lyz2-Cre-2A-GFP	6.2 x 10^{9}
	Pygm-Cre-2A-GFP	5 x 10^{8}
	Frzb-Cre-2A-GFP	1 x 10^{10}
	Col25a1-Cre-2A-GFP	7.2 x 10^{9}
	Ephb1-Cre-2A-GFP	5 x 10^{8}
	Cers6-Cre-2A-GFP	1.4 x 10^{9}
	Dio2-Cre-2A-GFP	2 x 10^{9}
Supplementary 10a	*Vcan-Cre-2A-GFP*	1 x 10^{9}
	A2m-Cre-2a-GFP	1 x 10^{9}
Supplementary 11 c-f	*AAV9-flex-hM3Dq-mCherry*<br>*Dio2-Cre*<br>*flex-shScramble-GFP*	0.3 x 10^{10}<br><br>1.25 x 10^{9}<br>2.5 x 10^{9}
Supplementary 11b	*Dio2-Cre*	1.25 x 10^{9}
Supplementary 13c	*Dio2-Cre*<br>*flex-shScramble-GFP*	1.25 x 10^{9}<br>2.5 x 10^{9}
	Dio-Cre<br>*flex-shKiss1r-GFP*	1.25 x 10^{9}<br>2.5 x 10^{9}
Supplementary 13d-g, q-t	*Dio2-Cre*<br>*flex-shScramble-GFP*	1.25 x 10^{9}<br>2.5 x 10^{9}
	Dio-Cre<br>*flex-shKiss1r-GFP*	1.25 x 10^{9}<br>2.5 x 10^{9}
	Vcan-Cre-2A-GFP<br>*flex-shKiss1r-GFP*	1.2 x 10^{9}<br>2.5 x 10^{9}
	A2m-Cre-2A-GFP<br>*flex-shKiss1r-GFP*	1.1 x 10^{9}<br>2.5 x 10^{9}
Supplementary 13h-j	*Dio2-Cre*<br>*flex-shScramble-GFP*	1.25 x 10^{9}<br>2.5 x 10^{9}
	Dio2-Cre	1.25 x 10^{9}

	flex-shKiss1r-GFP	2.5×10^9
Supplementary 13k-m	*Vcan-Cre-2A-GFP*<br>*flex-shScramble-GFP*	1.2×10^9<br>2.5×10^9
	Vcan-Cre-2A-GFP<br>*flex-shKiss1r-GFP*	1.2×10^9<br>2.5×10^9
Supplementary 13n-p	*A2m-Cre-2A-GFP*<br>*flex-shScramble-GFP*	1.1×10^9<br>2.5×10^9
	A2m-Cre-2A-GFP<br>*flex-shKiss1r-GFP*	1.1×10^9<br>2.5×10^9
Supplementary 13 z-ae	*A2m-Cre-2A-GFP*<br>*flex-shScramble-GFP*	1.1×10^9<br>2.5×10^9
	A2m-Cre-2A-GFP<br>*flex-shKiss1r-GFP*	1.1×10^9<br>2.5×10^9
Supplementary Video 8	*flex-mGFP*	1.01×10^8
