# Supplementary material for: A synaptoid connectome differentiates tanycytic subpopulations and underlies neuroglial communication and neuroendocrine regulation
Source: Nat Commun. 2026 Jun 20;17:7797. doi: 10.1038/s41467-026-74598-5 (PMC13439102; doi:10.1038/s41467-026-74598-5)
Supplement: Supplementary file 9 [file 41467_2026_74598_MOESM7_ESM.pdf]

Supplementary Data file 5. Sample size, sex of the animals and results of statistical tests. F, female; M, male.

Figure	Sample size (n) and sex (M, male; F, female)	Statistical test	Values
1a, b	2 mice, F	-	-
1c	see Methods section for details	-	-
1d	66 mice, mixed sex	-	-
1e	10 mice, mixed sex	-	-
1f	5 mice, mixed sex	-	-
1g	5 mice, mixed sex	-	-
1h	5 mice, mixed sex	-	-
1i	3 mice, mixed sex	-	-
1j, k,<br><br>Supplementary Fig. 3	8 mice, mixed sex	-	-
1l	5 mice, mixed sex	-	-
2b – h	9 mice,<br>6 F, 3 M	-	-
3a	1 M	-	-
3b	1 F	-	-
3c	1 F	-	-
3e1	9 mice, 5M, 4F	Two-tailed Welch's t-test	F/F_0 (Max) Male vs Female: *T* (4.6) = 0.9, *p* = .3979
3e2	9 mice, 5M, 4F	Two-tailed Welch's t-test	F/F_0 (Max) Male vs Female: *T* (4.3) = 1.9, *p* = .12
3f	9 mice; 5M, 4F	-	-
3g	9 mice; 5M, 4F	RM two-way ANOVA followed by Sidak´s post-hoc tests	Treatment: *F* (1, 7) = 3.6, *p* = .099;<br>Medium: *F* (1, 7) = 26.9, *p* = .001;<br>Interaction: *F* (1, 7) = 18, *p* = .004<br><br>Vehicle: aCSF vs. Kiss10 *p* = .002; Blocker: aCSF vs. Kiss10: *p* = .213; aCSF: Vehicle vs. Blocker: *p* = .052; Kiss10: Vehicle vs. Blocker: *p* = .018
3h	9 mice; 5M, 4F	RM two-way ANOVA followed by	Treatment: *F* (1, 7) = 7.5, *p* = .029;

		Sidak´s post-hoc tests	Medium: F (1, 7) = 22.5, p = .002;<br>Interaction: F (1, 7) = 10.8, p = .013<br><br>Vehicle: aCSF vs. Kiss10 p = .003; Blocker: aCSF vs. Kiss10: p = .952; aCSF: Vehicle vs. Blocker: p = .841; Kiss10: Vehicle vs. Blocker: p = .009
3i3	9 mice, 5M, 4F	Scheirer-Ray-Hare followed two-tailed Wilcoxon matched-pairs signed rank test	Subtype: χ^2 (1) = 6.0, p = .015;<br>Treatment: χ^2 (1) = 7.7, p = .006;<br>Interaction: χ^2 (1) = 5.9, p = .006<br><br>alpha: p = .008;<br>beta: p = .008
3j3	9 mice, 5M, 4F	Scheirer-Ray-Hare followed by two-tailed Wilcoxon matched-pairs signed rank test	Subtype: χ^2 (1) = 5.1, p = .025;<br>Treatment: χ^2 (1) = 7.0, p = .008;<br>Interaction: χ^2 (1) = 3.4, p = .065<br><br>alpha: p = .016;<br>beta: p = .016
3k3	4 mice, mixed sex	RM ANOVA followed by Sidak´s post-hoc tests	Subtype: F (1, 6) = 0.1, p = 0.738<br>Treatment: F (1, 6) = 41.2, p = .0005;<br>Interaction: F (1, 6) = 0.04, p = .856<br><br>alpha: p = .003;<br>beta: p = .002
3l3	8 mice with taltirelin, 9 mice with aCSF Mixed sex	Scheirer-Ray-Hare followed by two-tailed Mann-Whitney-U tests	Subtype: χ^2 (1) = 3.6, p = .056;<br>Treatment: χ^2 (1) = 5.6, p = .018;<br>Interaction: χ^2 (1) = 8.1, p = .004<br><br>alpha: p = .76;<br>beta: p < .000165
3m3	7 mice with carbachol. 9 mice with aCSF Mixed sex	Scheirer-Ray-Hare followed by two-tailed Mann-Whitney-U tests	Subtype: χ^2 (1) = 8.4, p = .004;<br>Treatment: χ^2 (1) = 12.6, p = .0004;

			Interaction: χ^2 (1) = 1.6, p = .211<br><br>alpha: p = .00035;<br>beta: p = .00035
3n3	4 mice with SST, 3 mice with aCSF<br>Mixed sex	Scheirer-Ray-Hare followed by two-tailed Mann-Whitney-U tests	Subtype: χ^2 (1) = 3.3, p = .25;<br>Treatment: χ^2 (1) = 5.4, p = .02;<br>Interaction: χ^2 (1) = 0.6, p = .434<br><br>alpha: p = .111;<br>beta: p = .111
3o3	11 mice<br>Mixed sex	RM ANOVA followed by Sidak´s post-hoc tests	Subtype: F (1, 20) = 2.3, p = .147;<br>Treatment: F (1, 20) = 12.4, p = .002;<br>Interaction: F (1, 20) = 0.9, p = .362;<br><br>alpha: p = .002;<br>beta: p = .019
3p3	13 mice with epinephrine, 11 mice with aCSF<br>Mixed sex	Scheirer-Ray-Hare followed by two-tailed Mann-Whitney-U tests	Subtype: χ^2 (1) = 2.8, p = .097;<br>Treatment: χ^2 (1) = 14.9, p = .0001;<br>Interaction: χ^2 (1) = 1.1, p = .301<br><br>alpha: $p = 6*10^{-5}$;<br>beta: $p = 6*10^{-5}$
4a	66 mice,<br>mixed sex	-	-
4b	47 mice,<br>mixed sex	-	-
4c	41 mice,<br>mixed sex	-	-
4e, f-i	9 mice, 5 F, 4 M	-	-
4g	9 mice/group,<br>Mixed sex	Two-tailed Mann-Whitney U test	U = 7, p = .002
5a<br><br>Supplementary Fig. 12a	5 mice/group,<br>All F	RM two-way ANOVA followed by Sidak´s post-hoc tests	Time: F (13, 52) = 5.9,<br>p = .0000016;<br>Treatment: F (1, 4) = 3.5,<br>p = .13;<br>Interaction: F (13, 52) = 4.1,<br>p = .0001;<br><br>min 0: p = 0.984<br>min 10: p = 0.996<br>min 20: p > 0.999<br>min 30: p = 0.998<br>min 40: p = 0.971<br>min 50: p = 0.961<br>min 60: p = 0.332<br>min 70: p = 0.0003

			min 80: p = 0.335<br>min 90: p = 0.894<br>min 100: p > 0.999<br>min 110: p > 0.999<br>min 120: p = 0.991<br>min 130: p = 0.01
5b<br><br>Supplementary Fig. 12b	6 mice/group<br>All F	RM two-way ANOVA followed by Sidak´s post-hoc tests	Time: F (13, 65) = 87.1, $p <$ $1*10^{-15}$;<br>Treatment: F (1, 5) = 0.1, p = .734;<br>Interaction: F (13, 65) = 0.6, p = .854<br><br>min 0: p = 0.999<br>min 10: p > 0.999<br>min 20: p > 0.999<br>min 30: p > 0.999<br>min 40: p > 0.999<br>min 50: p = 0.978<br>min 60: p > 0.999<br>min 70: p = 0.999<br>min 80: p > 0.999<br>min 90: p > 0.999<br>min 100: p = 0.384<br>min 110: p > 0.999<br>min 120: p > 0.999<br>min 130: p > 0.999
5c<br><br>Supplementary Fig. 12c	6 mice/group<br>All F	RM two-way ANOVA followed by Sidak´s post-hoc tests	Time: F (13, 65) = 22.5, $p <$ $1*10^{-15}$;<br>Treatment: F (1, 5) = 3.9, p = .105;<br>Interaction: F (13, 65) = 1.6, p = .123<br><br>min 0: p = 0.710<br>min 10: p > 0.999<br>min 20: p > 0.999<br>min 30: p > 0.999<br>min 40: p = 0.001<br>min 50: p = 0.052<br>min 60: p > 0.999<br>min 70: p = 0.998<br>min 80: p > 0.999<br>min 90: p > 0.999<br>min 100: p = 0.993<br>min 110: p = 0.830<br>min 120: p = 0.980<br>min 130: p > 0.999
5f	shScram: 9 mice, shKiss1R: 12 mice<br>All F	Two-tailed Mann-Whitney U test	U = 14, p = .0031
5g	shScram: 8 mice, shKiss1R: 12 mice<br>All F	Two-tailed unpaired t test	T (18) = 2.2, p = .045
5h	shScram: 7 mice, shKiss1R: 12 mice<br>All F	Two-tailed Mann-Whitney U test	U = 25, p = .167

5k	shScram: 9 mice, shKiss1R: 8 mice All F	Two-tailed Mann-Whitney U test	*U* = 17, *p* = .052
5l	shScram: 9 mice, shKiss1R: 8 mice All F	Two-tailed unpaired t test	*T (*15) = 1.0, *p* = .322
5m	8 mice/group All F	Two-tailed Mann-Whitney U test	*U* = 15, *p* = .083
5p	7 (Scram), 9 (A2m), All F	Two-tailed unpaired t test	*T (*14) = 3.1, *p* = .009
5q	7 (Scram), 9 (A2m), All F	Two-tailed unpaired t test	*T (*14) = 2.9, *p* = .011
5r	5 (Scram), 7 (A2m, two outliers removed (ROUT test, Q=1%)), All F	Two-tailed Welch's t-test	*T (4.82*) = 3.6, *p* = .018
5t	11 (Scram) mice, 12 (Dio2) mice All F	Two-tailed Mann-Whitney U test	*U* = 10, *p* = .0002
5u	11 (Scram) mice, 12 (Dio2) mice All F	Two-tailed Multiple Mann-Whitney U tests (Holm Sidak corrected)	Estrus: *U* = 26, *p* = .0104; Diestrus: *U* = 27, *p* = .0111; Proestrus: *U* = 39, *p* = .0869;
5w	9/group*, mice* All F	Two-tailed unpaired t-test	*T* (1) = 2.1*, p* = .051
5x	9/group*,* mice All F	Two-tailed Multiple Mann-Whitney U tests (Holm Sidak corrected)	Estrus: *U* = 25, *p* = .1820; Diestrus: *U* = 30, *p* = .3285; Proestrus: *U* = 25, *p* = .1696;
5z	14 (Scram), 12 (A2m) All F	Two-tailed Mann-Whitney U test	*U* = 32, *p* = .004
5aa	14 (Scram), 12 (A2m) All F	Two-tailed Multiple Mann-Whitney U tests (Holm Sidak corrected)	Estrus: *U* = 32, *p* = .0054; Diestrus: *U* = 50, *p* = .0742; Proestrus: *U* = 32, *p* = .0043;
Supplementary Fig. 1a	3 batches of primary tanycytes	-	-
Supplementary Fig. 1b	1 batch of primary tanycytes	-	-
Supplementary Fig. 1c, e, f, j	3 mice, all M	-	-
Supplementary Fig. 1d, g, h, j	3 mice, all M	-	-
Supplementary Fig. 1k	3 biological replicates	-	-
Supplementary Fig. 1i	3 biological replicates	-	-
Supplementary Fig. 2b, c	5 biological replicates	-	-
Supplementary Fig. 2d, e	5 biological replicates	-	-

Supplementary Fig. 2f, g	5 biological replicates	-	-
Supplementary Fig. 2h, i	5 biological replicates	-	-
Supplementary Fig. 2j, k	5 biological replicates	-	-
Supplementary Fig. 2l, m	7 ROIs/ group	Two-tailed Kruskal-Wallis followed by Dunn´s post hoc tests	χ^2 (4) = 15.1, *p* = .0017<br>SHANK2-HOMER1 vs. SHANK2-MCT8:<br>*p* > .999;<br>SHANK2-HOMER1 vs. NLGN2 - MCT8:<br>*p* > .999;<br>SHANK2-HOMER1 vs. Neg. control SHANK2: *p* = 0.001<br>SHANK2 - MCT8 vs. NLGN2 - MCT8:<br>*p* > .999;<br>SHANK2 - MCT8 vs. Neg. control SHANK2: *p* = 0.042<br>NLGN2 - MCT8 vs. Neg. control SHANK2: *p* = 0.031
Supplementary Fig. 4a, b, d	6 F, 3 M	-	-
Supplementary Fig. 4c	6 F, 3 M	Two-tailed Mann-Whitney U-test, with multiple comparisons False Discovery Rate (Q, 1%)	Rt: *U* = 6, *p* = .333<br>Amyg: *U* = 8, *p* =.881<br>PVA: *U* = 6, *p* = .5238<br>Cortex: *U* = 3, *p* = .1666<br>BST: *U* = 3, *p* = .1666<br>PeF: *U* = 8, *p* >0.999<br>f: *U* = 6, *p* = .5238<br>Pr: *U* = 6, *p* = .3333<br>MVePC: *U* = 9, *p* >0.999<br>PBN: *U* = 8, *p* = .8214<br>Bar: *U* = 3, *p* = .0833<br>DPO: *U* = 9, *p* >0.999<br>Tg: *U* = 6, *p* = .5238<br>LC: *U* = 8, *p* = .8452<br>3N: *U* = 7, *p* = .6428<br>EW: *U* = 9, *p* = .9642<br>SNR: *U* = 8, *p* >0.999<br>PAG: *U* = 9, *p* = .9642<br>RMg: *U* = 9, *p* >0.999<br>Ra: *U* = 6, *p* = .5476<br>S: *U* = 8, *p* >0.999<br>CA3: *U* = 6, *p* = .5476<br>CA2: *U* = 4, *p* = .2023<br>CA1: *U* = 3, *p* = .1666<br>SFO: *U* = 2, *p* = .0952<br>ME: *U* = 6, *p* = .5238<br>Ce: *U* = 8, *p* >0.999<br>PSTh: *U* = 6, *p* = .5<br>TC: *U* = 8, *p* = .9047<br>AHP: *U* = 3, *p* = .1666<br>Spa: *U* = 8, *p* = .8214<br>AC: *U* = 9, *p* >0.999<br>MM: *U* = 6, *p* = .5476<br>PH: *U* = 3, *p* = .1071<br>Arc: *U* = 3, *p* = .1666<br>VMH: *U* = 7, *p* = .6666<br>DMH: *U* = 4, *p* = .2619

			Pe: *U* = 8, *p* = .9047<br>PVN: *U* = 2, *p* = .0952<br>LH: *U* = 7, *p* = .7142<br>LA: *U* = 4, *p* = .2381<br>RCh: *U* = 9, *p* >0.999<br>SCh: *U* = 7, *p* = .5952<br>A14: *U* = 8, *p* = .8571<br>AVPe: *U* = 7, *p* = .7142<br>VOLT: *U* = 8, *p* = .8809<br>POA: *U* = 2, *p* = .0952<br>LS: *U* = 6, *p* = .5476<br>VDB: *U* = 6, *p* = .5476<br>HDB: *U* = 3, *p* = .1190<br>MS: *U* = 6, *p* = .4167
Supplementary Fig. 4e, f, h	5 F, 4 M	-	-
Supplementary Fig. 4g	5 F, 4 M	Two-tailed Mann-Whitney U-test, with multiple comparisons False Discovery Rate (Q, 1%)	AD: *U* = 8, *p* = .4444<br>Rt: *U* = 5, *p* = .1666<br>Amyg: *U* = 4, *p* =.1825<br>PVA: *U* = 6, *p* = .3016<br>Cortex: *U* = 7, *p* = .5635<br>BST: *U* = 8, *p* = .7302<br>PeF: *U* = 8, *p* >0.999<br>f: *U* = 7, *p* = .4921<br>Pr: *U* = 10, *p* >0.999<br>MVePC: *U* = 10, *p* >0.999<br>PBN: *U* = 6, *p* = .2857<br>Bar: *U* = 8, *p* = .4444<br>DPO: *U* = 10, *p* >0.999<br>Tg: *U* = 5, *p* = .2777<br>LC: *U* = 10, *p* >0.999<br>3N: *U* = 8, *p* = .6032<br>EW: *U* = 3, *p* = .0476<br>SNR: *U* = 7, *p* = .4444<br>PAG: *U* = 8, *p* = .7302<br>RMg: *U* = 7, *p* = 5238<br>Ra: *U* = 6, *p* = .4127<br>S: *U* = 10, *p* >0.999<br>CA3: *U* = 8, *p* = .555<br>CA2: *U* = 9, *p* = .9206<br>CA1: *U* = 7, *p* = .5<br>SFO: *U* = 8, *p* = .7302<br>ME: *U* = 10, *p* >0.999<br>Ce: *U* = 8, *p* >0.999<br>PSTh: *U* = 5, *p* = .1666<br>TC: *U* = 9, *p* = .9047<br>AHP: *U* = 9, *p* = .9047<br>Spa: *U* = 6, *p* = .4444<br>AC: *U* = 6, *p* = .3651<br>MM: *U* = 9, *p* = .9048<br>PH: *U* = 8, *p* = .7302<br>Arc: *U* = 10, *p* >0.999<br>VMH: *U* = 7, *p* = .5555<br>DMH: *U* = 8, *p* = .7302<br>Pe: *U* = 7, *p* = .5555<br>PVN: *U* = 5, *p* = .2857<br>LH: *U* = 6, *p* = .4127<br>LA: *U* = 4, *p* = .1746<br>RCh: *U* = 1, *p* = .0317<br>SCh: *U* = 7, *p* = .4524<br>A14: *U* = 6, *p* = .2857

			AVPe: $U = 6$, $p = .4126$<br>VOLT: $U = 8$, $p > 0.999$<br>POA: $U = 10$, $p > 0.999$<br>LS: $U = 8$, $p = .7302$<br>VDB: $U = 8$, $p = .5555$<br>HDB: $U = 8$, $p = .6984$<br>MS: $U = 9$, $p = .7936$
Supplementary Fig. 5a	13 mice, mixed sex	-	-
Supplementary Fig. 5b, d, f<br><br>Supplementary Fig. 6a, b, d, h, j	1 M	-	-
Supplementary Fig. 5c, e, g<br><br>Supplementary Fig. 6c, e, f, g, i, k, l, m	1 F	-	-
Supplementary Fig. 7b-f	1 M	-	-
Supplementary Fig. 8b	24 (Control), 61 (Carbachol), 50 (CRH), 34 (Kp10), 9 (OXT), 71 (AVP) cells	Two-tailed Kruskal-Wallis followed by targeted Dunn´s post hoc tests	χ^2 (5) = 32.3, $p < .001$;<br>Control vs. Carbachol: $p = .0006$;<br>Control vs. CRH: $p = .03$;<br>Control vs. Kp10: $p = .000003$;<br>Control vs. OXT: $p = .0002$;<br>Control vs. AVP: $p = .00004$
Supplementary Fig. 8d	4 mice, mixed sex	-	-
Supplementary Fig. 8e-g	10 (Carbachol), 3 (Atropine), 5 (MT7) mice, mixed sex	Two-tailed Kruskal-Wallis followed by Dunn´s post hoc tests	χ^2 (2) = 13.0, $p = .000015$;<br>Carbachol vs. Carbachol + Atropine: $p = .008$;<br>Carbachol vs. Carbachol + MT7: $p = .017$;<br>Carbachol + Atropine vs. Carbachol + MT7: $p > .999$
Supplementary Fig. 8h-k	10 (Carbachol), 4 (2APB), 4 (U73122), 4 (*Gαq11*^{gliaKO}) mice, mixed sex	Two-tailed Kruskal-Wallis followed by Dunn´s post hoc tests	χ^2 (3) = 17.4, $p = .0006$;<br>Carbachol vs. Carbachol+2APB: $p = .003$;<br>Carbachol vs. Carbachol+U73122: $p = .009$;<br>Carbachol vs. Carbachol+Gq11 KO: $p = .305$;<br>Carbachol+2APB vs. Carbachol+U73122: $p > .999$;<br>Carbachol+2APB vs. Carbachol+Gq11 KO: $p > .999$;<br>Carbachol+U73122 vs. Carbachol+Gq11 KO: $p > .999$
Supplementary Fig. 8m	8 mice, mixed sex	-	-
Supplementary Fig. 8n	6 mice, mixed sex	-	-

Supplementary Fig. 8o	6 mice, mixed sex	-	-
Supplementary Fig. 8p, q	11 mice, mixed sex	-	-
Supplementary Fig. 8s	7/group, mice, mixed sex	Two-tailed Friedman followed by targeted Dunn´s post hoc tests	χ^2 (2) = 11.1, *p* = .001;<br>B vs. E: *p* = .003;<br>E vs. E+D: *p* = .032
Supplementary Fig. 8t	7/group, mice, mixed sex	Two-tailed Friedman followed by targeted Dunn´s post hoc tests	χ^2 (2) = 14.0, *p* = .000021;<br>B vs. E: *p* = .00037;<br>E vs. E+Q: *p* = .123
Supplementary Fig. 8u	5/group, mice, mixed sex	Two-tailed Friedman followed by targeted Dunn´s post hoc tests	χ^2 (2) = 10.0, *p* = .001;<br>B vs. E: *p* = .003;<br>E vs. E+C: *p* = .228
Supplementary Fig. 8v	11 (Control), 8 (GHRH), 10 (CRH), 5 (Forskolin), wells	Two-tailed Kruskal-Wallis followed by targeted Dunn´s post hoc tests	χ^2 (3) = 20.8, *p* = .0001;<br>Control vs. Forskolin: *p* = .0002;<br>Control vs. GHRH: *p* = .009;<br>Control vs. CRH: *p* = .002
Supplementary Fig. 9b	Vcan: 47 mice with mixed sex	-	-
	Sprr1a: 4 mice with mixed sex		
	Pttg1: 3 mice with mixed sex		
	Abhd: 2 mice with mixed sex		
	A2m: 15 mice with mixed sex		
	Trhde: 2 mice with mixed sex		
	Scn7a: 5 mice with mixed sex		
	Cd59a: 2 mice with mixed sex		
	Crlf3: 2 mice with mixed sex		
	Slc17a8: 3 mice with mixed sex		
	Adm: 6 mice with mixed sex		
	Crym: 2 mice with mixed sex		
	Trhr: 2 mice with mixed sex		
	Lyz2: 3 mice with mixed sex		
	Pygm: 6 mice with mixed sex		
	Frzb: 1 mouse, M		
	Col25a1: 1 mouse, M		
	Ephb: 10 mice with mixed sex		
	Cers6: 4 mice with mixed sex		

	Dio2: 66 mice with mixed sex		
Supplementary Fig. 10	3 replicates of 3 individual mice	-	-
Supplementary Fig. 11a, b	9 replicates, mixed sex	-	-
Supplementary Fig. 11c - e	11 replicates, all F	-	-
Supplementary Fig. 12d	5 F	-	-
Supplementary Fig. 13b	7 (Diestrus), 7 (Proestrus), 6 (Estrus), 6 (OVX), mice All F	Two-tailed Kruskal-Wallis followed by Dunn´s post hoc tests	χ^2 (3) = 17.5, *p* = .00056; Proestrus vs. Estrus: *p* > .999; Proestrus vs. Diestrus: *p* = 0.256; Proestrus vs. OVX: *p* = .00027; Estrus vs. Diestrus: *p* > .999; Estrus vs. OVX: *p* = .028; Diestrus vs. OVX: *p* = .198
Supplementary Fig. 13d	12 (Scrambled), 7 (Vcan), 6 (Dio2), 6 (A2m), mice, all F	One-way ANOVA followed by targeted Dunnett's post hoc tests	*F* (3, 27) = 6.3, *p* = .002; shScram+Dio2 vs. shKiss1R+Vcan: *p* = .033; shScram+Dio2 vs. shKiss1R+Dio2: *p* = .007 shScram+Dio2 vs. shKiss1R+A2m: *p* = .048
Supplementary Fig. 13e	7 (Scrambled), 7 (Vcan), 6 (Dio2), 6 (A2m), mice all F	Mixed effects analysis followed by Sidak´s post-hoc tests	GFP: *F (*1,21) = 31.8, *p* = .000014; AAV: *F (*3,22) = 0.5, *p* = .7; Interaction: *F* (3,21) = 0.8, *p* = .48;<br><br>GFP+ vs. GFP: shScambled-Dio2: *p* = .362; shKiss1R-Dio2: *p* = .042; shKiss1R-Vcan: *p* = .009; shKiss1R-A2m: *p* = .016
Supplementary Fig. 13f	7 (Scrambled), 7 (Vcan), 6 (Dio2), 6 (A2m), mice all F	RM two-way ANOVA followed by targeted Sidak´s post-hoc tests	GFP: *F (*1,22) = 8.5, *p* =.008; AAV: *F (*3,22) = 0.5, *p* = .682; Interaction: *F (*3,22) = 0.9, *p* = .452;<br><br>GFP+ vs. GFP: shScambled-Dio2: *p* = .04; shKiss1R-Dio2: *p* = .454; shKiss1R-Vcan: *p* = .952; shKiss1R-A2m: *p* = .854;
Supplementary Fig. 13g	7 (Scrambled), 7 (Vcan), 6 (Dio2), 6 (A2m), mice all F	RM two-way ANOVA followed by targeted Sidak´s post-hoc tests	GFP: *F (*1,22) = 26.3, *p* = .000039; AAV: *F (*3,22) = 2.7, *p* = .067; Interaction: *F (*3,22) = 1.5, *p* = .249;<br><br>GFP+ vs. GFP: shScambled-Dio2: *p* = .017; shKiss1R-Dio2: *p* = .006; shKiss1R-Vcan: *p* = .057; shKiss1R-A2m: *p* = .891

Supplementary Fig. 13h	9 (Scram), 12 (shKiss 1R), mice, all F	Two-tailed unpaired t test	*T* (19) = 0.5, *p* = .6
Supplementary Fig. 13i	8 (Scram), 12 (shKiss 1R), mice, all F	Two-tailed unpaired t test	*T* (18) = 1.4, *p* = .195
Supplementary Fig. 13j	7 (Scram), 12 (shKiss 1R), mice, all F	Two-tailed unpaired t test	*T* (17) = 1.3, *p* = .22
Supplementary Fig. 13k	9/group, mice, all F	Two-tailed unpaired t test	*T* (16) = 0.7, *p* = .471
Supplementary Fig. 13l	9 (Scram), 8 (shKiss 1R), mice, all F	Two-tailed unpaired t test	*T* (15) = 0.9, *p* = .363
Supplementary Fig. 13m	8/group, mice, all F	Two-tailed unpaired t test	*T* (14) = 0.6, *p* = .585
Supplementary Fig. 13n	7 (Scram), 9 (shKiss 1R), mice, all F	Two-tailed Mann-Whitney U test	*U* = 19, *p* = .211
Supplementary Fig. 13o	7 (Scram), 9 (shKiss 1R), mice, all F	Two-tailed Mann-Whitney U test	*U = 20, p* = .252
Supplementary Fig. 13p	5 (Scram), 9 (shKiss 1R), mice, all F	Two-tailed unpaired t-test	*T* (12) = 0.528, *p* = .607
Supplementary Fig. 13v	10 (Scram), 10 (Dio2), 6 (Vcan), mice, all M	Two-tailed Kruskal-Wallis followed by Dunn´s post hoc tests	χ^2 (2) = 12.2, *p* = .002;<br><br>shScram+Dio2 vs. shKiss1R+Vcan: p = .003; shScram+Dio2 vs. shKiss1R+Dio2: p = .041; shKiss1R+Vcan vs. shKiss1R+Dio2: p = .074
Supplementary Fig. 13w	10 (Scram), 10 (Dio2), 6 (Vcan), mice	Two-tailed Kruskal-Wallis followed by Dunn´s post hoc tests	χ^2 (2) = 11.1, *p* = .004;<br><br>shScram+Dio2 vs. shKiss1R+Vcan: p > .999; shScram+Dio2 vs. shKiss1R+Dio2: p = .007; shKiss1R+Vcan vs. shKiss1R+Dio2: p = .039
Supplementary Fig. 13x	10 (Scram), 10 (Dio2), 6 (Vcan), mice	Two-tailed Kruskal-Wallis test	χ^2 (2) = 4.7, *p* = .102
Supplementary Fig. 13y	10 (Scram), 6 (Vcan), 10 (Dio2), mice	Two-tailed Kruskal-Wallis test	χ^2 (2) = 5.2, *p* = .073
Supplementary Fig. 13z	10 (Scram), 6 (Vcan), 10 (Dio2), mice	One-way ANOVA	*F* (2, 23) = 1.3, *p* = .299
Supplementary Fig. 13aa	7 (Scram), 8 (A2m), mice, all M	Two-tailed Mann-Whitney U-test	*U* = 12, *p* = 0.063
Supplementary Fig. 13ab	7 (Scram), 8 (A2m), mice, all M	Two-tailed Welch's t-test	*T* (9) = 2.4, *p* = 0.04
Supplementary Fig. 13ac	4 (Scram), 8 (A2m), mice, all M	Two-tailed unpaired t test	*T* (10) = 3.2, *p* = 0.009

Supplementary Fig. 13ad	7 (Scram), 8 (A2m), mice, all M	Two-tailed unpaired t test	T (13) = 3.2, p = 0.006
Supplementary Fig. 13ae	7 (Scram), 8 (A2m), mice, all M	Two-tailed unpaired t test	T (13) = 3.2, p = 0.008
